# Synthesis and Anti-Proliferative Effects of Mono- and Bis-Purinomimetics Targeting Kinases

**DOI:** 10.3390/ijms18112292

**Published:** 2017-11-01

**Authors:** Andrea Bistrović, Anja Harej, Petra Grbčić, Mirela Sedić, Sandra Kraljević Pavelić, Mario Cetina, Silvana Raić-Malić

**Affiliations:** 1 Department of Organic Chemistry, Faculty of Chemical Engineering and Technology, University of Zagreb, Marulićev trg 20, HR-10000 Zagreb, Croatia; abistrov@fkit.hr; 2 Department of Biotechnology, Center for High-Throughput Technologies, University of Rijeka, Ulica Radmile Matejčić 2, HR-51000 Rijeka, Croatia; aharej@biotech.uniri.hr (A.H.); petra.grbcic@gmail.com (P.G.); sandrakp@biotech.uniri.hr (S.K.P.); 3 Department of Applied Chemistry, Faculty of Textile Technology, University of Zagreb, Prilaz baruna Filipovića 28a, HR-10000 Zagreb, Croatia; mcetina@ttf.hr

**Keywords:** pyrrolo[2,3-*d*]pyrimidines, purinomimetics, 1,2,3-triazole, anticancer, pancreatic carcinoma (CFPAC-1)

## Abstract

A series of mono-pyrrolo[2,3-*d*]pyrimidines **4a**–**4k**, unsymmetrical bis-purine isosteres **5a**–**5e** and symmetrical bis-pyrrolo[2,3-*d*]pyrimidines **6a** and **6b** connected via di(1,2,3-triazolyl)phenyl linker were synthesized by click chemistry. Whereas mono- **4g** and bis-pseudopurine **5e** showed selective inhibitory activities on cervical carcinoma (HeLa) cells, bis-pyrrolo[2,3-*d*]pyrimidine **6b** exhibited potent and selective anti-proliferative effect in the nanomolar range on pancreatic carcinoma (CFPAC-1) cells. Among these, compound **6b** induced a significant reduction in the expression level of CDK9 (cyclin-dependent kinase 9)/cyclin T1 in CFPAC-1 cells concomitant with attenuation of proliferative signaling mediated by c-Raf (rapidly accelerated fibrosarcoma) and p38 MAP (mitogen-activated protein) kinases. Our findings encourage further development of novel structurally related analog of **6b** to obtain more selective anticancer agent for treating pancreatic cancer.

## 1. Introduction

**Figure 1:**
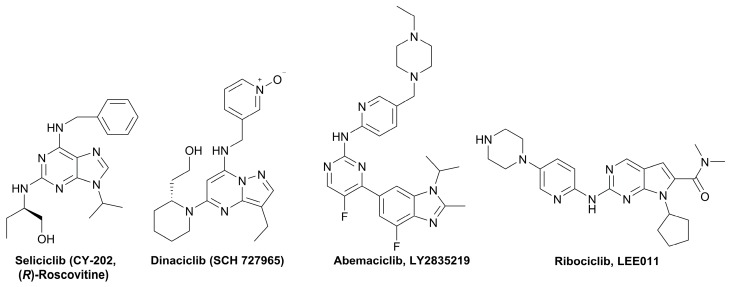
(*R*)-Roscovitine and purine isosteres as small-molecule inhibitors of CDK (cyclin-dependent kinases) under clinical evaluations for the treatment of cancer.

Kinases have emerged as one of the most intensively pursued classes of drug targets with approximately 30 various kinase targets being developed to the stage ready for clinical trials. Among them, cyclin-dependent kinases (CDKs) belong to the serine/threonine kinases with fundamental role in the control of the cell cycle and/or proliferation and transcription [1]. The kinase activity of CDKs is tightly regulated by the binding to cyclins, the levels of which depend on the balance between protein synthesis and proteasomal degradation, which plays an important role in regulating cellular processes [2]. Overexpression of CDK activators such as cyclin D1 is a major cause for the excessive activation of CDKs [3]. Therefore, deregulation of CDK-cyclin activity in cancer cells has provided a rationale for the investigation of CDK inhibitors for therapeutic intervention in various types of cancer. Over the past two decades, several CDK inhibitors have been developed as potential cancer therapeutics and tested in numerous clinical trials for several tumor types [4,5,6]. The first CDK inhibitor, palbociclib, was pyrido[2,3-*d*]pyrimidinone derivative that was selective CDK4 and CDK6 inhibitor approved for the treatment of breast cancer [7,8]. Further investigations on purine and purine-like classes resulted in discovery of the 2,6,9-trisubstituted purine analog, roscovitine [CY-202, *(R*)-roscovitine, seliciclib] that was found to inhibit a subset of CDKs through a direct competition at the ATP-binding site [9,10,11,12]. This purine-based CDK inhibitor was among the first agents evaluated in the clinic [13]. Despite many successful preclinical studies, roscovitine did not meet the initial expectations for a CDK inhibitor in clinical trials [14]. Therefore, purine bioisosteres, as small-molecule CDKs inhibitors, have been widely explored [15,16,17]. Some of them that constitute pyrazolo[1,5-*a*]pyrimidine [18,19,20,21], benzimidazole [22,23] and pyrrolo[2,3-*d*]pyrimidine [24,25,26] scaffolds have been currently under clinical evaluation for the treatment of various cancers (Figure 1).

**Figure 2:**
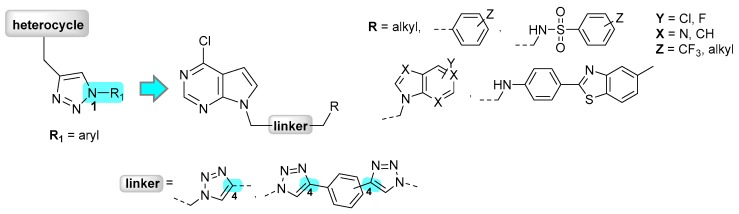
Design and synthesis of mono- and bis-pseudopurines with C-4 substituted 1,2,3-triazole scaffold.

Based on the aforementioned and our results on purinomimetics [27] and pyrimidine [28] with diverse substituents at N-1 of 1,2,3-triazole, we performed structural modification by designing pyrrolo[2,3-*d*]pyrimidines containing alkyl, varied substituted phenyl and phenyl sulfonamide pharmacophores at C-4, instead of N-1, of the 1,2,3-triazole moiety (Figure 2) in order to evaluate their contribution to the anti-proliferative activity. Encouraged by the findings that some heterocyclic dimers with acyclic and cyclic spacer were endowed with pronounced cytostatic activity [29,30,31,32,33,34,35,36] and the importance of halogenated compounds that have been widely exploited in drug discovery [37,38,39] the structural diversity was further extended to the synthesis of halogen-substituted bis-pseudopurines connected through 1,2,3-triazole linker. 1,2,3-Triazole heterocycle has been recognized as good amide bioisosteres and therefore has been widely applied in molecular hybridization approach as an important linker between selected pharmacophoric moieties to produce new hybrid molecules with improved biological properties [40,41,42,43]. Besides unsymmetrical bis-pseudopurine, symmetrical bis-pyrrolo[2,3-*d*]pyrimidines connected via di(ethylene-1,2,3-triazolyl)phenyl spacer were synthesized (Figure 2).

Therefore, a series of mono- (**4a**–**4k**) and bis-pseudopurines (**5a**–**5e**, **6a**, **6b**) were provided with the aim to evaluate their cytostatic activities and further investigate the effects of selected candidates on molecular targets CDK9/cyclin T1, p38 MAPK and c-Raf-1 kinases that regulate cell proliferation.

## 2. Results and Discussion

### 2.1. Chemistry

**Scheme 1:**
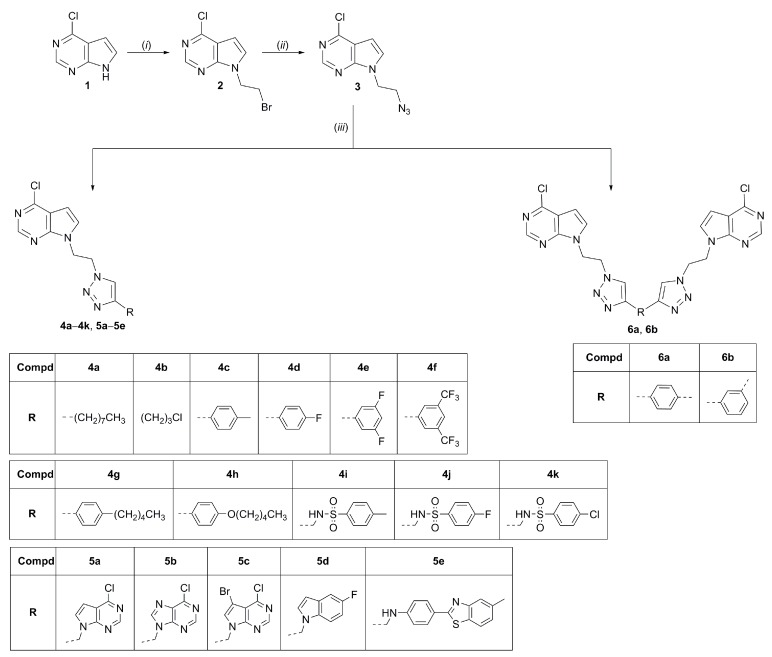
Reagents and conditions: (*i*) 1,2-dibromoethane, DMF (dimethylformamide), Ar atmosphere, rt (room temperature), 24 h; (*ii*) NaN_3_, acetonitrile, reflux, overnight; (*iii*) corresponding terminal alkyne, Cu(0), 1 M CuSO_4_ solution, *tert*-butanol:H_2_O = 1:1, DMF, MW (microwave), 300 W, 80 °C, 45 min. Compd: compound.

At the top of the working area, a focused library of 18 hybrids of mono- and bis-purine isosteres linked via 1,2,3-triazole moiety was prepared as shown in Scheme 1. *N*-Alkylation of 6-chloro-7-deazapurine (4-chloro-pyrrolo[2,3-*d*]pyrimidine) with 1,2-dibromoethane in the presence of K_2_CO_3_ afforded the 2-bromoethyl 7-deazapurine derivative **2**, which was then converted to the key intermediate 7-deazapurine ethyl azide **3** using NaN_3_. Target regioselective 1,4-disubstituted 1,2,3-triazoles (**4a**–**4k**, **5a**–**5e**, **6a** and **6b**) were prepared by Huisgen 1,3-dipolar cycloaddition under microwave irradiation using copper(I) catalyst, that was obtained from copper(II) sulfate and metallic copper. 4-Alkyl- (**4a**, **4b**) and 4-aryl- (**4c**–**4h**) and 4-arylsulfonamide-substituted (**4i**–**4k**) 1,2,3-triazolyl pyrrolo[2,3-*d*]pyrimidine hybrids were obtained by Cu(I)-catalyzed click reaction of corresponding terminal alkynes and 7-deazapurine azide derivative **3**, while bis-purine isosteres (**5a**–**5e**) were prepared using 7-deazapurine azide **3** and *N*-alkynyl heterocyclic base. *N*-Propargylated derivatives of aryl sulfonamides and heterocyclic bases, including 6-chloro-7-deazapurine, 6-chloropurine, 7-bromo-6-chloro-7-deazapurine, 5-fluoroindole and 5-methylbenzothiazole [44,45,46] were prepared according to known procedures given in literature. Symmetrical bis(6-chloro-7-deazapurine-1,2,3-triazol-1-ylethane) derivatives **6a** and **6b** connected through phenyl were prepared were synthesized by Cu(I)-catalyzed 1,3-cycloaddition of 1,4- and 1,3-diethynylbenzene with azide **3**.

### 2.2. X-ray Crystal Structure Analysis

**Figure 3:**
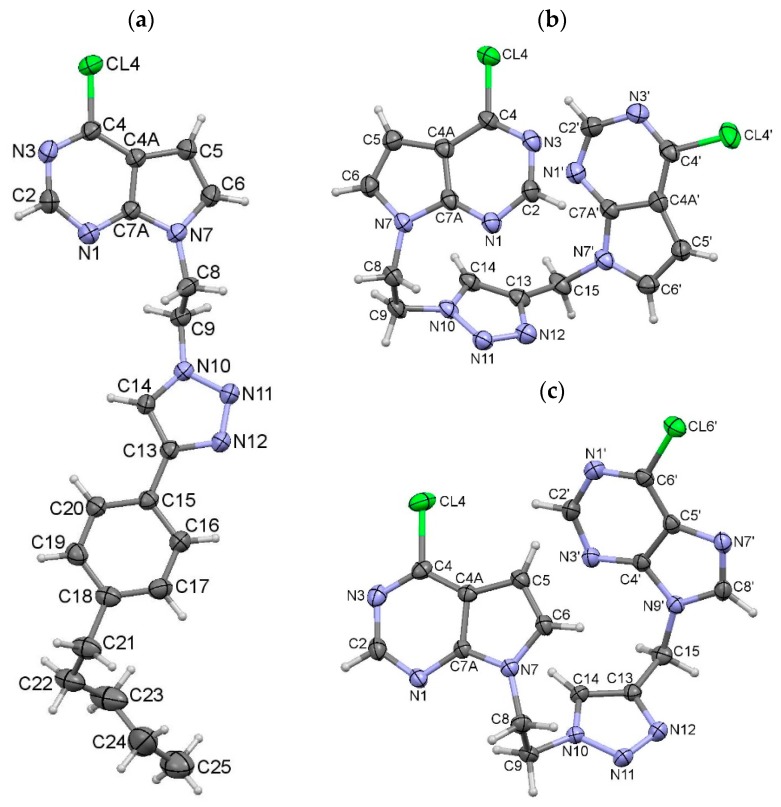
Molecular structures of **4g** (**a**), **5a** (**b**), and **5b** (**c**), with the atom-numbering schemes. Displacement ellipsoids for non-hydrogen atoms are drawn at the 30% probability level. Color code: blue, N; green, Cl; dark grey, C; light grey, H.

The structures of **4g**, **5a** and **5b** are confirmed by single crystal X-ray diffraction method. In **4g**, **5a** and **5b** triazole ring is bonded to the nitrogen N7 atom of the 7-deazapurine ring via ethylene spacer (Figure 3).

The C13 atom of the triazole ring has three different substituents. Pentylphenyl moiety is directly bonded to the triazole ring in **4g**, while 7-deazapurine ring in **5a** and purine ring in **5b** are bonded to the triazole ring via methylene spacer. Equivalent bond lengths in these structures are very similar, but there are some conformational differences between them. Phenyl ring in **4g** is coplanar with the triazole ring; the dihedral angle between the mean planes of the rings is 3.93(13)°. The dihedral angle between the 7-deazapurine and triazole rings in **4g** is slightly bigger (Table S1). On the contrary, 7-deazapurine rings in **5a**, as well as deazapurine and purine rings in **5b** are twisted with respect to the triazole ring for 40° to 70°. It should be mentioned also that 7-deazapurine ring in **5a** is rotated around N7–C8 bond for 187° compared to the ring in **5b**, as defined by the C6–N7–C8–C9 torsion angle (Figure 3b,c; Table S1).

**Figure 4:**
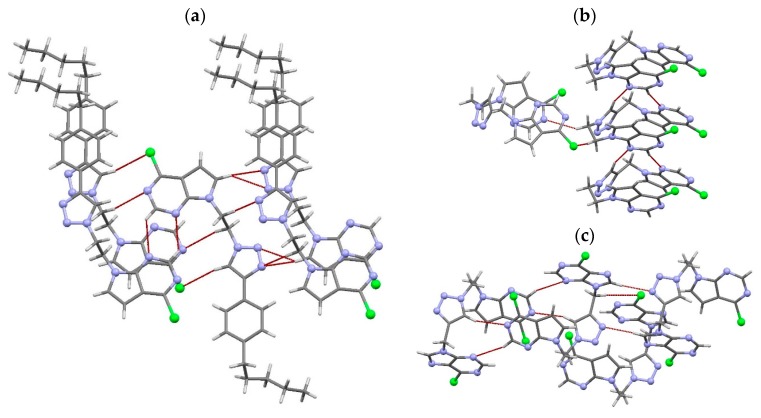
Capped stick representation of **4g** (**a**), **5a** (**b**) and **5b** (**c**), showing C–H···N and C–H···Cl hydrogen bonds. Nitrogen and chlorine atoms are presented in ball and stick style. Color code: blue, N; green, Cl; grey, C; light grey, H.

Weak C–H···N and C–H···Cl hydrogen bonds self-assemble the molecules of **4g**, **5a** and **5b** (Table S2). Thus, six weak C–H···N hydrogen bonds and one C–H···Cl hydrogen bond participate in the supramolecular assembling of **4g** (Figure 4a). Each molecule is linked to four neighboring molecules. The C6 atom is proton donor for two hydrogen bonds, as well as the N12 atom is proton acceptor for two hydrogen bonds. All these interactions form two-dimensional network (Figure S1a). Two π···π interactions established between triazole and phenyl rings of neighboring molecules are also included in the formation of such supramolecular structure (Table S3 and Figure S1b). The molecules of **5a** are linked by three C–H···N hydrogen bonds and one C–H···Cl hydrogen bond (Table S2). Each molecule is linked by these four interactions to other three molecules (Figure 4b), so forming parallel arrangement of hydrogen-bonded chains (Figure S2). Crystal packing diagram reveals that chains are disposed in zig-zag manner and form two-dimensional network (Figure S2). As in **5a**, each molecule of **5b** is connected to three neighboring molecules by three C–H···N hydrogen bonds and one C–H···Cl hydrogen bond (Figure 4c and Table S2). Hydrogen-bonded molecules are mutually parallel, and form two-dimensional network very similar to that in **5a** (Figure S3a). However, there is one distinct difference between these structures. Six-membered N1/C2/N3/C4/C4A/C7A rings of neighboring molecules are mutually parallel, so forming two π···π interactions (Table S3 and Figure S3b). These two interactions extend two-dimensional network into three-dimensional.

### 2.3. Biological Evaluations

#### 2.3.1. Anti-Proliferative Evaluations

Results of anti-proliferative evaluations of compounds **4a**–**4k**, **5a**–**5e**, **6a** and **6b** on human tumor lung adenocarcinoma (A549), ductal pancreatic adenocarcinoma (CFPAC-1), cervical carcinoma (HeLa) and colorectal adenocarcinoma, metastatic (SW620) are presented in Table 1. Roscovitine was used as reference compound. The compound that found to exhibit the highest cytostatic activity and roscovitine were also evaluated on normal human foreskin fibroblasts (HFF).

From the mono-pyrrolo[2,3-*d*]pyrimidines (**4a**–**4k**), compounds containing 4-alkyl (**4a**, **4b**) and 4-aryl-substituted (**4c**–**4f**) 1,2,3-triazole showed only marginal cytostatic effects. It can be observed that type and position of substituents at phenyl ring in **4c**–**4f** had no impact on the growth of tested tumor cell lines. Importantly, *p*-alkyl- and *p*-alkoxyphenyl substituents at C-4 of 1,2,3-triazole in **4g** and **4h**, respectively, considerably improved anti-proliferative effects on CFPAC-1, HeLa and SW620 cells. Thus, 7-deazapurine **4g** with 4-(*p*-pentylphenyl)-1,2,3-triazole exhibited the highest potency (IC_50_ = 9.5 µM) on HeLa cells, whereas its analog **4h** with *p*-pentoxyphenyl substituent at C-4 showed strong activity on CFPAC-1 (IC_50_ = 8.1 µM), HeLa (IC_50_ = 7.8 µM) and SW620 (IC_50_ = 6.9 µM) cells. Interestingly, electron-rich *p*-alkoxy unit in **4g** led to significant improved inhibitory activity on CFPAC-1 cells relative to compound **4h** (IC_50_ > 100 µM). The influence of substituent at phenylsulfonamide was also observed indicating that the electron-withdrawing halogen atoms enhanced the cytostatic effects compared with electron-donating methyl group. Namely, **4i** with *p*-methylphenylsulfonamide was deprived of any cytostatic activity. Conversely, *p*-fluorophenylsulfonamide in **4j** caused slight enhancement of potency on CFPAC-1, HeLa and SW620 cells and *p*-chlorophenylsulfonamide in **4k** increased the activity on all tested cell lines with respect to **4i**. From mono-pyrrolo[2,3-*d*]pyrimidine series (**4a**–**4k**), except for **4f**, compounds **4g** and **4h** with best cytostatic effects showed also the highest Clog*P* (coefficients log*P*) values.

**Figure 5:**
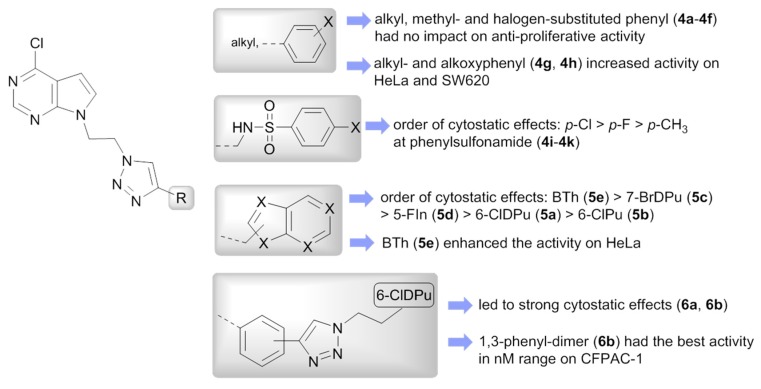
Structure-activity relationship (SAR) of a series of mono- (**4a**–**4k**) and bis-pseudopurines (**5a**–**5e**, **6a** and **6b**).

Among the series of bis-pseudopurines **5a**–**5e**, **6a** and **6b**, the impact of bromine at C-7 of 7-deazapurine (7-BrDPu) in **5c** was detected displaying higher activities of **5c** against all tested cell lines than corresponding analog (7-DPu) **5a** without bromine. These differences in activities were particularly expressed on HeLa and SW620 cells. Bis-purine isosteres bearing 2-aminophenylbenzothiazole (BTh) was endowed with strong and selective anti-proliferative effect (IC_50_ = 5.3 µM) on HeLa cells. On the contrary, 6-chloropurine (6-ClPu) and 5-fluoroindole (5-FIn) moieties in compounds **5b** and **5d**, respectively, decreased their anti-proliferative effects. Symmetrical bis(6-chloro-7-deazapurine-ethyl-1,2,3-triazole) connected via 1,4- and 1,3-phenyl spacer in **6a** and **6b**, respectively, exhibited the most pronounced inhibitory effect, particularly on CFPAC-1 cells (**6a**: IC_50_ = 3.6 µM, **6b**: IC_50_ = 0.95 µM). Abovementioned structural requirements that influenced the cytostatic activities are summarized in Figure 5.

In accord with findings for mono-pyrrolo[2,3-*d*]pyrimidine series (**4a**–**4k**), relationship between lipophilicity and anti-proliferative effect was also observed for bis-pseudopurine series (**5a**–**5e**, **6a** and **6b**) suggesting that compounds **5e**, **6a** and **6b** with best cytostatic effect were rather lipophilic with Clog*P* values from 4.9 to 5.4.

Mono- **4g** and bis-pseudopurine **5e** with selective antitumor activities on HeLa cells, and symmetrical bis-pyrrolo[2,3-*d*]pyrimidines **6b** exhibiting potent and selective cytostatic effect on CFPAC-1 cells were identified as candidates for further biological evaluations in order to investigate their mechanism of action on HeLa and CFPAC-1 cells. In comparison with cytostatic activities of compounds **4g**, **5e** and **6b**, roscovitine exerted markedly weaker anti-proliferative effects on all tested cancer cell lines. However, compound **6b** showing the cytostatic activity in the nanomolar range on CFPAC-1 cells was also cytotoxic to normal fibroblasts. Therefore, additional efforts are required to determine which structural modifications are required to retain anticancer activity and eliminate toxicity of **6b**.

#### 2.3.2. Western Blot Analysis of Predicted Protein Targets

**Figure 6:**
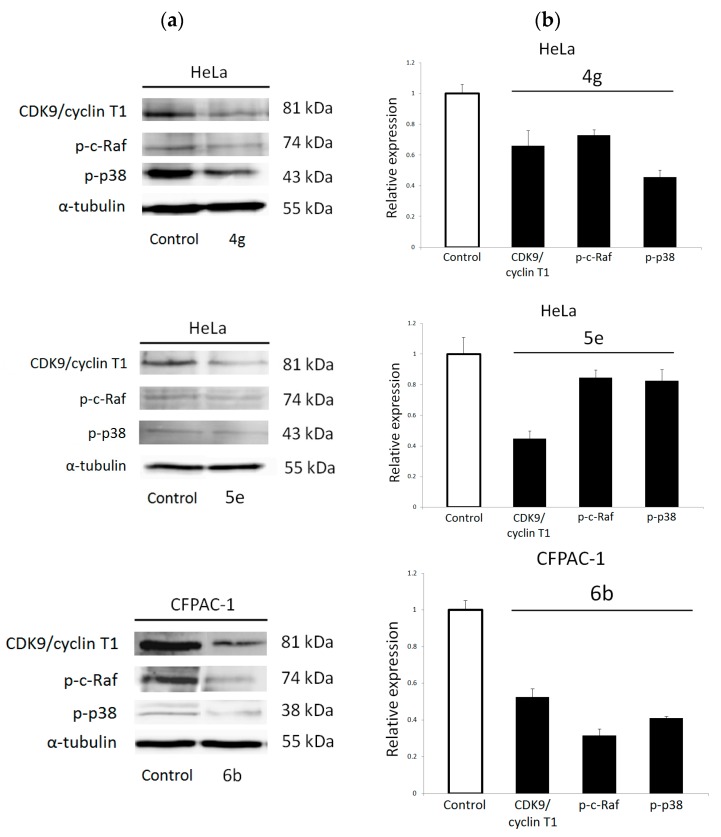
Western blot analysis of predicted protein targets of compounds **4g**, **5e** and **6b**. (**a**) Representative Western blots are shown detecting the cellular levels of selected proteins before and after treatment of the pancreatic adenocarcinoma (CFPAC-1) and HeLa cells with indicated compounds at their 2 × IC_50_ values for 48 h. Approximate molecular weights (kDa) are indicated. (**b**) Relative protein expressions, as determined by densitometric analysis of protein bands and normalized to the α-tubulin loading control. Two independent experiments were performed with similar results. Data are presented as mean values ± SD.

The prediction of activity spectra for substances analysis (PASS) [47] was performed to reveal the probable protein targets of selected candidates **4g**, **5e** and **6b**. Thus, CDK9/cyclin T1 was indicated with the highest probability as a potential target for **4g**, **5e** and **6b** (Tables S4–S6). Western blot analyses (Figure 6) showed that compounds **5e** and **6b** significantly reduced the expression level of CDK9/cyclin T1 in human pancreatic cancer CFPAC-1 cells and human cervical cancer HeLa cells, respectively. In addition, compound **4g** also induced down-regulation of CDK9/cyclin T1, although to a lesser extent. Thus, we can conclude that compounds **4g**, **5e** and **6b** exert their antitumor effects by targeting CDK9/cyclin T1. Similarly, among the most advanced CDK4 and CDK6 inhibitors, purinomimetics abemaciclib (LY2835219) [22,23] and ribociclib (LEE011) (Figure 1) [23,48] showed to be potent CDK9 inhibitors. In addition, the therapeutic potential of selective CDK inhibition on pancreatic cancer cells was revealed by other structurally related small molecule CDK inhibitor, dinaciclib (SCH727965) (Figure 1) [21,49]. Furthermore, PASS analysis also revealed that some of selected compounds could potentially inhibit p38 MAP kinase with higher (compound **6b**) or lower probability (compound **4g**) (Tables S4–S6). For compound **5e**, PASS also revealed the possibility to inhibit MAPK with high probability, and for compounds **4g** and **6b** with lower probabilities. Since c-Raf is a member of MAPK family [50], this protein was tested as a potential target for all three compounds. Previously demonstrated roles of p38 MAPK and c-Raf in promoting cancer cell proliferation [51] provide additional rationale for examining their involvement in mediating anti-proliferative effects of tested compounds revealed by the MTT (3-(4,5-dimethylthiazol-2-yl)-2,5-diphenyltetrazolium bromide) assay.

Our results revealed that symmetrical bis-pyrrolo[2,3-*d*]pyrimidine **6b** elicited significant decrease in the levels of phospho-c-Raf and phospho-p38 kinases, which points to abrogation of their activities (Figure 6). Modest inhibition of c-Raf activity was also observed with mono-pyrrolo[2,3-*d*]pyrimidine **4g** and unsymmetrical bis-purine isostere **5e**. Importantly, **4g** led to a marked reduction in p38 MAPK activity in HeLa cells. These differences in the inhibitory effects on c-Raf and p38 kinases activities between evaluated compounds could be ascribed to both purine isostere and substituent at C-4 of 1,2,3-triazole moiety. It is interesting to note that symmetrical bis(pyrrolo[2,3-*d*]pyrimidine) hybrid **6b** with the most pronounced expression of c-Raf and p38 kinases also showed the most potent cytostatic effects among these compounds as evident from their IC_50_ values (Table 1). Altogether, Western blot study has demonstrated that anti-proliferative effect of compound **6b** in CFPAC-1 cells could be attributed to inhibition of CDK9/cyclin T1 and attenuation of oncogenic signaling propagated by p38 MAPK and c-Raf, which encourages further development of compound **6b** as a novel anticancer agent for treating pancreatic cancer.

#### 2.3.3. Apoptosis Detection

**Figure 7:**
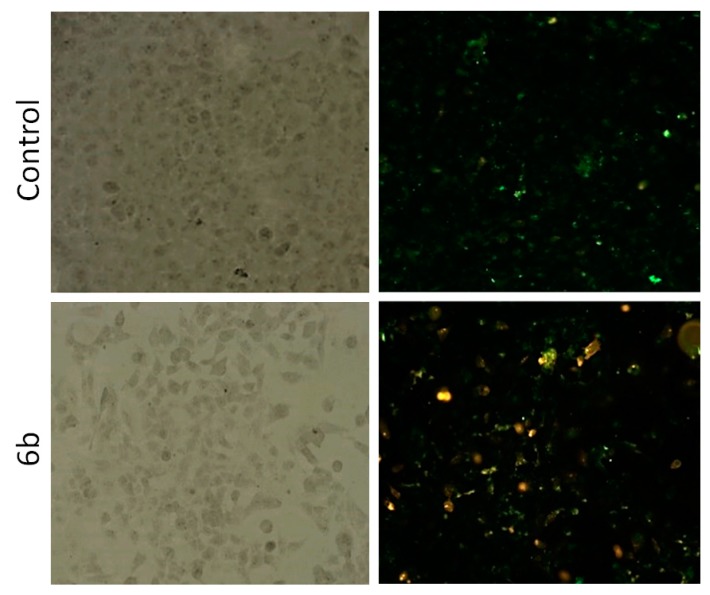
Detection of apoptosis induced by compound **6b** in human pancreatic adenocarcinoma CFPAC-1 cell line using Annexin V-FITC assay. Cells were visualized by fluorescence microscope at 40× magnification before and after treatment with indicated compounds at the concentration of 2 × IC_50_ for 48 h. PI staining was used as a nuclear marker. Shown here are bright-field images (**left**) and late apoptotic/primary necrotic cells (**right**).

Annexin V assay was carried out to determine if the growth-inhibitory activity of compound **6b** exhibiting the most pronounced potency could be attributed to induction of apoptosis. Obtained results (Table 2, Figure 7) showed that compound **6b** reduced viable cell population by 19.48% concurrent with a marked increase in late apoptotic/primary necrotic and secondary necrotic cells by 12.24% and 10.28%, respectively. Therefore, obtained results showed that antitumor effects of compound **6b** in CFPAC-1 cells could be ascribed to its ability to induce late apoptosis and necrosis.

## 3. Materials and Methods

### 3.1. General

All solvents were purified using appropriate drying agents and stored over 3 Å molecular sieves. Thin layer chromatography (TLC) was carried out using Merck silica gel 60F-254 plates (Merck, Darmstadt, Germany), while Fluka 0.063–0.2 mm silica gel (Fluka, Neu-Ulm, Germany) was applied for purification by column chromatography. Melting points (m.p.) were determined on Kofler micro hot-stage (Reichert, Wien, Austria). ^1^H and ^13^C APT (Attached Proton Test) NMR (nuclear magnetic resonance) spectra were recorded in DMSO-*d*_6_ on a Varian Gemini 300 (at 300 and 75 MHz) or Varian Gemini 600 (at 600 and 150 MHz) (Figures S4–S23). Chemical shifts (б) were referenced to the residual solvent signal of DMSO at б 2.50 ppm for ^1^H and б 39.50 ppm for ^13^C. Individual resonances were assigned on the basis of their chemical shifts, signal intensities, multiplicity of resonances and H-H coupling constants. High performance liquid chromatography was performed on an Agilent 1100 series system with UV detection (photodiode array detector). Zorbax C18 reverse-phase analytical column (2.1 × 30 mm, 3.5 µm) was used. All novel compounds showed 95% purity in this HPLC (high performance liquid chromatography) system. Microwave-assisted syntheses were performed in a Milestone start S microwave oven using quartz cuvettes.

### 3.2. Experimental Procedures for the Synthesis of Compounds

*7-(2-Bromoethyl)-4-chloro-7H-pyrrolo[2,3-d]pyrimidine* (**2**). 6-Chloro-7-deazapurine (500 mg; 3.26 mmol) and K_2_CO_3_ (540 mg; 3.91 mmol) were dissolved in 10 mL DMF and stirred under argon atmosphere for 1 h. 1,2-Dibromoethane (0.34 mL; 3.91 mmol) was added to the mixture and stirred for 24 h at room temperature. The solvent was evaporated to dryness and the residue was purified by column chromatography (hexane:ethyl acetate = 8:1) to give compound **1** as white powder (347.38 mg, 41%, m.p. = 91–93 °C). ^1^H NMR (300 MHz, DMSO) δ 8.66 (1H, s, H2), 7.85 (1H, d, *J* = 3.6 Hz, H6), 6.69 (1H, d, *J* = 3.6 Hz, H5), 4.71 (2H, t, *J* = 6.2 Hz, CH_2_), 3.96 (2H, t, *J* = 6.2 Hz, CH).^13^C NMR (151 MHz, DMSO) δ 150.7, 150.6, 150.3, 131.4, 116.8, 98.6, 45.9, 31.6.

*7-(2-Azidoethyl)-4-chloro-7H-pyrrolo[2,3-d]pyrimidine* (**3**). Compound **2** (247.8 mg, 0.95 mmol) and NaN_3_ (167.4 mg, 1.90 mmol) were stirred in acetonitrile (10 mL) under reflux overnight. The solvent was evaporated to dryness and the residue was purified by column chromatography (hexane: ethyl acetate = 1:1) to obtain **3** as crude oil (189.2, 95%). ^1^H NMR (300 MHz, DMSO) δ 8.67 (1H, s, H2), 7.83 (1H, d, *J* = 3.6 Hz, H6), 6.71 (1H, d, *J* = 3.6 Hz, H5), 4.49 (2H, t, *J* = 6.2 Hz, CH_2_), 3.81 (2H, t, *J* = 6.2 Hz, CH_2_). ^13^C NMR (151 MHz, DMSO) δ 151.0, 150.8, 150.5, 131.5, 116.9, 98.9, 50.1, 43.9. 

#### 3.2.1. General Procedure for the Synthesis of Compounds (**4a**–**4k** and **5a**–**5e**)

The azido derivative **3** was dissolved in 0.5 mL DMF and *t*-BuOH:H_2_O = 1:1 (2–3 mL), Cu(0) (0.3 eq.), 1 M CuSO_4_ (0.8 eq.). The corresponding terminal alkyne (1.2 eq.) was added and stirred under microwave irradiation for 45 min at 80 °C and 300 W. The solvent was evaporated and the residue was purified by column chromatography using CH_2_Cl_2_:CH_3_OH = 100:1, as an initial eluent, and CH_2_Cl_2_:CH_3_OH = 10:1, as final eluent.

*4-Chloro-7-(2-(4-octyl-1H-1,2,3-triazol-1-yl)ethyl)-7H-pyrrolo[2,3-d]pyrimidine* (**4a**). Compound **4a** was prepared using the above-mentioned procedure using compound **3** (30 mg, 0.14 mmol) and decyne (0.03 mL, 0.17 mmol) to obtain **4a** as brown solid (13.6 mg, 29%, m.p. = 115–118 °C). ^1^H NMR (600 MHz, DMSO) δ 8.55 (1H, s, H2), 7.55 (1H, s, H5′), 7.52 (1H, d, *J* = 3.6 Hz, H6), 6.59 (1H, d, *J* = 3.6 Hz, H5), 4.84–4.76 (2H, m, CH_2_), 4.75–4.68 (2H, m, CH_2_), 2.46 (2H, t, *J* = 7.4 Hz, CH_2_′′), 1.46–1.38 (2H, m, CH_2_′′), 1.31–1.22 (8H, m, CH_2_′′), 1.17–1.12 (2H, m, CH_2_′′), 0.86 (3H, t, *J* = 7.1 Hz, CH_3_′′). ^13^C NMR (75 MHz, DMSO) δ 153.2, 150.7, 150.3, 147.1, 131.2, 122.2, 116.8, 99.0, 49.0, 44.8, 31.6, 29.0, 28.8, 28.7, 28.4, 24.9, 22.3, 14.1. Anal. calcd. (analytically calculated) for C_18_H_25_ClN_6_: C, 59.91; H, 6.98; N, 23.29. Found: C, 60.13; H, 7.07; N, 23.22.

*4-Chloro-7-(2-(4-(3-chloropropyl)-1H-1,2,3-triazol-1-yl)ethyl)-7H-pyrrolo[2,3-d]pyrimidine* (**4b**). Compound **4b** was prepared using the above-mentioned procedure using compound **3** (30 mg, 0.14 mmol) and 5-chloropent-1-yne (0.02 mL, 0.17 mmol) to obtain **4b** as white crystals (34.9 mg, 77%, m.p. = 114–116 °C). ^1^H NMR (300 MHz, DMSO) δ 8.55 (1H, s, H2), 7.66 (1H, s, H5′), 7.53 (1H, d, *J* = 3.6 Hz, H6), 6.60 (1H, d, *J* = 3.6 Hz, H5), 4.80 (2H, m, CH_2_), 4.77–4.70 (2H, m, CH_2_), 3.54 (2H, t, *J* = 6.5 Hz, CH_2_′′), 2.64 (2H, t, *J* = 7.3 Hz, CH_2_′′), 2.03–1.77 (2H, m, CH_2_′′). ^13^C NMR (75 MHz, DMSO) δ 150.8, 150.7, 150.3, 145.5, 131.2, 122.5, 116.8, 98.9, 49.0, 44.8, 44.5, 31.8, 22.1. Anal. calcd. for C_13_H_14_Cl_2_N_6_: C, 48.01; H, 4.34; N, 25.84. Found: C, 47.86; H, 4.39; N, 25.71.

*4-Chloro-7-(2-(4-(p-tolyl)-1H-1,2,3-triazol-1-yl)ethyl)-7H-pyrrolo[2,3-d]pyrimidine* (**4c**). Compound **4c** was prepared using the above-mentioned procedure using compound **3** (30 mg, 0.14 mmol) and 1-ethynyl-4-methylbenzene (0.02 mL, 0.17 mmol) to obtain **4c** as white powder (15 mg, 31%, m.p. = 198–201 °C). ^1^H NMR (300 MHz, DMSO) δ 8.56 (1H, s, H2), 8.32 (1H, s, H5′), 7.60 (2H, d, *J* = 8.1 Hz, Ph′′), 7.56 (1H, d, *J* = 3.6 Hz, H6), 7.22 (2H, d, *J* = 7.9 Hz, Ph′′), 6.61 (1H, d, *J* = 3.6 Hz, H5), 4.93–4.85 (2H, m, CH_2_), 4.85–4.76 (2H, m, CH_2_), 2.31 (3H, s, CH_3_′′). ^13^C NMR (75 MHz, DMSO) δ 150.8, 150.7, 150.3, 146.5, 137.3, 131.3, 129.5, 127.8, 125.1, 121.3, 116.9, 98.9, 49.2, 44.8, 20.9. Anal. calcd. for C_17_H_15_ClN_6_: C, 60.27; H, 4.46; N, 24.80. Found: C, 60.35; H, 4.43; N, 24.98.

*4-Chloro-7-(2-(4-(4-fluorophenyl)-1H-1,2,3-triazol-1-yl)ethyl)-7H-pyrrolo[2,3-d]pyrimidine* (**4d**). Compound **4d** was prepared using the above-mentioned procedure using compound **3** (30 mg, 0.14 mmol) and 1-ethynyl-4-fluorobenzene (0.02 mL, 0.17 mmol) to obtain **4d** as white crystals (25.8 mg, 54%, m.p. = 200–202 °C). ^1^H NMR (300 MHz, DMSO) δ 8.56 (1H, s, H2), 8.38 (1H, s, H5′), 7.79–7.73 (2H, m, Ph′′), 7.56 (1H, d, *J* = 3.6 Hz, H6), 7.26 (2H, t, *J* = 8.9 Hz, Ph′′), 6.61 (1H, d, *J* = 3.6 Hz, H5), 4.93–4.87 (2H, m, CH_2_), 4.84–4.78 (2H, m, CH_2_). ^13^C NMR (151 MHz, DMSO) δ 162.7; 161.1 (d, *J_CF_* = 244.6 Hz), 150.8, 150.7, 150.3, 145.6, 131.3, 127.3; 127.2 (d, *J_CF_* = 8.2 Hz), 127.2, 121.7, 116.9, 116.0; 115.8 (d, *J_CF_* = 21.7 Hz), 99.0, 49.3, 44.8. Anal. calcd. for C_16_H_12_ClFN_6_: C, 56.07; H, 3.53; N, 24.52. Found: C, 55.83; H, 3.80; N, 24.26.

*4-Chloro-7-(2-(4-(3,5-difluorophenyl)-1H-1,2,3-triazol-1-yl)ethyl)-7H-pyrrolo[2,3-d]pyrimidine* (**4e**). Compound **4e** was prepared using the above-mentioned procedure using compound **3** (30 mg, 0.14 mmol) and 1-ethynyl-3,5-difluorobenzene (0.02 mL, 0.17 mmol) to obtain **4e** as white crystals (37.3 mg, 74%, m.p. = 187–190 °C). ^1^H NMR (300 MHz, DMSO) δ 8.56 (1H, s, H2), 8.52 (1H, s, H5′), 7.57 (1H, d, *J* = 3.6 Hz, H6), 7.47–7.42 (2H, m, Ph′′), 7.27–7.13 (1H, m, Ph′′), 6.61 (1H, d, *J* = 3.6 Hz, H5), 4.96–4.89 (2H, m, CH_2_), 4.84–4.78 (2H, m, CH_2_). ^13^C NMR (75 MHz, DMSO) δ 164.7; 161.4 (d, *J_CF_* = 245.7 Hz) 164.5; 161.3 (d, *J_CF_* = 245.7 Hz), 150.9, 150.7, 150.3, 144.5, 134.1, 131.4, 123.2, 116.9, 108.3; 108.2; 108.1, 108.0 (dd, *J_CF_* = 26.3, 8.6 Hz), 103.6; 103.3; 102.9 (t, *J_CF_* = 25.9 Hz), 99.0, 49.5, 44.8. Anal. calcd. for C_16_H_11_ClF_2_N_6_: C, 53.27; H, 3.07; N, 29.30. Found: C, 53.42; H, 3.01; N, 29.20.

*7-(2-(4-(3,5-Bis(trifluoromethyl)phenyl)-1H-1,2,3-triazol-1-yl)ethyl)-4-chloro-7H-pyrrolo[2,3-d]pyrimidine* (**4f**). Compound **4f** was prepared using the above-mentioned procedure using compound **3** (30 mg, 0.14 mmol) and 1-ethynyl-3,5-bis(trifluoromethyl)benzene to obtain **4f** as white power (60.9 mg, 94%, m.p. = 215–218 °C). ^1^H NMR (300 MHz, DMSO) δ 8.80 (1H, s, H5′), 8.55 (1H, s, H2), 8.39 (2H, s, Ph′′), 8.05 (1H, s, Ph′′), 7.58 (1H, d, *J* = 3.6 Hz, H6), 6.61 (1H, d, *J* = 3.6 Hz, H5), 4.96–4.94 (2H, m, CH_2_), 4.84–4.80 (2H, m, CH_2_). ^13^C NMR (151 MHz, DMSO) δ 150.9, 150.9, 150.4, 143.9, 133.2, 131.6; 131.4; 131.2; 131.0 (q, *J_CF_* = 33.3 Hz), 131.4, 126.1; 124.3; 122.5; 120.7 (q, *J_CF_* = 273.0 Hz), 125.5; 125.4 (d, *J_CF_* = 3.6 Hz), 123.9, 121.4, 121.4; 121.4 (m), 117.0, 99.2, 49.7, 44.9. Anal. calcd. for C_18_H_11_ClF_6_N_6_: C, 46.92; H, 2.41; N, 18.24. Found: C, 47.21; H, 2.16; N, 18.37.

*4-Chloro-7-(2-(4-(4-pentylphenyl)-1H-1,2,3-triazol-1-yl)ethyl)-7H-pyrrolo[2,3-d]pyrimidine* (**4g**). Compound **4g** was prepared using the above-mentioned procedure using compound **3** (30 mg, 0.14 mmol) and 1-ethynyl-4-pentylbenzene (0.03 mL, 0.17 mmol) to obtain **4g** as colorless crystals (40.2 mg, 72%, m.p. = 170–173 °C). ^1^H NMR (300 MHz, DMSO) δ 8.57 (1H, s, H2), 8.33 (1H, s, H5′), 7.62 (2H, d, *J* = 8.1 Hz, Ph′′), 7.54 (1H, d, *J* = 3.7 Hz, H6), 7.23 (2H, d, *J* = 8.1 Hz, Ph′′), 6.60 (1H, d, *J* = 3.6 Hz, H5), 4.92–4.86 (2H, m, CH_2_), 4.84–4.78 (2H, m,CH_2_), 2.61–2.54 (2H, m, CH_2_′′), 1.58 (2H, dt, *J* = 14.5, 7.1 Hz, CH_2_′′), 1.35–1.24 (4H, m, CH_2_′′), 0.86 (3H, t, *J* = 6.8 Hz, CH_3_′′). ^13^C NMR (151 MHz, DMSO) δ 150.9, 150.7, 150.4, 146.6, 142.3, 131.4, 128.9, 128.1, 125.2, 121.4, 116.9, 99.0, 49.2, 44.8, 35.0, 31.0, 30.7, 22.1, 14.1. Anal. calcd. for C_21_H_23_ClN_6_: C, 63.87; H, 5.87; N, 21.28. Found: C, 63.82; H, 6.00; N, 20.96.

*4-Chloro-7-(2-(4-(4-(pentyloxy)phenyl)-1H-1,2,3-triazol-1-yl)ethyl)-7H-pyrrolo[2,3-d]pyrimidine* (**4h**). Compound **4h** was prepared using the above-mentioned procedure using compound **3** (30 mg, 0.14 mmol) and 1-ethynyl-4-(pentyloxy)benzene to obtain **4h** as white powder (49.7 mg, 86%, m.p. = 166–168 °C). ^1^H NMR (300 MHz, DMSO) δ 8.57 (1H, s, H2), 8.26 (1H, s, H5′), 7.62 (2H, d, *J* = 8.7 Hz, Ph′′), 7.55 (1H, d, *J* = 3.6 Hz, H6), 6.96 (2H, d, *J* = 8.8 Hz, Ph′′), 6.60 (1H, d, *J* = 3.6 Hz, H5), 4.91–4.85 (2H, m, CH_2_), 4.83–4.77 (2H, m, CH_2_), 3.98 (2H, t, *J* = 6.5 Hz, CH_2_′′), 1.78–1.65 (2H, m, CH_2_′′), 1.46–1.28 (4H, m, CH_2_′′), 0.90 (3H, t, *J* = 7.0 Hz, CH_2_′′). ^13^C NMR (75 MHz, DMSO) δ 158.5, 150.8, 150.6, 150.3, 146.3, 131.3, 126.5, 123.0, 120.7, 116.8, 114.8, 98.8, 67.5, 49.1, 44.7, 28.4, 27.7, 21.9, 14.0. Anal. calcd. for C_21_H_23_ClN_6_O: C, 61.38; H, 5.64; N, 20.45. Found: C, 61.42; H, 5.55; N, 20.64.

*N-((1-(2-(4-Chloro-7H-pyrrolo[2,3-d]pyrimidin-7-yl)ethyl)-1H-1,2,3-triazol-4-yl)methyl)-4-methylbenzenesulfonamide* (**4i**). Compound **4i** was prepared using the above-mentioned procedure using compound **3** (20 mg, 0.1 mmol) and 4-methyl-*N*-(prop-2-ynyl)benzenesulfonamide (24.07 mg, 0.12 mmol) to obtain **4i** as white powder (30.3 mg, 73%, m.p. = 190–192 °C). ^1^H NMR (600 MHz, DMSO) δ 8.59 (1H, s, H2), 7.98 (1H, t, *J* = 5.6 Hz, NH′′), 7.78 (1H, s, H5′′), 7.65 (2H, d, *J* = 8.2 Hz, Ph′′), 7.46 (1H, d, *J* = 3.6 Hz, H6), 7.37 (2H, d, *J* = 8.1 Hz, Ph′′), 6.59 (1H, d, *J* = 3.6 Hz, H5), 4.79 (2H, t, *J* = 5.8 Hz, CH_2_), 4.71 (2H, t, *J* = 5.7 Hz, CH_2_), 3.92 (2H, d, *J* = 5.5 Hz, CH_2_′′), 2.38 (3H, s, CH_3_′′). ^13^C NMR (151 MHz, DMSO) δ 150.7, 150.5, 150.3, 143.6, 142.6, 137.4, 131.1, 129.5, 126.6, 123.5, 116.7, 98.7, 48.7, 44.5, 38.0, 20.9. Anal. calcd. for C_18_H_18_ClN_7_O_2_S: C, 50.06; H, 4.20; N, 22.70. Found: C, 49.91; H, 4.52; N, 22.33.

*N-((1-(2-(4-Chloro-7H-pyrrolo[2,3-d]pyrimidin-7-yl)ethyl)-1H-1,2,3-triazol-4-yl)methyl)-4-fluorobenzenesulfonamide* (**4j**). Compound **4j** was prepared using the above-mentioned procedure using compound **3** (20 mg, 0.1 mmol) and 4-fluoro-*N*-(prop-2-ynyl)benzenesulfonamide (26.60 mg, 0.12 mmol) to obtain **4j** as white powder (10.1 mg, 24%, m.p. = 178–179 °C). ^1^H NMR (600 MHz, DMSO) δ 8.58 (1H, s, H2), 8.13 (1H, s, NH′′), 7.85–7.79 (3H, m, *J* = 9.5, 6.2 Hz, H5′, Ph′′), 7.47 (1H, d, *J* = 3.5 Hz, H6), 7.40 (2H, t, *J* = 8.8 Hz, Ph′′), 6.59 (1H, d, *J* = 3.5 Hz, H5), 4.79 (2H, t, *J* = 5.7 Hz, CH_2_), 4.72 (2H, t, *J* = 5.8 Hz, CH_2_), 3.96 (2H, s, CH_2_′′). ^13^C NMR (151 MHz, DMSO) δ 164.9; 163.2 (*J_CF_* = 251.0 Hz), 150.7, 150.6, 150.2, 143.4, 136.8; 136.7 (*J_CF_* = 3.4 Hz), 131.1, 129.6, 129.5 (*J_CF_* = 9.5 Hz), 123.6, 116.7, 116.3; 116.1 (*J_CF_* = 22.7 Hz), 98.7, 48.7, 44.5, 37.9. Anal. calcd. for C_17_H_15_ClFN_7_O_2_S: C, 46.85; H, 3.47; N, 22.49. Found: C, 46.87; H, 3.19; N, 22.62.

*N-((1-(2-(4-Chloro-7H-pyrrolo[2,3-d]pyrimidin-7-yl)ethyl)-1H-1,2,3-triazol-4-yl)methyl)-4-chlorobenzenesulfonamide* (**4k**). Compound **4k** was prepared using the above-mentioned procedure using compound **3** (25 mg, 0.11 mmol), and 4-chloro-*N*-(prop-2-ynyl)benzenesulfonamide (28.83 mg, 0.13 mmol) to obtain **4k** as white powder (35.3 mg, 71%, m.p. = 195–197 °C). ^1^H NMR (300 MHz, DMSO) δ 8.59 (1H, s, H2), 8.20 (1H, bs, NH′′), 7.80 (1H, s, H5′), 7.76 (2H, d, *J* = 8.7 Hz, Ph′′), 7.64 (2H, d, *J* = 8.7 Hz, Ph′′), 7.47 (1H, d, *J* = 3.6 Hz, H6), 6.60 (1H, d, *J* = 3.6 Hz, H5), 4.86–4.74 (2H, m, CH_2_), 4.74–4.63 (2H, m, CH_2_), 3.97 (2H, s, CH_2_′′). ^13^C NMR (75 MHz, DMSO) δ 150.8, 150.7, 150.3, 143.4, 139.3, 137.3, 131.2, 129.3, 128.6, 123.7, 116.8, 98.8, 48.9, 44.6, 37.9. Anal. calcd. for C_17_H_15_Cl_2_N_7_O_2_S: C, 45.14; H, 3.34; N, 21.68. Found: C, 44.89; H, 3.46; N, 21.60.

*4-Chloro-7-((1-(2-(4-chloro-7H-pyrrolo[2,3-d]pyrimidin-7-yl)ethyl)-1H-1,2,3-triazol-4-yl)methyl)-7H-pyrrolo*[2,3-*d*]*pyrimidine* (**5a**). Compound **5a** was prepared using the above-mentioned procedure using compound **3** (40 mg, 0.18 mmol) and 4-chloro-7-(prop-2-yn-1-yl)-7*H*-pyrrolo[2,3-*d*]pyrimidine (38.6 mg, 0.22 mmol) to obtain **5a** as white powder (57.2 mg, 77%, m.p. = 202–204 °C). ^1^H NMR (600 MHz, DMSO) δ 8.64 (1H, s, H2′′), 8.41 (1H, s, H2), 7.82 (1H, s, H5′), 7.61 (1H, d, *J* = 3.6 Hz, H6′′), 7.47 (1H, d, *J* = 3.6 Hz, H6), 6.65 (1H, d, *J* = 3.6 Hz, H5′′), 6.51 (1H, d, *J* = 3.6 Hz, H5), 5.46 (2H, s, CH_2_′′), 4.81 (2H, t, *J* = 5.7 Hz, CH_2_), 4.70 (2H, t, *J* = 5.7 Hz, CH_2_). ^13^C NMR (75 MHz, DMSO) δ 150.7, 150.7, 150.5, 150.4, 150.3, 150.1, 142.6, 131.0, 123.9, 116.8, 116.6, 98.8, 98.8, 49.1, 44.6, 39.4. Anal. calcd. for C_17_H_13_Cl_2_N_9_: C, 49.29; H, 3.16; N, 30.43. Found: C, 49.59; H, 3.02; N, 30.27.

*6-Chloro-9-((1-(2-(4-chloro-7H-pyrrolo[2,3-d]pyrimidin-7-yl)ethyl)-1H-1,2,3-triazol-4-yl)methyl)-9H-purine* (**5b**). Compound **5b** was prepared using the above-mentioned procedure using compound **3** (16 mg, 0.09 mmol) and 6-chloro-9-(prop-2-yn-1-yl)-9*H*-purine (24.0 mg, 0.11 mmol) to obtain **5b** as white powder (30.9 mg, 83%, m.p. = 214–216 °C). ^1^H NMR (300 MHz, DMSO) δ 8.77 (1H, s, H2′′), 8.68 (1H, s, H2), 8.40 (1H, s, H8′′), 7.94 (1H, s, H5′), 7.46 (1H, d, *J* = 3.6 Hz, H6), 6.51 (1H, d, *J* = 3.6 Hz, H5), 5.51 (2H, s, CH_2_′′), 4.85–4.80 (2H, m, *J* = 6.6, 4.4 Hz, CH_2_), 4.73–4.67 (2H, m, *J* = 6.6, 4.4 Hz, CH_2_). ^13^C NMR (75 MHz, DMSO) δ 151.6, 151.5, 150.6, 150.5, 150.0, 149.1, 147.2, 141.7, 131.0, 130.7, 124.1, 116.6, 98.7, 49.1, 44.6, 38.7. Anal. calcd. for C_16_H_12_Cl_2_N_10_: C, 46.28; H, 2.91; N, 33.73. Found: C, 46.27; H, 3.17; N, 33.77.

*5-Bromo-4-chloro-7-((1-(2-(4-chloro-7H-pyrrolo[2,3-d]pyrimidin-7-yl)ethyl)-1H-1,2,3-triazol-4-yl)methyl)-7H-pyrrolo*[2,3-*d*]*pyrimidine* (**5c**). Compound **5c** was prepared using the above-mentioned procedure using compound **3** (15 mg, 0.07 mmol) and 5-bromo-4-chloro-7-(prop-2-yn-1-yl)-7*H*-pyrrolo[2,3-*d*]pyrimidine (22.7 mg, 0.08 mmol) to obtain **5c** as white crystals (26.2 mg, 76%, m.p. = 168–171 °C). ^1^H NMR (600 MHz, DMSO) δ 8.68 (1H, s, H2), 8.41 (1H, s, H2′′), 7.91 (1H, s, H5′), 7.86 (1H, s, H8′′), 7.46 (1H, d, *J* = 3.6 Hz, H6), 6.51 (1H, d, *J* = 3.6 Hz, H5), 5.45 (2H, s, CH_2_′′), 4.82–4.80 (2H, m, *J* = 6.6, 4.7 Hz, CH_2_), 4.72–4.68 (2H, m, *J* = 6.6, 4.8 Hz, CH_2_). ^13^C NMR (151 MHz, DMSO) δ 151.0, 150.6, 150.5, 150.0, 149.6, 142.1, 131.0, 130.8, 124.0, 116.6, 113.9, 98.6, 85.9, 49.0, 44.5, 39.5. Anal. calcd. for C_17_H_12_BrCl_2_N_9_: C, 41.40; H, 2.45; N, 25.56. Found: C, 41.69; H, 2.34; N, 25.72.

*4-Chloro-7-(2-(4-((5-fluoro-1H-indol-1-yl)methyl)-1H-1,2,3-triazol-1-yl)ethyl)-7H-pyrrolo[2,3-d]pyrimidine* (**5d**). Compound **5d** was prepared using the above-mentioned procedure using compound **3** (40 mg, 0.18 mmol) and 5-fluoro-1-(prop-2-yn-1-yl)-1*H*-indole (37.4 mg, 0.22 mmol) to obtain **5d** as white powder (70.2 mg, 98%, m.p. = 169–171 °C). ^1^H NMR (600 MHz, DMSO) δ 8.44 (1H, s, H2), 7.78 (1H, s, H5′), 7.46–7.41 (2H, m, H6, H7′′), 7.33 (1H, d, *J* = 3.1 Hz, H2′′), 7.29 (1H, dd, *J* = 9.9, 2.4 Hz, H4′′), 6.95 (1H, td, *J* = 9.3, 2.5 Hz, H6′′), 6.51 (1H, d, *J* = 3.6 Hz, H5), 6.41 (1H, dd, *J* = 3.2, 0.6 Hz, H3′′), 5.35 (2H, s, CH_2_′′), 4.82–4.79 (2H, m, CH_2_), 4.72–4.68 (2H, m, CH_2_). ^13^C NMR (75 MHz, DMSO) δ 158.7; 155.6 (d, *J_CF_* = 231.6 Hz), 150.7, 150.6, 150.2, 143.4, 132.3, 131.0, 130.3, 128.5; 128.4 (d, *J* = 10.4 Hz), 123.6, 116.6, 111.1; 111.0 (d, *J_CF_* = 9.9 Hz) 109.4; 109.0 (d, *J_CF_* = 26.1 Hz), 105.1; 104.8 (d, *J_CF_* = 23.1 Hz), 101.0, 101.0 (d, *J_CF_* = 4.7 Hz), 98.8, 49.0, 44.6, 40.9. Anal. calcd. for C_19_H_15_ClFN_7_: C, 57.65; H, 3.82; N, 24.77. Found: C, 57.36; H, 4.01; N, 25.02.

*N-((1-(2-(4-Chloro-7H-pyrrolo[2,3-d]pyrimidin-7-yl)ethyl)-1H-1,2,3-triazol-4-yl)methyl)-4-(5-methylbenzo[d]thiazol-2-yl)benzenamine* (**5e**). Compound **5e** was prepared using the above-mentioned procedure using compound **3** (30 mg, 0.14 mmol) and 4-(5-methylbenzo[*d*]thiazol-2-yl)-*N*-(prop-2-ynyl)benzenamine (48.74 mg, 0.17 mmol) to obtain **5e** as yellow powder (27.3 mg, 38%, m.p. = 228–230 °C). ^1^H NMR (300 MHz, DMSO) δ 8.54 (1H, s, H2), 7.83–7.74 (5H, m, H5′; H4′′; H7′′; Ph′′), 7.40 (1H, d, *J* = 3.6 Hz, H6), 7.27 (1H, dd, *J* = 8.3, 1.2 Hz, H6′′), 6.82 (1H, t, *J* = 5.9 Hz, NH′′), 6.68 (2H, d, *J* = 8.8 Hz, Ph′′), 6.52 (1H, d, *J* = 3.6 Hz, H5), 4.87–4.76 (2H, m, CH_2_), 4.77–4.67 (2H, m, CH_2_), 4.31 (2H, d, *J* = 5.9 Hz, CH_2_′′), 2.43 (3H, s, CH_3_′′). ^13^C NMR (151 MHz, DMSO) δ 167.1, 152.0, 151.4, 150.9, 150.7, 150.3, 145.4, 134.1, 133.9, 131.2, 128.7, 127.7, 123.1, 121.6, 121.4, 120.8, 116.8, 114.1, 99.1, 49.7, 44.6, 38.0, 21.0. Anal. calcd. for C_25_H_21_ClN_8_S: C, 59.93; H, 4.22; N, 22.36. Found: C, 59.84; H, 4.15; N, 22.48.

#### 3.2.2. General Procedure for the Synthesis of Compounds (**6a** and **6b**)

The corresponding diethynylbenzene and azido derivative **3** (1.2 eq.) were dissolved in 0.5 mL DMF and *t*-BuOH:H_2_O = 1:1 (2 mL), Cu(0) (0.5 eq.), 1M CuSO4 (1 eq.) and stirred under microwave irradiation for 1.5 h at 80 °C and 300 W. The solvent was evaporated and the residue was purified by column chromatography using CH_2_Cl_2_:CH_3_OH = 100:1 as an initial eluent and CH_2_Cl_2_:CH_3_OH = 10:1, as final eluent.

*1,4-Bis(1-(2-(4-chloro-7H-pyrrolo[2,3-d]pyrimidin-7-yl)ethyl)-1H-1,2,3-triazol-4-yl)benzene* (**6a**). Compound **6a** was prepared in accord with the general procedure using compound **3** (44.1 mg, 0.20 mmol) and 1,4-diethynylbenzene (11.3 mg, 0.09 mmol) to obtain **6a** as white powder ( 20.4 mg, 39%, m.p. > 250 °C). ^1^H NMR (600 MHz, DMSO) δ 8.57 (2H, s, H2, H2′′), 8.44 (2H, s, H5′), 7.78 (4H, s, Ph), 7.58 (2H, d, *J* = 3.6 Hz, H6, H6′′), 6.61 (2H, d, *J* = 3.6 Hz, H5, H5′′), 5.06–4.84 (4H, m, CH_2_, CH_2_′′), 4.87–4.73 (4H, m, CH_2_, CH_2_′′). ^13^C NMR (151 MHz, DMSO) δ 151.0, 150.7, 150.6, 145.9, 131.2, 130.0, 125.5, 122.1, 116.8, 98.8, 49.1, 44.6. Anal. calcd. for C_26_H_20_Cl_2_N_12_: C, 54.65; H, 3.53; N, 29.41. Found: C, 54.65; H, 3.31; N, 29.78.

*1,3-Bis(1-(2-(4-chloro-7H-pyrrolo[2,3-d]pyrimidin-7-yl)ethyl)-1H-1,2,3-triazol-4-yl)benzene* (**6b**). Compound **6b** was prepared in accord with the general procedure using compound **3** (53.9 mg, 0.24 mmol) and 1,3-diethynylbenzene (13.9 mg, 0.11 mmol) to obtain **6b** as white powder (50.2 mg, 81%, m.p. = 193–196 °C). ^1^H NMR (300 MHz, DMSO) δ 8.59–8.54 (2H, m, H2, H2′′), 8.51–8.47 (2H, m, H5′), 7.80–7.75 (1H, m, Ph), 7.66 (1H, dd, *J* = 7.7, 1.5 Hz, Ph), 7.58–7.53 (2H, m, *J* = 3.6, 1.3 Hz, H6, H6′′), 7.51–7.42 (2H, m, Ph), 6.69–6.56 (2H, m, H5, H5′′), 4.97–4.75 (8H, m, CH_2_). ^13^C NMR (75 MHz, DMSO) δ 150.7, 150.6, 150.2, 146.0, 145.3, 131.3, 131.1, 131.0, 129.4, 128.1, 125.5, 124.6, 122.3, 122.2, 122.0, 116.8, 98.8, 49.2, 44.7, 44.7. Anal. calcd. for C_26_H_20_Cl_2_N_12_: C, 54.65; H, 3.53; N, 29.41. Found: C, 54.58; H, 3.76; N, 29.46.

### 3.3. X-ray Crystal Structure Analysis

Single crystals of **4g**, **5a**, and **5b** suitable for single crystal X-ray structure analysis were obtained at room temperature by partial evaporation from ethanol, methanol and acetone, respectively. Data were collected at room temperature (295 K) on Oxford Diffraction Xcalibur Nova R diffractometer with mirror-monochromatized CuK_α_ radiation (*λ* = 1.54184 Å). Data collection and processing was performed by using the *CrysAlisPro* program [52]. The intensities were corrected for absorption using two different absorption correction methods, multi-scan (for **4g** and **5b**) and analytical (**5a**) [52]. All structures were solved using direct methods with *SIR-2004* [53] and refined by full-matrix least-squares calculations based on *F*^2^ using *SHELXL-2016* [54] integrated in the *WinGX* program package [55]. All hydrogen atoms were included in calculated positions, with *SHELXL-2016* [54] defaults. Geometric restraints and restraints on anisotropic thermal parameters were used in the refinement of slightly disordered butyl chain atoms in **4g**. For structure analysis and molecular and crystal structure drawing preparation were used the *PLATON* [56] and *Mercury* [57] programs. The CCDC 1551719–1551721 contains the supplementary crystallographic data for this paper. These data can be obtained free of charge from The Cambridge Crystallographic Data Centre via www.ccdc.cam.ac.uk/data_request/cif. 

Crystal data for **4g**: 0.315 × 0.163 × 0.072 mm^3^; C_21_H_23_ClN_6_, *M*_r_ = 394.90, triclinic, space group *P* −1 (No. 2); *a* = 4.6875(2) Å, *b* = 12.7403(6) Å, *c* = 17.7949(10) Å, *α* = 109.141(4)°, *β* = 92.486(4)°, *γ* = 91.871(4)°, *V* = 1001.78(9) Å^3^; *Z* = 2; *ρ* = 1.309 g cm^−3^, *μ*(CuK*_α_*) = 1.833 mm^−1^; *θ*_max_ = 70.000°, 7842 reflections measured, 3785 unique reflections and 3298 with *I* ≥ 2*σ*(*I*), *R*_int_ = 0.0212; Final *R* indices [(*I* > 2*σ*(*I*)]: *R* = 0.0621, *wR* = 0.1785, [all data]: *R* = 0.0681, *wR* = 0.1876, *S* = 1.072 for 254 parameters and 11 restraints, largest diff. peak and hole 0.809/−0.528 *e* Å^−3^.

Crystal data for **5a**: 0.622 × 0.083 × 0.030 mm^3^; C_17_H_13_Cl_2_N_9_, *M*_r_ = 414.26, monoclinic, space group *P* 2_1_/*n* (No. 14); *a* = 5.0071(3) Å, *b* = 19.6228(17) Å, *c* = 18.2988(10) Å, *β* = 94.275(4)°, *V* = 1792.9(2) Å^3^; *Z* = 4; *ρ* = 1.535 g cm^−3^, *μ*(CuK*_α_*) = 3.475 mm^−1^; *θ*_max_ = 69.985°, 6929 reflections measured, 3114 unique reflections and 2116 with *I* ≥ 2*σ*(*I*), *R*_int_ = 0.0637; Final *R* indices [(*I* > 2*σ*(*I*)]: *R* = 0.0610, *wR* = 0.0896, [all data]: *R* = 0.1564, *wR* = 0.1837, *S* = 1.003 for 253 parameters and 0 restraints, largest diff. peak and hole 0.266/−0.269 *e* Å^−3^. 

Crystal data for **5b**: 0.386 × 0.301 × 0.196 mm^3^; C_16_H_12_Cl_2_N_10_, *M*_r_ = 415.26, triclinic, space group *P* −1 (No. 2); *a* = 7.2549(3) Å, *b* = 8.6808(3) Å, *c* = 14.7203(6) Å, *α* = 89.171(3)°, *β* = 78.931(4)°, *γ* = 73.387(3)°, *V* = 871.03(6) Å^3^; *Z* = 2; *ρ* = 1.583 g cm^−3^, *μ*(CuK*_α_*) = 3.595 mm^−1^; *θ*_max_ = 69.988°, 6701 reflections measured, 3171 unique reflections and 3022 with *I* ≥ 2*σ*(*I*), *R*_int_ = 0.0266; Final *R* indices [(*I* > 2*σ*(*I*)]: *R* = 0.0373, *wR* = 0.1021, [all data]: *R* = 0.0388, *wR* = 0.1040, *S* = 1.028 for 253 parameters and 0 restraints, largest diff. peak and hole 0.202/−0.270 *e* Å^−3^. 

### 3.4. In Silico

Values of log*P*, *n*-octanol/water partition coefficients, for novel compounds were calculated by ChemAxon algorithm available within MarvinView Ver. 6.2.2. For predictions of plausible biological targets and pharmacological activities web-service PASS (Available online: http://www.pharmaexpert.ru/passonline/index.php; based on the identification of substructure features typical for active compounds was used [47].

### 3.5. Cell Culturing

Human cell lines A549 (lung carcinoma), HeLa (cervical carcinoma), SW620 (colorectal adenocarcinoma, metastatic) and CFPAC-1 (pancreatic cancer, derived from metastatic: liver) as well as on normal human foreskin fibroblasts (HFF) were obtained from the American Type Culture Collection (ATCC, Manassas, VA, USA). Cells were cultured in humidified atmosphere at 37 °C with 5% CO_2_. As growth medium, Dulbecco’s modified Eagle medium (DMEM, Lonza Group, Basel, Switzerland) was used with the addition of fetal bovine serum (Lonza Group, Basel, Switzerland) (10%), l-glutamine (Lonza Group, Basel, Switzerland) (2 mM) and antibiotics: streptomycin (Lonza Group, Basel, Switzerland) (100 µg/mL) and penicillin (Lonza Group, Basel, Switzerland) (100 U/mL).

### 3.6. Proliferation Assay

Cells were seeded onto 96 well microtiter plates at a seeding density of 3000 cells/well for carcinoma cell lines, and 5000 cells/well for normal human fibroblasts. The next day, cells were treated with test agents in five different concentrations (0.01 to 100 µM) and further incubated for 72 h. Dimethyl sulfoxide (DMSO) (solvent), was tested for potential cytotoxic effect but it did not exceed 0.1%. Following 72 h incubation, the MTT assay was performed and measured absorbances were transformed into percentage of cell growth as described previously [58]. Results were obtained from three independent experiments. IC_50_ values were calculated using linear regression analysis and results were statistically analyzed by ANOVA, Tukey *post-hoc* test (*p* < 0.05).

### 3.7. Western Blot Analysis

Cells were seeded in 6-well plates in the concentration depending on tested cell line varying from 1 × 10^5^ to 2 × 10^5^ cells/well. Cells were treated for 48 h with 2 × IC_50_ concentrations of selected compounds. Following treatment, cells were lysed with RIPA (radioimmunoprecipitation assay) buffer supplemented with protease and phosphatase inhibitors (Roche, Basel, Switzerland). Total proteins (50 µg) were resolved on 10% or 12% polyacrilamide gels, deppending on protein size, and transferred onto PVDF (polyvinylidene fluoride) membranes (Bio-Rad, Hercules, CA, USA) that were blocked for 1h with either 4% BSA (bovine serum albumin, Sigma Aldrich, St. Louis, MO, USA) or 5% non-fat milk (Bio-Rad, Hercules, CA, USA) prepared in TBST (Tris-buffered saline, 0.1% Tween 20). Membranes were probed with primary antibodies against CDK9/cyclin T1 (Cell Signaling Technology, Danvers, MA, USA), p-c-Raf (Abcam, Cambridge, UK) and p-p38 MAPK (p-38 MAPK (Thr180/Tyr182) (Cell Signaling Technology) at 4 °C overnight. The next day, membranes were washed in TBST and probed with horseradish peroxidase-conjugated secondary antibodies goat anti-mouse (Santa Cruz Biotechnology, Dallas, TX, USA) or goat anti-rabbit (Santa Cruz Biotechnology, Dallas, TX, USA). Protein bands were visualised using chemiluminesncence supstrate and ImageQuant LAS 500 (GE Healthcare, Little Chalfont, UK). Following visualisation, protein band density was analyzed by Quantity One 1-D Analysis Software (Bio-Rad, Hercules, CA, USA).

### 3.8. Apoptosis Detection

Cells were seeded into 8 well chambers (Lab-tek II Chmaber Slides, Thermo Fisher Scientific, Waltham, MA, USA) in concentration of 2 × 10^4^ cells per well and treated with 2 × IC_50_ concentrations of selected compounds for 48 h. Staining of the cells was performed by Annexin-V-FITC Staining kit (Santa Cruz Biotechnology, Dallas, TX, USA) according to the manufacturer’s instructions. Cells were visualized by fluorescent microscope (Olympus, Shinjuku, Tokyo, Japan) at magnification of 40×.

## 4. Conclusions

A series of mono-pyrrolo[2,3-*d*]pyrimidines comprising alkyl (**4a**, **4b**), varied substituted aryl (**4c**–**4h**) and halogenphenylsulfonamide (**4i**–**4k**) attached to 1,2,3-triazole at C-4, unsymmetrical (**5a**–**5e**) and symmetrical bis-purine isosteres (**6a**, **6b**) were designed and synthesized by 1,3-dipolar Huisgen cycloaddition reaction under microwave irradiation using copper(II) sulfate and metallic copper, as a catalyst. The stereostructures of mono- **4g**, and bis-pseudopurines **5a** and **5b** were unambiguously confirmed by crystallographic analyses.

Mono-pyrrolo[2,3-*d*]pyrimidine **4g** and bis-purine isosteres **5e** exhibited selective anti-proliferative effects on cervical carcinoma cells (HeLa), while **6b** showed strong and selective cytostatic activity (IC_50_ = 0.95 µM) on pancreatic cancer cells (CFPAC-1). Growth-inhibitory effects of symmetrical bis-purine isostere **6b** against CFPAC-1 could be ascribed to induction of late apoptosis and necrosis. At the molecular level, this compound proved to be a potent inhibitor of CDK9/cyclin T1 and to suppress proliferative signaling transduced by p38 MAPK and c-Raf. Overall, new chemical entity based on symmetrical bis-pyrrolo[2,3-*d*]pyrimidines connected through di(ethylene-1,2,3-triazolyl)phenyl spacer was identified as a novel CDK9/cyclin T1 inhibitor for potential treatment of pancreatic cancer. 

## Figures and Tables

**Table 1:** In vitro growth inhibitory effects of synthesized compounds on selected tumor cell lines.

Compd	R	IC_50_^a^ (µM)				Clog*P* ^b^
		A549	CFPAC-1	HeLa	SW620	
**4a**	–(CH_2_)_7_CH_3_	>100	>100	>100	77.8 ± 4.25	4.9
**4b**	–(CH_2_)_3_Cl	>100	86.0 ± 2.13	85.5 ± 5.61	75.9 ± 3.41	2.6
**4c**	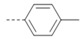	>100	>100	75.5 ± 3.94	99.1 ± 8.36	4.0
**4d**	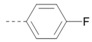	>100	>100	>100	80.5 ± 5.84	3.6
**4e**	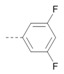	>100	>100	98.5 ± 0.54	>100	3.7
**4f**	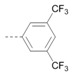	>100	>100	77.2 ± 18.04	>100	5.2
**4g**	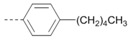	>100	>100	9.5 ± 1.76	16.8 ± 1.97	5.7
**4h**	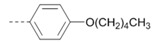	>100	8.1 ± 0.84	7.4 ± 0.19	6.9 ± 1.79	5.1
**4i**	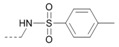	>100	>100	>100	>100	2.6
**4j**	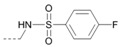	>100	60.8 ± 4.70	64.0 ± 1.70	86.0 ± 2.03	2.2
**4k**	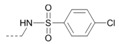	86.2 ± 3.75	41.6 ± 3.24	25.6 ± 3.03	65.1 ± 7.34	2.7
**5a**	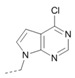	82.9 ± 2.35	77.5 ± 0.17	>100	>100	2.8
**5b**	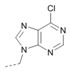	>100	84.2 ± 5.43	84.1 ± 0.02	>100	2.0
**5c**	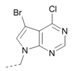	38.4 ± 1.04	31.4 ± 8.45	15.9 ± 2.09	28.4 ± 3.04	3.6
**5d**	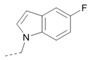	75.6 ± 7.29	60.6 ± 5.52	53.2 ± 5.68	75.8 ± 1.81	3.7
**5e**	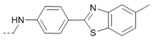	>100	9.8 ± 0.20	5.3 ± 2.69	36.5 ± 1.43	5.4
**6a**	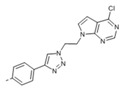	9.4 ± 1.16	3.6 ± 2.02	7.0 ± 0.64	40.8 ± 3.83	4.9
**6b** ^c^	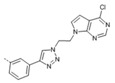	4.2 ± 1.39	0.95 ± 0.28	2.3 ± 0.99	6.8 ± 0.69	4.9
Roscovitine ^c^		24.7 ± 1.15	25.3 ± 2.63	27.2 ± 1.79	28.0 ± 1.83	-

^a^ 50% inhibitory concentration or compound (Compd) concentration required inhibiting tumor cell proliferation by 50%; ^b^ Values of *n*-octanol/water partition coefficients log*P* (Clog*P*) were calculated using ChemAxon algorithm (MarvinView Ver. 6.2.2., Budapest, Hungary). ^c^ IC_50_ = 0.11 ± 0.37 µM for **6b** and IC_50_ = 25.7 ± 0.45 µM for roscovitine on normal foreskin fibroblasts (HFF).

**Table 2:** Annexin V assay for apoptosis detection in CFPAC-1 cells ^a^.

CFPAC-1	Untreated Cells (%)	6b (%)
secondary necrotic cells	10.44	20.72
late apoptotic/primary necrotic cells	5.78	18.02
viable cells	78.04	58.56
early apoptotic cells	5.78	2.70

^a^ The percentages of viable cells (PI^−^/Ann V^−^), early apoptotic cells (PI^−^/Ann V^+^), late apoptotic/primary necrotic cells (PI^+^/Ann V^+^) and secondary necrotic cells (PI^+^) after 48 h treatment with compound **6b** at 2 × IC_50_ value are shown. PI: propidium iodide; Ann V: Annexin V-FITC.

## Supplementary Materials

Supplementary materials can be found at www.mdpi.com/1422-0067/18/11/2292/s1.
