# Supplementary material for: Synthesis and Anti-Proliferative Effects of Mono- and Bis-Purinomimetics Targeting Kinases
Source: Int J Mol Sci. 2017 Nov 1;18(11):2292. doi: 10.3390/ijms18112292 (PMC5713262; doi:10.3390/ijms18112292)

Supporting information for

Synthesis and anti-proliferative effects of mono- and bis-purinomimetics targeting kinases

Andrea Bistrović [1], Anja Harej [2], Petra Grbčić [2], Mirela Sedić [2,*], Sandra Kraljević Pavelić [2], Mario Cetina [3], Silvana Raić-Malić [1,*]

[1] University of Zagreb, Faculty of Chemical Engineering and Technology, Department of Organic Chemistry, Marulićev trg 20, HR-10000 Zagreb, Croatia; E-mail: abistrov@fkit.hr

[2] University of Rijeka, Department of Biotechnology, Center for High-Throughput Technologies, Ulica Radmile Matejčić 2, HR-51000 Rijeka, Croatia; E-mail: aharej@biotech.uniri.hr (A.H.); petra.grbcic@gmail.com (P.G.), sandrakp@biotech.uniri.hr (S.K.P.)

[3] University of Zagreb, Department of Applied Chemistry, Faculty of Textile Technology, Prilaz baruna Filipovića 28a, HR-10000 Zagreb, Croatia; E-mail: mcetina@ttf.hr

* Correspondence: E-mail: msedic@biotech.uniri.hr (M.S.); sraic@fkit.hr (S.R.M.)); Tel.: +85-51-584-574 (M.S.); +385-1-4597-213 (S.R.M.)

**Contents**	**Pages**
1. X-ray crystal structure analyses	S1–S4
2. *In silico* analysis of biological targets by PASS for compd. **4g**, **5b** and **6b**	S5–S7
3. NMR spectra of novel compounds	S8 –S27

1. X-ray crystal structure analyses

Table S1. Selected dihedral and torsion angles (°) in **4g**, **5a** and **5b**

Dihedral / Torsion Angle	**4g**	**5a**	**5b**
Ring 1[a] / Ring 2[b]	14.24(11)	44.21(18)	40.19(8)
Ring 1[a] / Ring 3[c]	3.93(13)	64.50(18)	69.16(8)
C6–N7–C8–C9	–87.0(3)	84.9(5)	–102.17(19)

[a]triazole ring N10–N12/C13/C14.
[b]7-deazapurine ring N1/C2/N3/C4/C4A/C5/C6/N7/C7A.
[c]phenyl ring C16–C20 in **4g**, 7-deazapurine ring N1′/C2′/N3′/C4′/C4A′/C5′/C6′/N7′/C7A′ in **5a**, and purine ring N1/C2/N3/C4/C5/C6/N7/C8/N9 in **5b**.

Table S2 . Intermolecular hydrogen-bonding geometry for **4g**, **5a** and **5b**

	D–H···A	D–H (Å)	H···A (Å)	D···A (Å)	D–H···A (°)	Symmetry codes
**4g**	C2–H2···N1	0.93	2.66	3.385(4)	135	–x, 1–y, –z
	C6–H6···N11	0.93	2.70	3.495(4)	144	–1–x, –y, –z
	C6–H6···N12	0.93	2.49	3.359(4)	156	–1–x, –y, –z
	C8–H8B···N12	0.97	2.54	3.481(3)	162	–x, –y, –z
	C9–H9A···N3	0.97	2.71	3.653(3)	164	–1–x, 1–y, –z
	C14–H14···Cl4	0.93	2.93	3.816(3)	160	–1–x, 1–y, –z
**5a**	C2–H2···N1'	0.93	2.48	3.391(6)	168	–1+x, y, z
	C14–H14···N1	0.93	2.60	3.490(5)	161	1+x,y,z
	C9–H9A···N3	0.97	2.67	3.514(5)	146	1/2+x, 3/2–y, 1/2+z
	C8–H8A···Cl4′	0.97	2.90	3.823(4)	159	1/2+x, 3/2–y, 1/2+z
**5b**	C2–H2···N3′	0.93	2.72	3.619(2)	163	1–x, 2–y, 1–z
	C8'–H8'···N12	0.93	2.49	3.394(2)	163	1–x, 2–y, 1–z
	C14–H14···N3	0.93	2.65	3.513(2)	154	1–x, 1–y, 2–z
	C15–H15A···Cl6′	0.97	2.94	3.8895(18)	168	x, 1+y, z

Table S3 .π···π interactions geometries for **4g** and **5b**

	Ring 1 / Ring 2	Centroids separation (Å)	Interplanar spacing (Å)	Ring inclination (°)	Centroid-centroid offset (Å)	Symmetry codes
**4g**	*Cg*1···*Cg*2[a]	3.7314(15)	3.5661(10)	3.93(13)	1.236	−1+x, y, z
	*Cg*2···*Cg*1[a]	3.7314(15)	3.5208(11)	3.93(13)	1.099	1+x, y, z
**5b**	*Cg*3···*Cg*3[b]	3.7332(10)	3.3083(7)	0.03(8)	1.730	−x, 1−y, 2−z
	*Cg*3···*Cg*3[b]	3.6683(10)	3.4089(7)	0.03(8)	1.355	1−x, 1−y, [illegible]

[a] *Cg*1 and *Cg*2 are centroids of the N10–N12/C13/C14 and C15–C20 rings.
[b] *Cg*3 is centroid of the N1/C2/N3/C4/C4A/C7A ring.

Figure S1. Capped stick representations of **4g**, showing two-dimensional network formed by hydrogen bonds (a) and parallel arrangement of triazole and phenyl rings (b).

a)

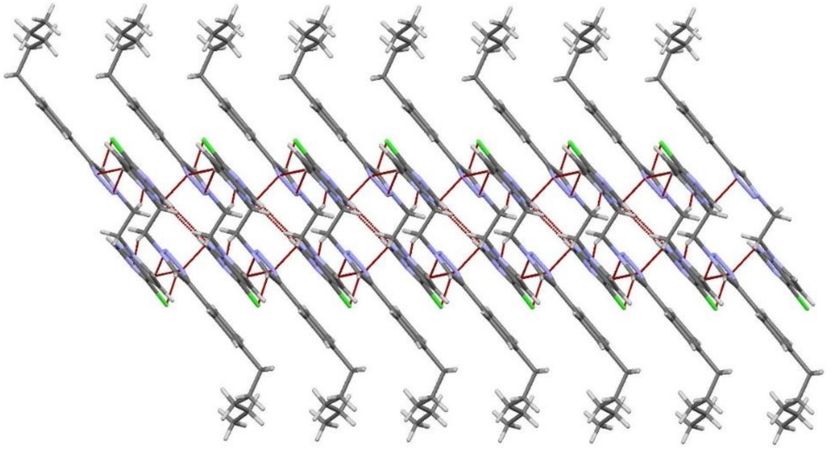

b)

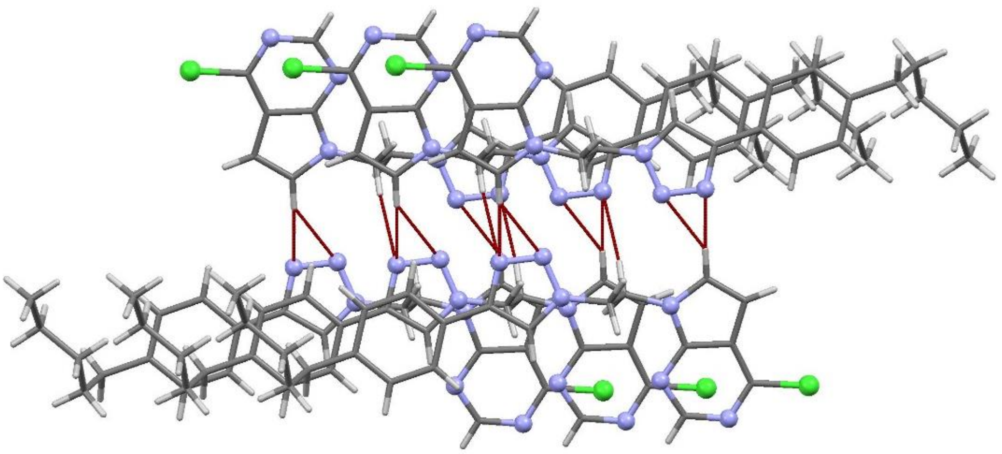

Figure S2. Capped stick representations of **5a**, showing two-dimensional network formed by hydrogen bonds. Nitrogen and chlorine atoms are presented in ball and stick style.

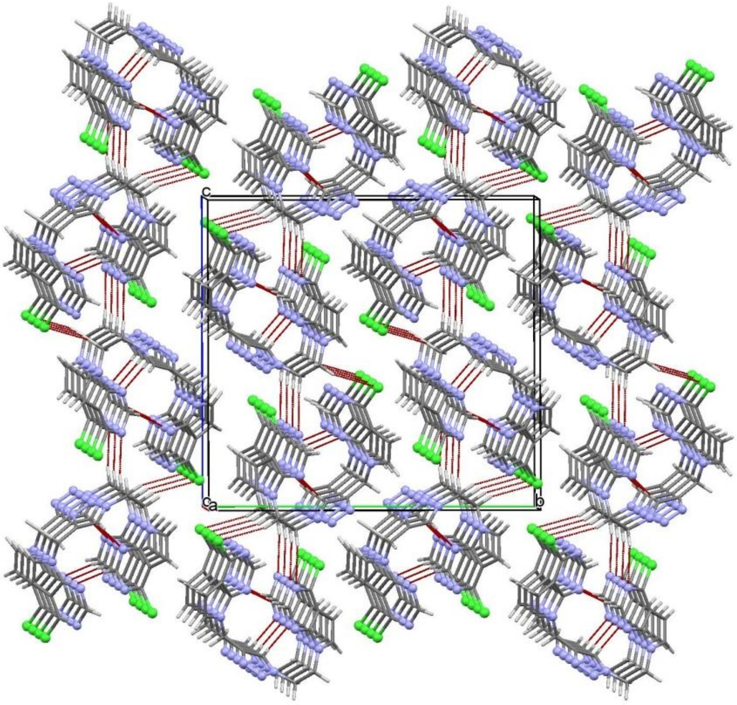

Figure S3. Capped stick representations of **5b**, showing two-dimensional network formed by hydrogen bonds (a) and π···π interactions that link two-dimensional network into three-dimensional (b). Nitrogen and chlorine atoms are presented in ball and stick style.

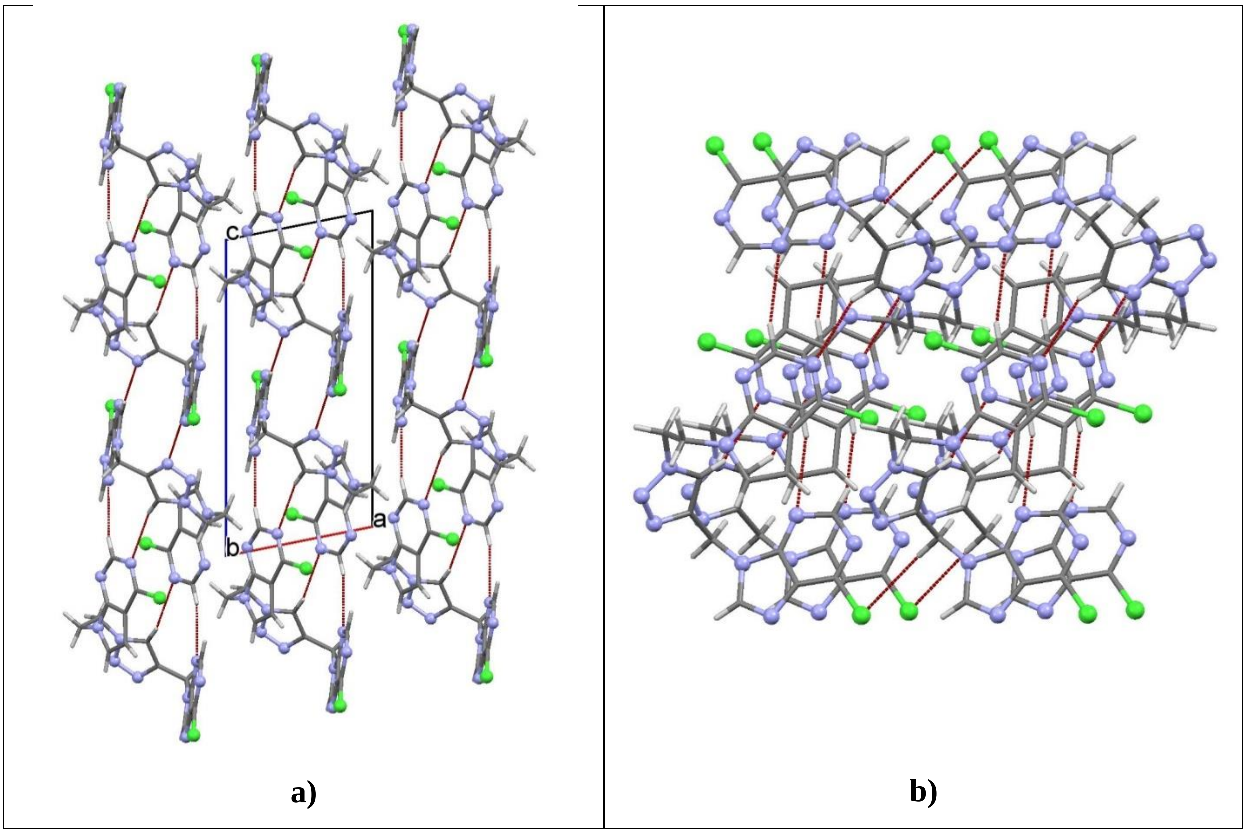

a) **b)**

2. *In silico* analysis of biological targets

Table S4. The prediction of activity spectra for substances (PASS) analysis. Most probable biological targets as predicted by PASS[a] for compound **4g.**

**Biological targets**	**4g**		**Biological targets**	**4g**	
	Pa	Pi		Pa	Pi
CF transmembrane conductance regulator agonist	0.583	0.004	Angiotensin AT1 receptor antagonis	0.062	0.021
Neurodegenerative diseases treatment	0.427	0.055	Angiotensin antagonist	0.062	0.023
**CDK9/cyclin T1 inhibitor**	**0.408**	**0.041**	Angiotensin II receptor antagonist	0.061	0.024
Hedgehog signaling inhibitor	0.327	0.004	mTOR inhibitor	0.045	0.010
Glycine-tRNA ligase inhibitor	0.303	0.049	Protein-tyrosine kinase (PTK, not ETK,WZC) inhibitor	0.104	0.074
17-Beta-hydroxysteroid dehydrogenase 1 inhibitor	0.255	0.004	H+-transporting two sector ATPase inhibitor	0.215	0.187
Macular degeneration treatment	0.240	0.022	Fibroblast growth factor 2 antagonist	0.108	0.080
Nicotinic alpha3beta4 receptor agonist	0.222	0.009	Abl kinase inhibitor	0.100	0.074
Antiarthritic	0.327	0.117	Lipase inhibitor	0.062	0.035
Antiinfertility, female	0.268	0.066	Posttraumatic stress disorder treatment	0.114	0.089
Tumour necrosis factor alpha release inhibitor	0.214	0.044	Vascular endothelial growth factor 1 antagonist	0.079	0.056
Interleukin 1 antagonist	0.192	0.036	CD45 antagonist	0.043	0.021
Interleukin antagonist	0.214	0.070	Antineoplastic (sarcoma)	0.158	0.135
Alzheimer's disease treatment	0.225	0.122	Inosine nucleosidase inhibitor	0.091	0.075
Channel-conductance-controlling ATPase inhibitor	0.197	0.123	Vanilloid 1 agonist	0.231	0.217
5-Hydroxytryptamine 6 agonist	0.086	0.017	T-cell protein-tyrosine phosphatase inhibitor	0.033	0.020
Multidrug resistance associated protein inhibitor	0.089	0.022	Oleamide hydrolase inhibitor	0.038	0.025
Multidrug resistance associated protein 1 inhibitor	0.086	0.022	**p38 MAP inhibitor**	**0.074**	**0.062**
Adenosine deaminase inhibitor	0.083	0.022	Antiinflammatory	0,238	0,227
Antiviral (Rhinovirus)	0.304	0.244	Phosphatidylinositol 3-kinase delta inhibitor	0,051	0,040
Protein-tyrosine phosphatase 2C inhibitor	0.078	0.024	Factor XIa inhibitor	0,062	0,054
**Antineoplastic**	**0.243**	**0.191**	Fibroblast growth factor agonist	0,230	0,224
Adenosine receptor antagonist	0.085	0.034	Alpha 3 adrenoreceptor agonist	0,064	0,060
**MAP kinase kinase inhibitor**	**0.081**	**0.031**	Proto-oncogene tyrosine-protein kinase c-hck inhibitor	0,036	0,032
Imidazoline I1 receptor agonist	0.189	0.141	Hepatocyte growth factor antagonist	0,074	0,070
Nicotinic alpha4beta2 receptor agonist	0.055	0.010	Prenyl-diphosphatase inhibitor	0,138	0,135
Protocollagen prolyl hydroxylase inhibitor	0.068	0.026	Purinergic receptor antagonist	0,059	0,057
Interleukin 1b antagonist	0.092	0.051	Antiinflammatory	0,238	0,227
Angiotensin antagonist	0.062	0.023			

[a]Activity spectrum predicted by PASS is presented by the list of activities with the probabilities "to be active" (Pa) and "to be inactive" (Pi) calculated for each activity. Increased Pa and decreased Pi, the more probable is predicted activity. The list is arranged in descending order of Pa-Pi; therefore, more probable activities are at the top of the list.

Table S5. The prediction of activity spectra for substances (PASS) analysis. Most probable biological targets as predicted by PASS[a] for compound **5e**.

**Biological targets**	**5e** Pa	Pi	**Biological targets**	**5e** Pa	Pi
CF transmembrane conductance regulator agonist	0.546	0.006	Analgesic, non-opioid	0.250	0.140
Neurodegenerative diseases treatment	0.557	0.021	Adenosine regulator	0.216	0.117
Transcription factor inhibitor	0.486	0.014	Epidermal growth factor receptor kinase inhibitor	0.144	0.045
**MAP kinase kinase inhibitor**	**0.428**	**0.003**	Signal transduction pathways inhibitor	0.194	0.097
Proto-oncogene tyrosine-protein kinase Fgr inhibitor	0.420	0.011	Transcription factor STAT inhibitor	0.223	0.126
Protein-serine-threonine kinase inhibitor	0.406	0.011	17-Beta-hydroxysteroid dehydrogenase 1 inhibitor	0.107	0.011
**CDK9/cyclin T1 inhibitor**	**0.414**	**0.039**	Ca2+/calmodulin-dependent kinase II inhibitor	0.121	0.027
Antiinfertility, female	0.391	0.020	Imidazoline I1 receptor agonist	0.204	0.115
Protein kinase inhibitor	0.366	0.031	Proto-oncogene tyrosine-protein kinase Fyn inhibitor	0.171	0.084
Hedgehog signaling inhibitor	0.280	0.005	mTOR inhibitor	0.079	0.005
Autoimmune disorders treatment	0.326	0.085	Protein-tyrosine kinase Lyn inhibitor	0.125	0.052
Antiparkinsonian	0.294	0.067	5 Hydroxytryptamine 6 agonist	0.083	0.019
Transglutaminase 2 inhibitor	0.224	0.005	Nicotinic alpha4beta2 receptor agonist	0.062	0.009
Leukotriene synthesis inhibitor	0.238	0.025	Adenosine receptor antagonist	0.085	0.034
Atherosclerosis treatment	0.293	0.083	Protein-tyrosine kinase (PTK, not ETK, WZC) inhibitor	0.115	0.064
**Antineoplastic**	**0.337**	**0.132**	Abl kinase inhibitor	0.111	0.063
Nicotinic alpha3beta4 receptor agonist	0.209	0.010	Hepatocyte growth factor antagonist	0.085	0.049
Macular degeneration treatment	0.226	0.027	Phosphatidylinositol 3-kinase delta inhibitor	0.060	0.029
Glycine-tRNA ligase inhibitor	0.264	0.074	Multidrug resistance-associated protein inhibitor	0.080	0.055
Lck kinase inhibitor	0.216	0.025	Multidrug resistance associated protein 1 inhibitor	0.076	0.054
Protein-tyrosine kinase p55(blk) inhibitor	0.228	0.059	Antiarthritic	0.219	0.197
Alzheimer's disease treatment	0.257	0.090	Cyclin-dependent kinase 7 inhibitor	0.094	0.072
Ca2+/calmodulin-dependent protein kinase inhibitor	0.032	0.191	Benzodiazepine inverse agonist	0.054	0.034
Src kinase inhibitor	0.185	0.034	Phosphatidylinositol 3-kinase inhibitor	0.057	0.041
Interleukin 2 agonist	0.290	0.149	Proto-oncogene tyrosine-protein kinase c-hck inhibitor	0.041	0.025
Tyrosine kinase inhibitor	0.185	0.061	Phosphatidylinositol kinase inhibitor	0.058	0.042
Rho-associated kinase I inhibitor	0.133	0.014	Antineoplastic (solid tumors)	0.211	0.200
Rheumatoid arthritis treatment	0.221	0.110	**MAP3K8 inhibitor**	**0.071**	**0.066**

[a]Activity spectrum predicted by PASS is presented by the list of activities with the probabilities "to be active" (Pa) and "to be inactive" (Pi) calculated for each activity. Increased Pa and decreased Pi, the more probable is predicted activity. The list is arranged in descending order of Pa-Pi; therefore, more probable activities are at the top of the list.

Table S6. The prediction of activity spectra for substances (PASS) analysis. Most probable biological targets as predicted by PASS[a] for compound **6b**.

Biological targets	6b		Biological targets	6b	
	Pa	Pi		Pa	Pi
CF transmembrane conductance regulator agonist	0.602	0.004	**MAP kinase kinase inhibitor**	**0.131**	**0.012**
Hedgehog signaling inhibitor	0.527	0.004	Hepatocyte growth factor antagonist	0.136	0.017
Neurodegenerative diseases treatment	0.528	0.026	Hepatocyte growth factor antagonist	0.136	0.017
**CDK9/cyclin T1 inhibitor**	**0.444**	**0.028**	Vascular endothelial growth factor 1 antagonist	0.139	0.023
**Antineoplastic**	**0.440**	**0.090**	Polyribonucleotide n ucleotidyltransferase inhibitor	0.206	0.097
17-Beta-hydroxysteroid dehydrogenase 1 inhibitor	0.337	0.004	Nucleoside oxidase (H2O2-forming) inhibitor	0.203	0.094
Antiarthritic	0.390	0.083	Epidermal growth factor receptor kinase inhibitor	0.148	0.043
Glycine-tRNA ligase inhibitor	0.340	0.034	Nicotinic alpha4beta2 receptor agonist	0.105	0.005
Macular degeneration treatment	0.298	0.008	K(Ca) 3.1 channel activator	0.167	0.068
Phthalate 4,5-dioxygenase inhibitor	0.411	0.123	Potassium channel intermediate-conductance Ca-activated activator	0.167	0.068
Nicotinic alpha3beta4 receptor agonist	0.271	0.005	c-Src kinase inhibitor	0.128	0.030
Interleukin antagonist	0.295	0.036	mTOR inhibitor	0.101	0.005
Interleukin 1 antagonist	0.248	0.017	Fibroblast growth factor 3 antagonist	0.126	0.031
Protein kinase inhibitor	0.275	0.047	Src kinase inhibitor	0.138	0.051
Tumour necrosis factor alpha release inhibitor	0.256	0.032	Inosine nucleosidase inhibitor	0.132	0.046
Ligase inhibitor	0.248	0.027	Protocollagen prolyl hydroxylase inhibitor	0.093	0.008
Serum-glucocorticoid regulated kinase 1 inhibitor	0.292	0.081	Posttraumatic stress disorder treatment	0.129	0.045
Imidazoline I1 receptor agonist	0.258	0.063	Protein-tyrosine phosphatase 2C inhibitor	0.096	0.014
Alzheimer's disease treatment	0.271	0.080	Multidrug resistance-associated protein inhibitor	0.094	0.014
Nucleotide metabolism regulator	0.295	0.134	Multidrug resistance-associated protein 1 inhibitor	0.090	0.014
Analgesic, non-opioid	0.277	0.117	Tyrosine kinase inhibitor	0.152	0.076
Protein-tyrosine kinase (PTK, not ETK, WZC) inhibitor	0.185	0.026	Adenosine regulator	0.207	0.133
Abl kinase inhibitor	0.182	0.026	Phosphatidylinositol kinase inhibitor	0.094	0.021
Signal transduction pathways inhibitor	0.229	0.081	Phosphatidylinositol 3-kinase delta inhibitor	0.085	0.012
Antineoplastic (brain cancer)	0.224	0.088	Anxiolytic	0.197	0.124
**p38 MAP kinase inhibitor**	**0.154**	**0.021**	Ribosomal protein S6 kinase, 90-kDa inhibitor	0.131	0.058
Fibroblast growth factor 2 antagonist	0.155	0.028	Hepatocyte growth factor antagonist	0.136	0.017
Interleukin 1b antagonist	0.150	0.024	Vascular endothelial growth factor 1 antagonist	0.139	0.023
Antiprotozoal (Trichomonas)	0.221	0.096	Polyribonucleotide n ucleotidyltransferase inhibitor	0.206	0.097
Growth factor antagonist	0.163	0.041	Nucleoside oxidase (H2O2-forming) inhibitor	0.203	0.094
5 Hydroxytryptamine 6 agonist	0.128	0.008	Epidermal growth factor receptor kinase inhibitor	0.148	0.043

[a]Activity spectrum predicted by PASS is presented by the list of activities with the probabilities "to be active" (Pa) and "to be inactive" (Pi) calculated for each activity. Increased Pa and decreased Pi, the more probable is predicted activity. The list is arranged in descending order of Pa-Pi; therefore, more probable activities are at the top of the list.

3. NMR spectra of novel compounds

Figure S4. a) ^{1}H NMR and b) ^{13}C NMR of compd. **2**

a)

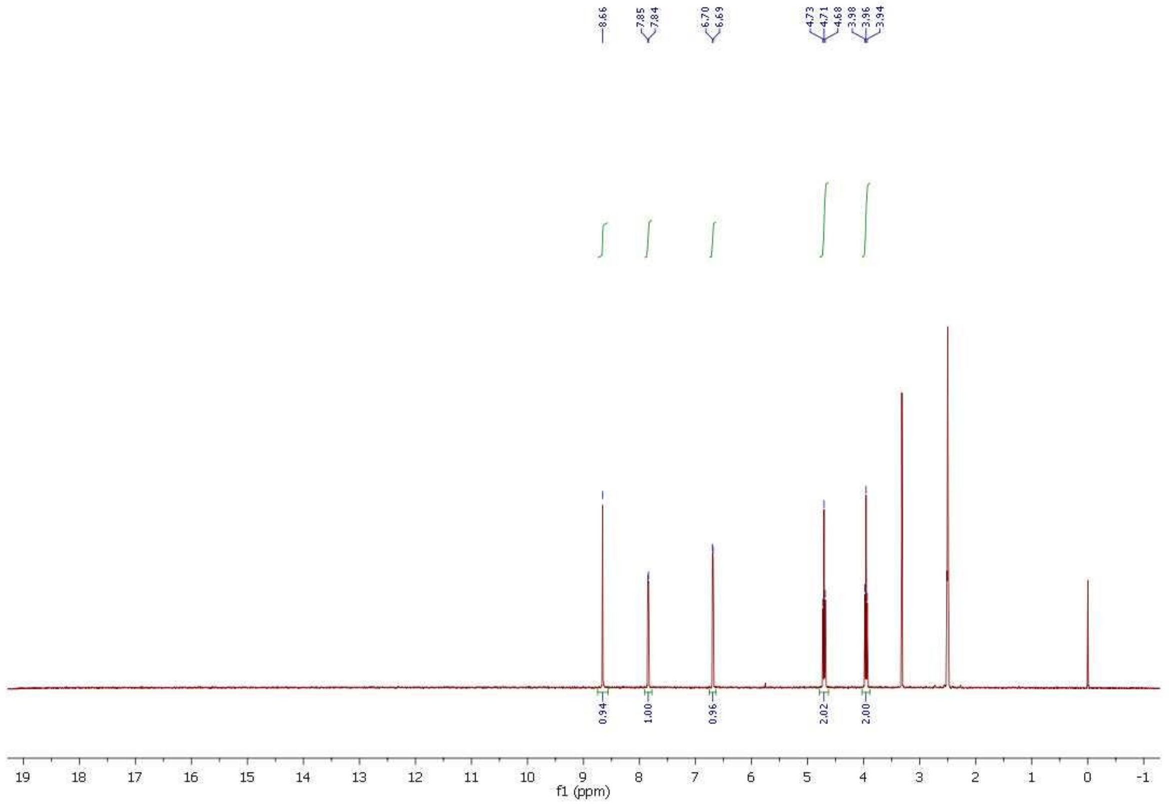

b)

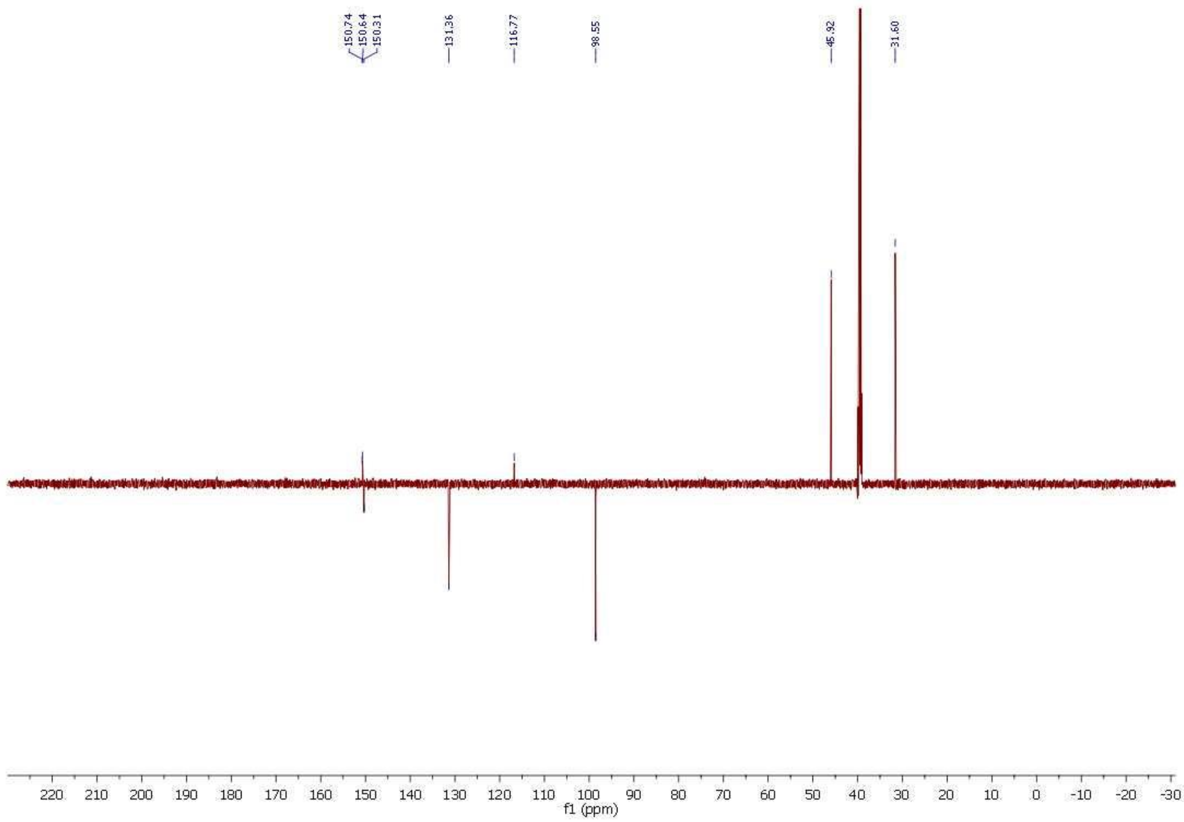

Figure S5. a) ^{1}H NMR and b) ^{13}C NMR of compd. **3**

a)

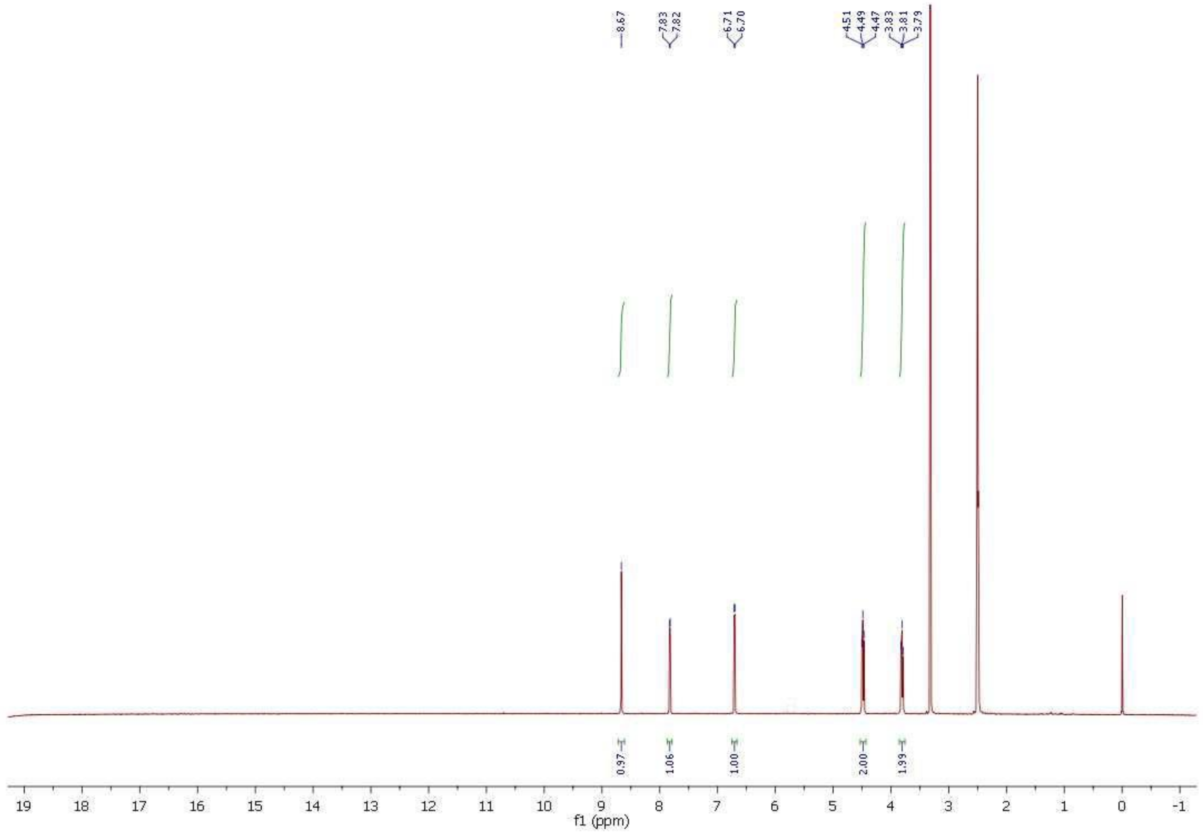

b)

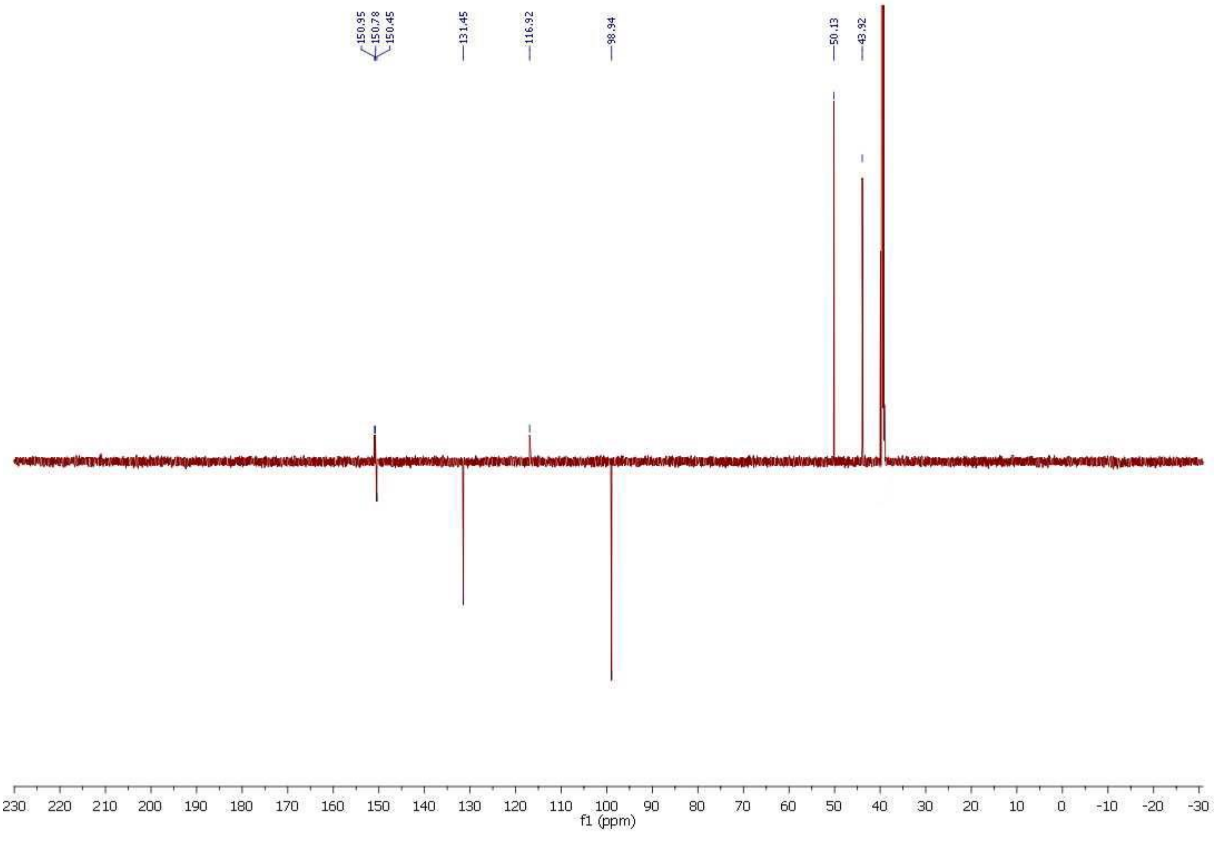

Figure S6. a) ^{1}H NMR and b) ^{13}C NMR of compd. **4a**

a)

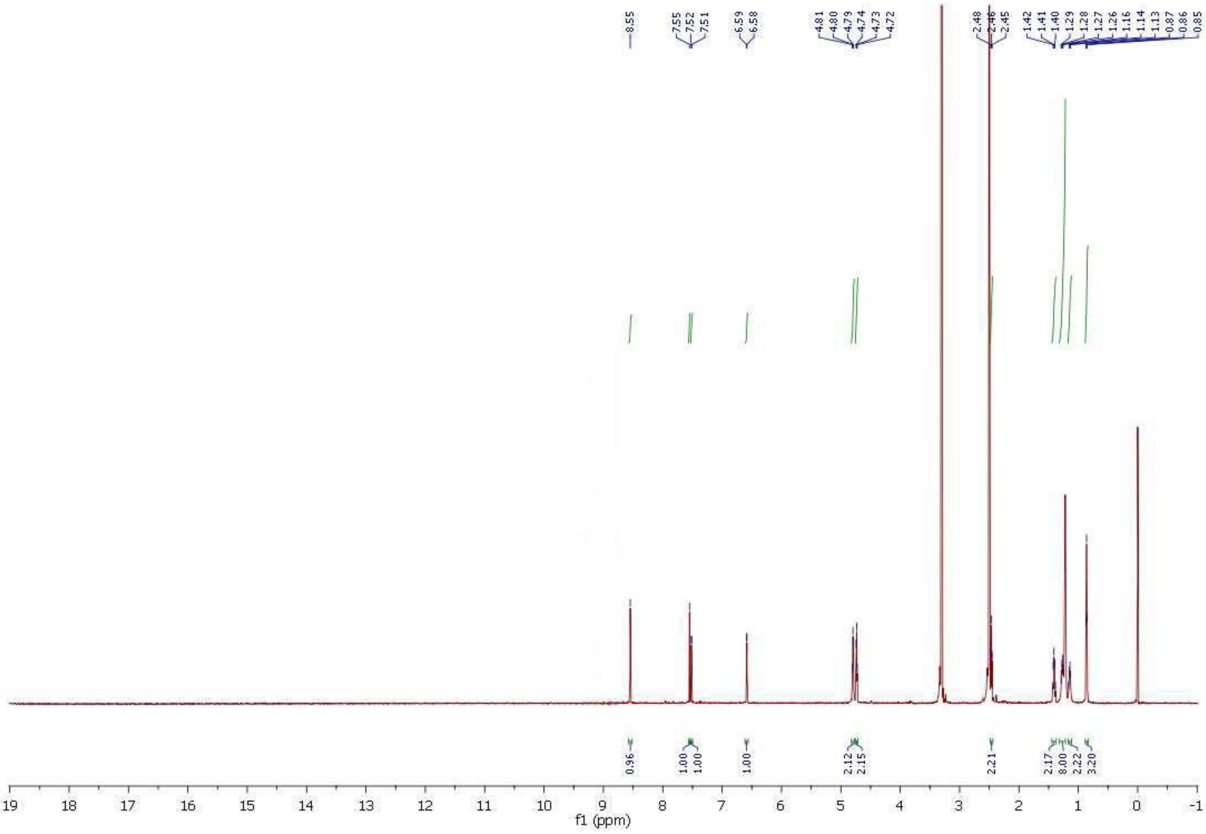

b)

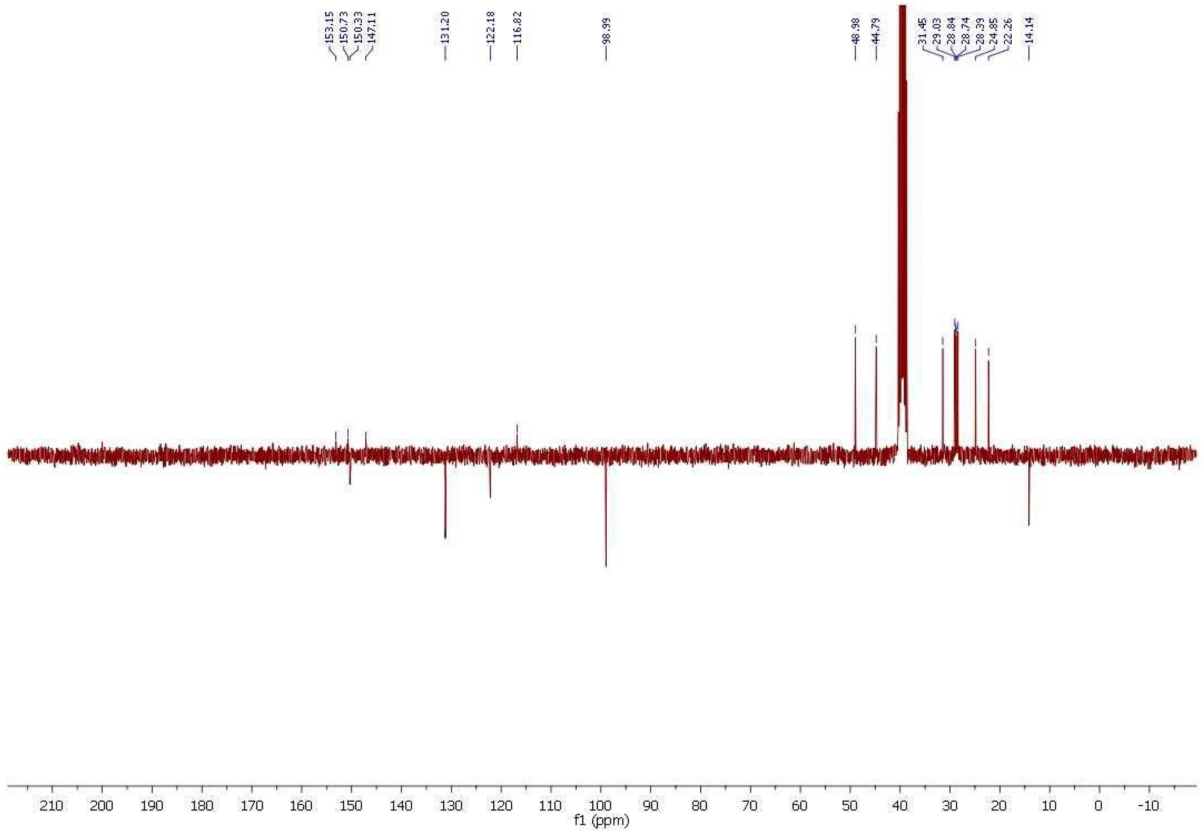

Figure S7. a) ¹H NMR and b) ¹³C NMR of compd. **4b**

a)

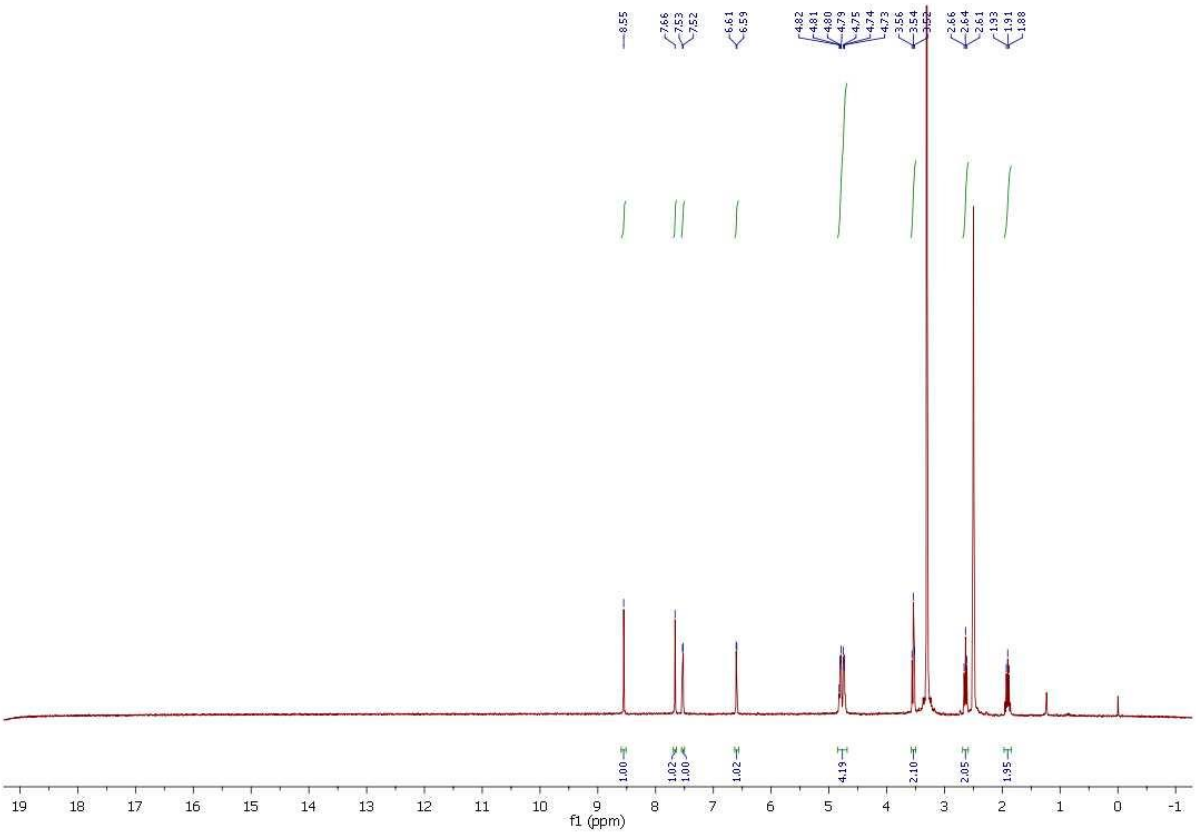

b)

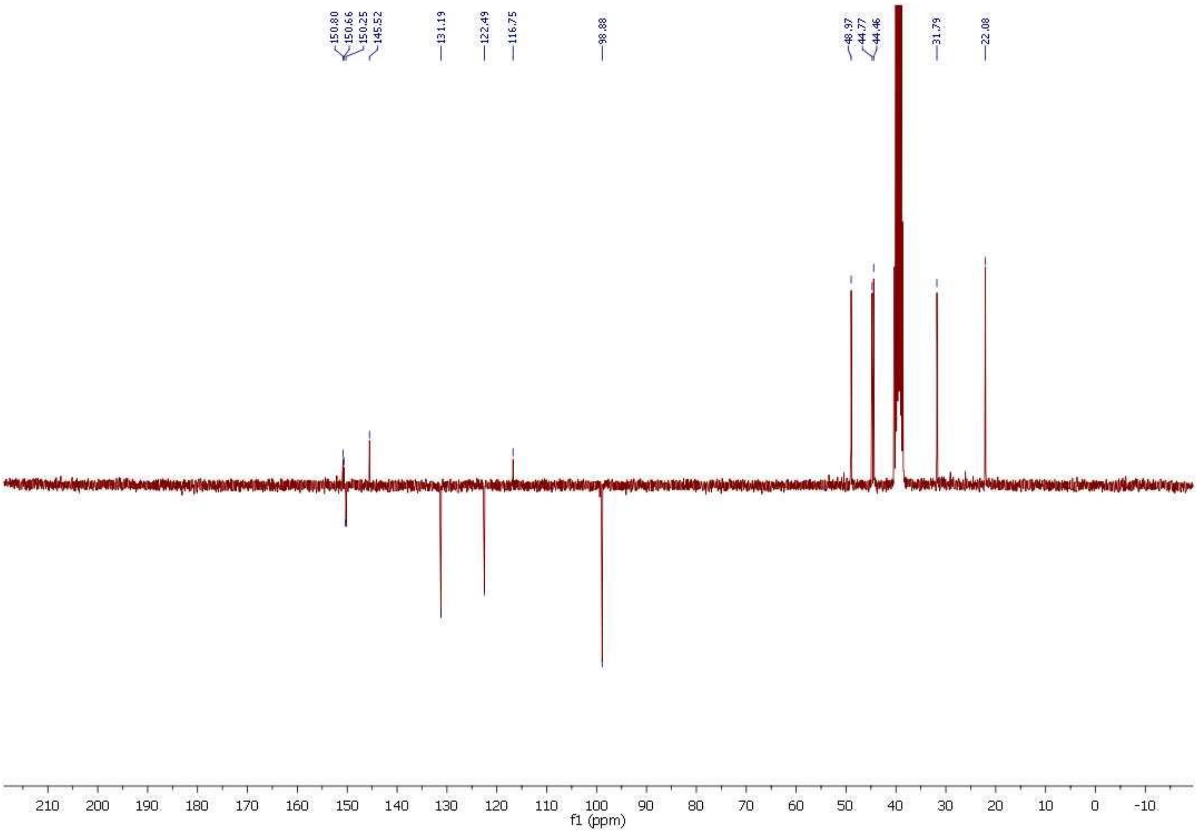

Figure S8. a) ^{1}H NMR and b) ^{13}C NMR of compd. **4c**

a)

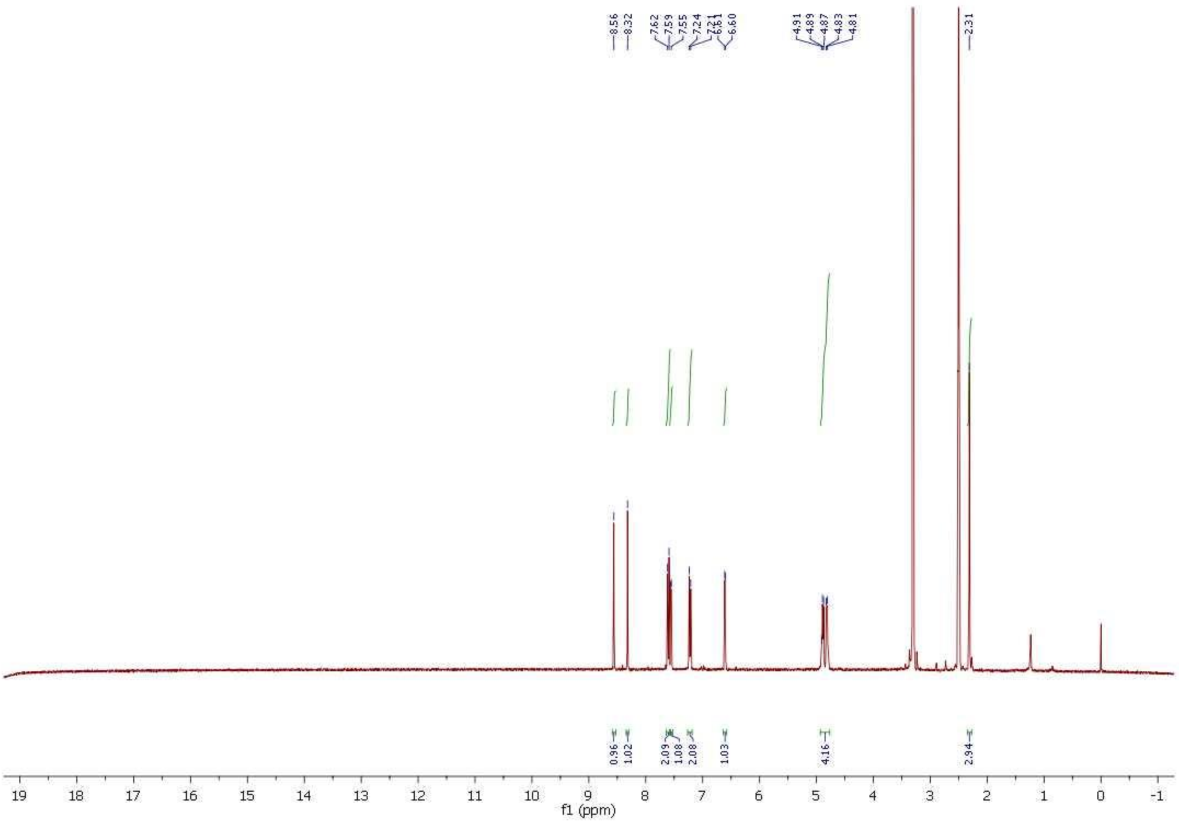

b)

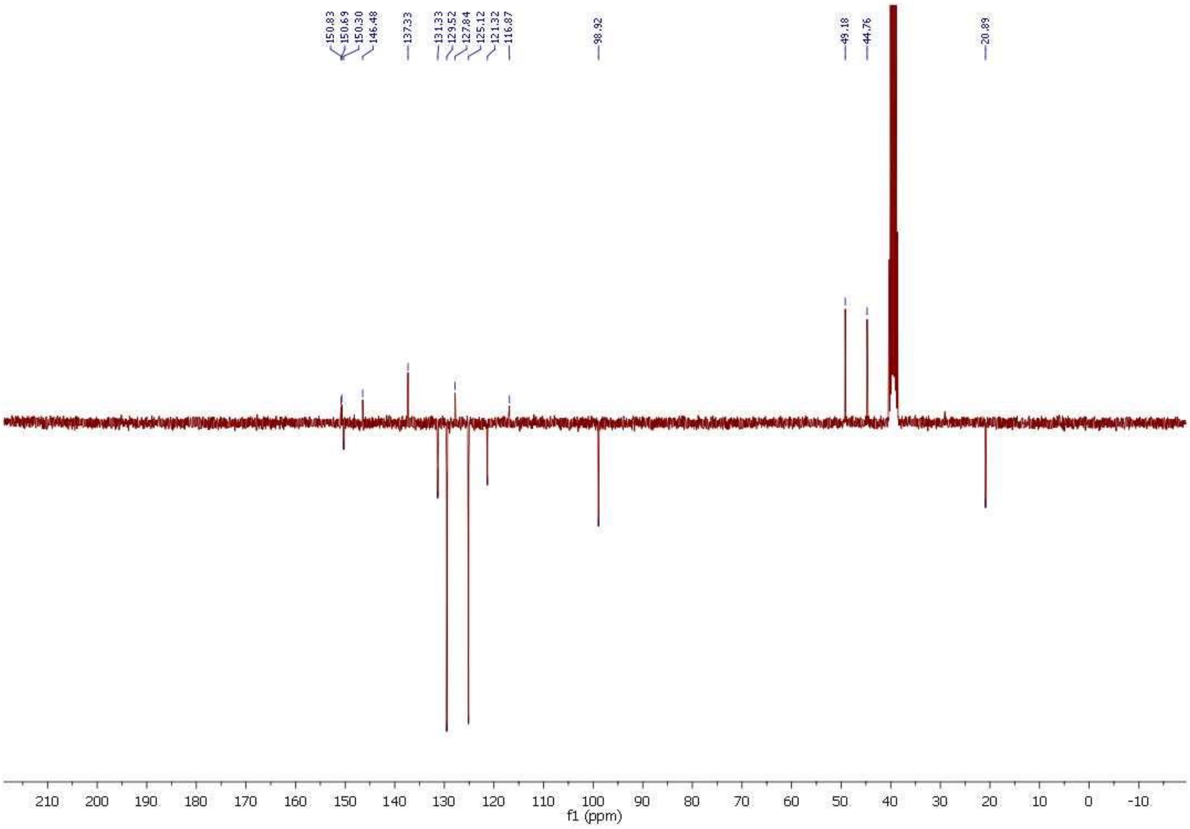

Figure S9. a) ^{1}H NMR and b) ^{13}C NMR of compd. **4d**

a)

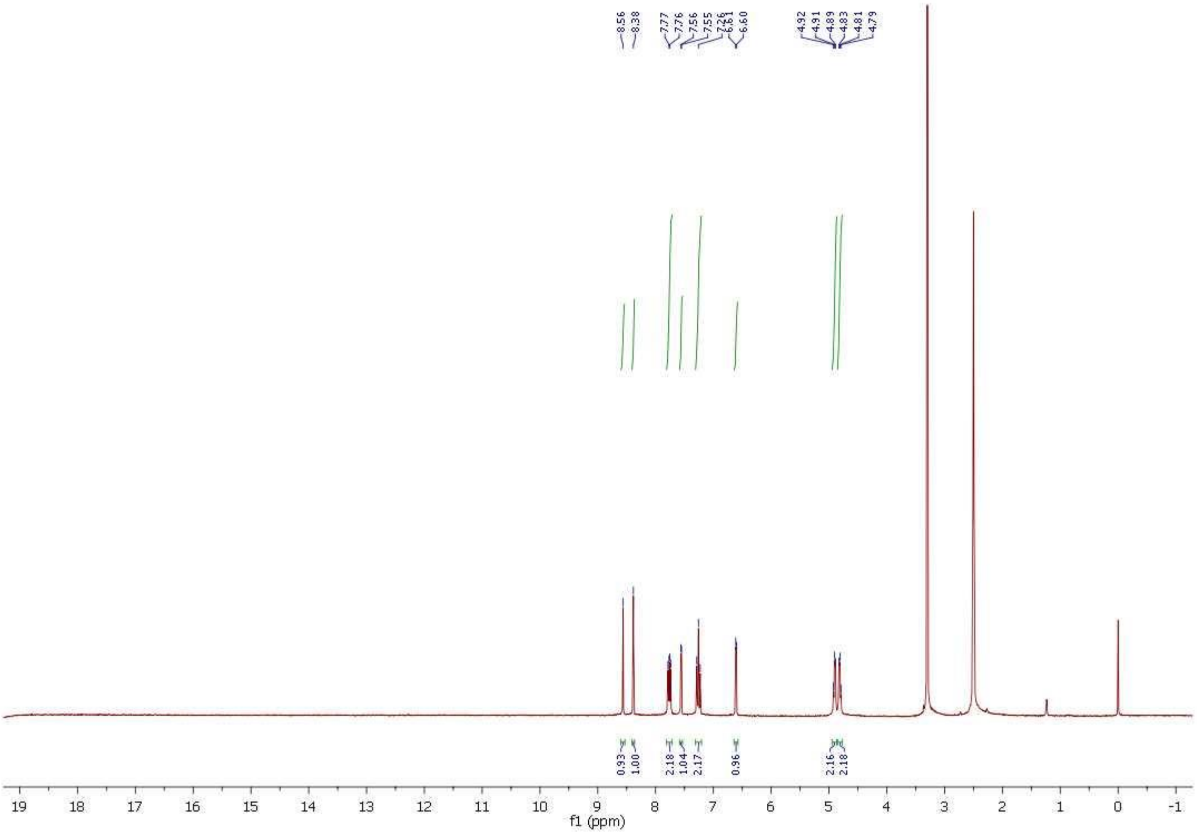

b)

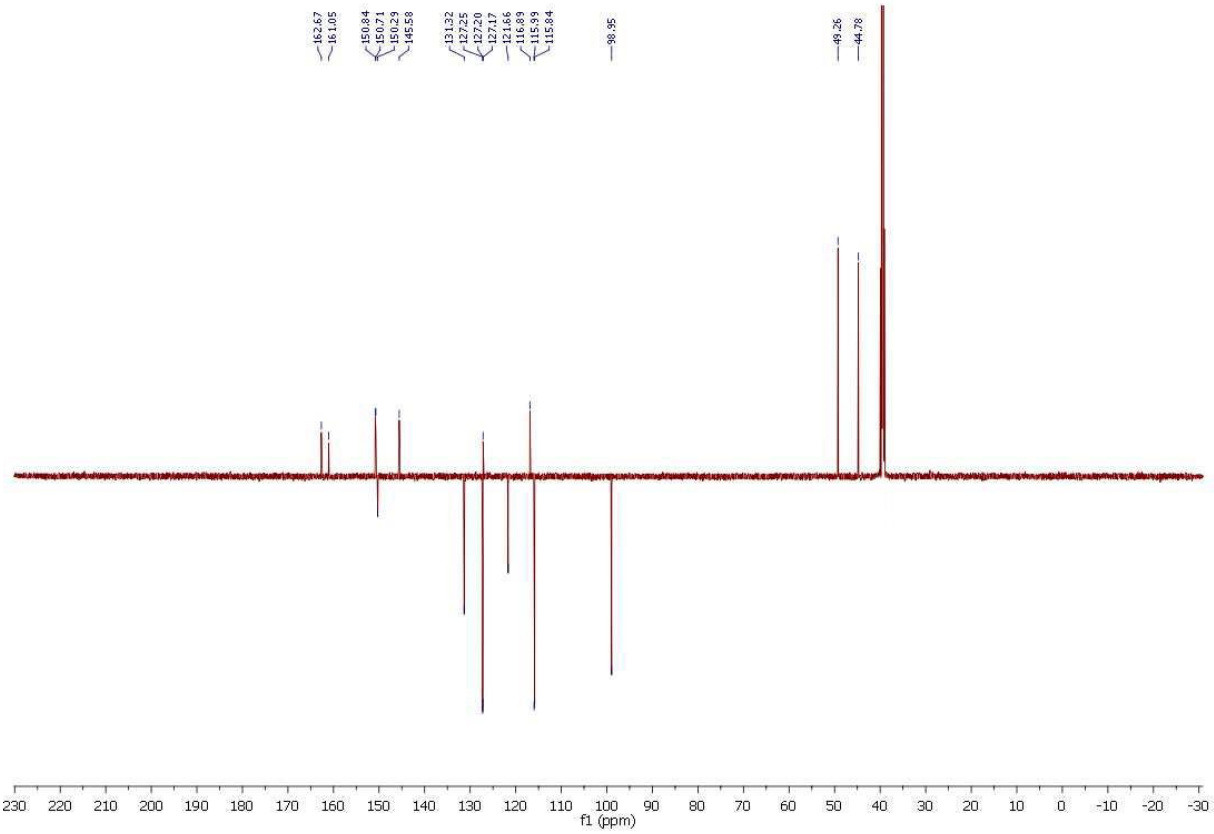

Figure S10 a) ^{1}H NMR and b) ^{13}C NMR of compd. **4e**

a)

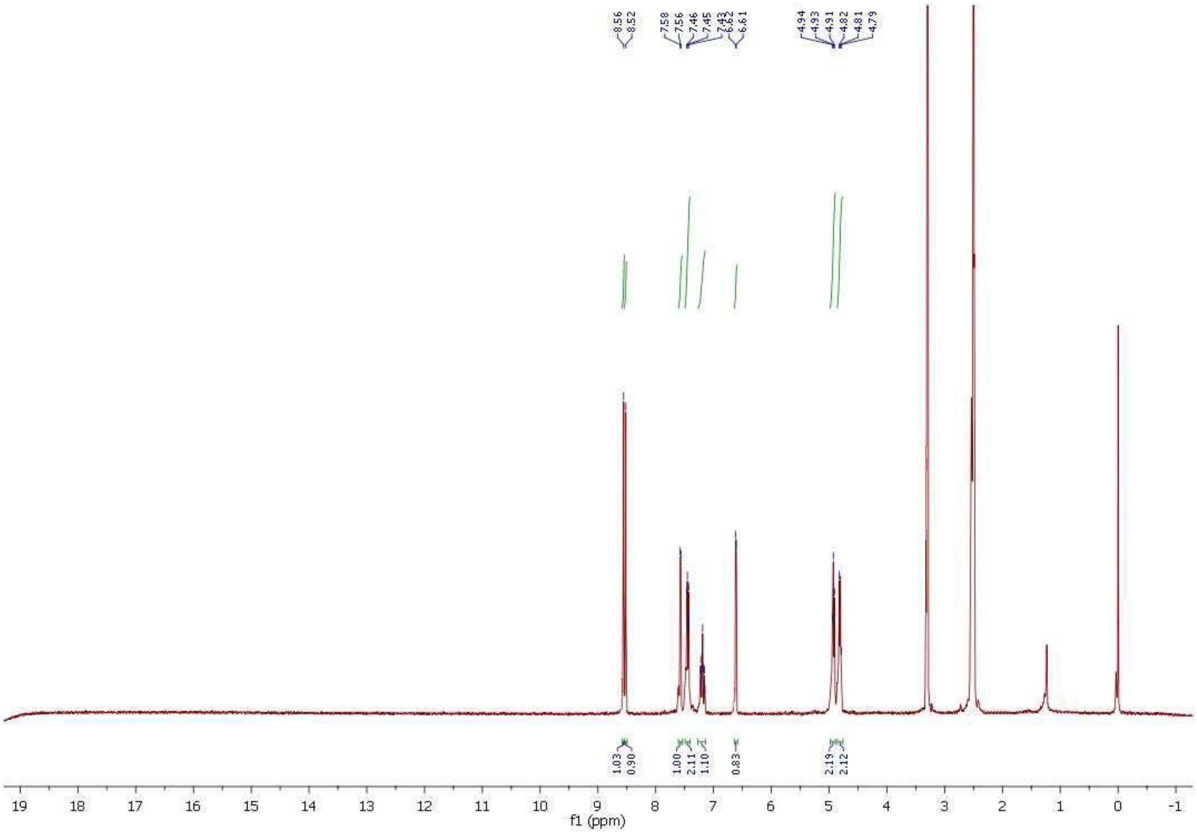

b)

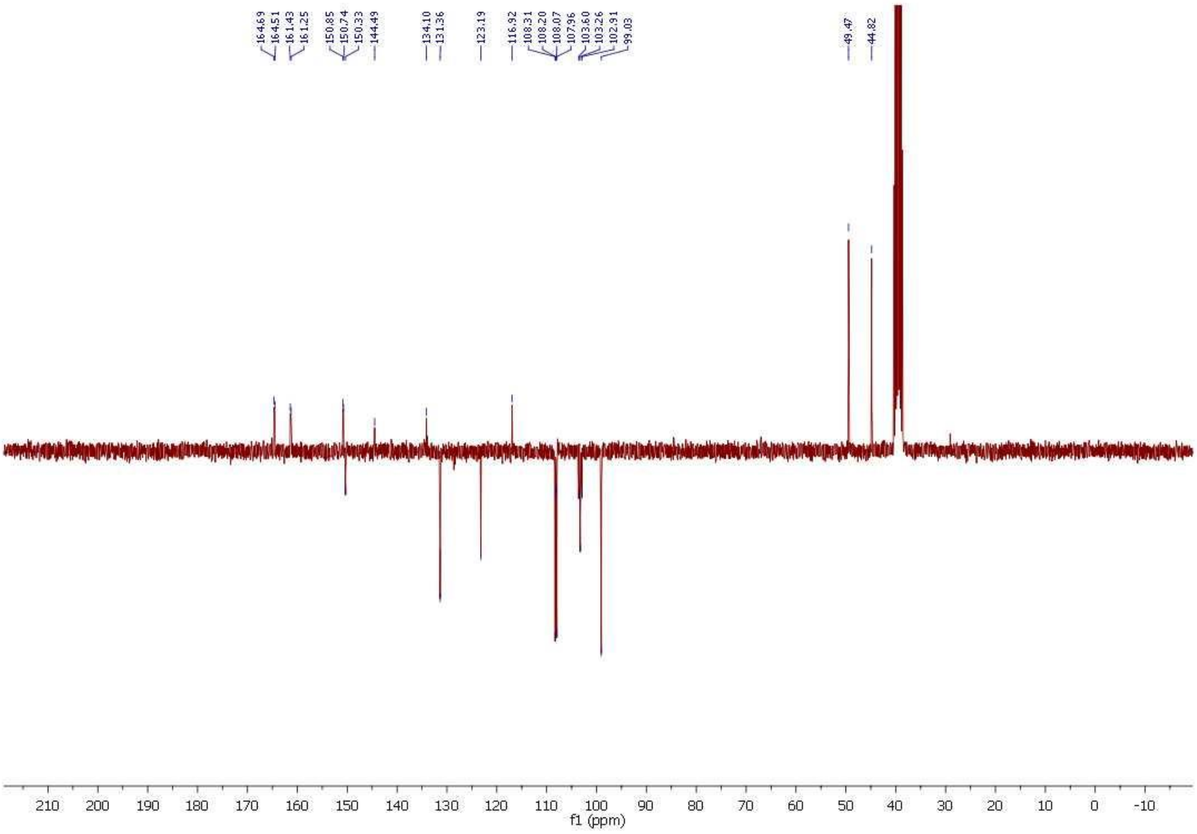

Figure S11. a) ^{1}H NMR and b) ^{13}C NMR of compd. **4f**

a)

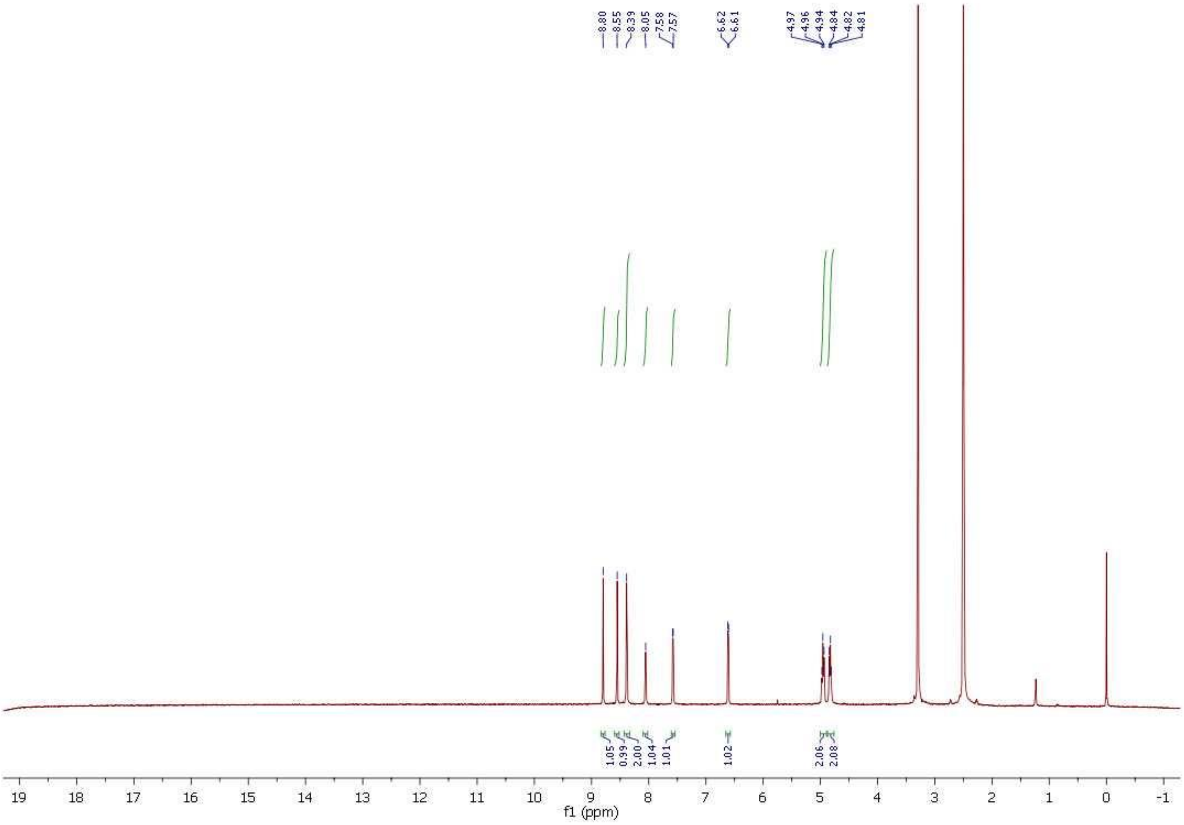

b)

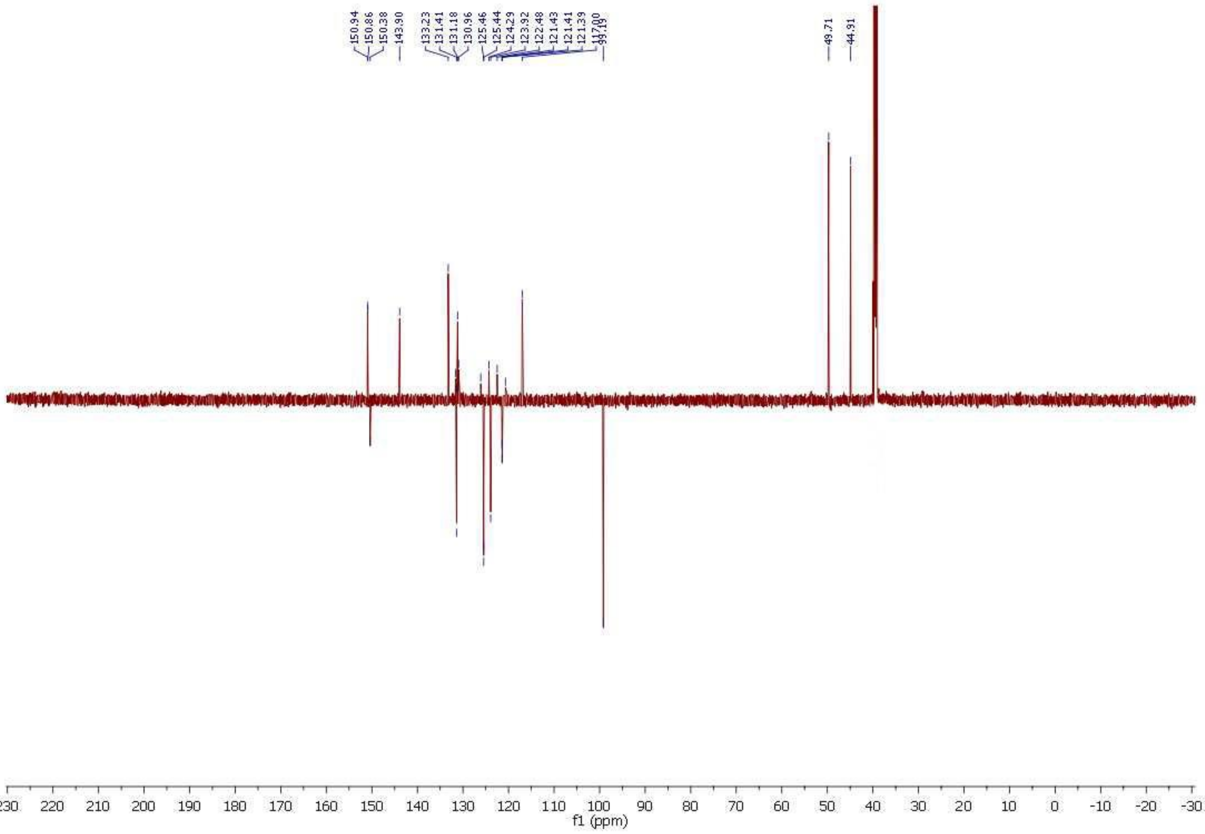

Figure S12. a) ^{1}H NMR and b) ^{13}C NMR of compd. **4g**

a)

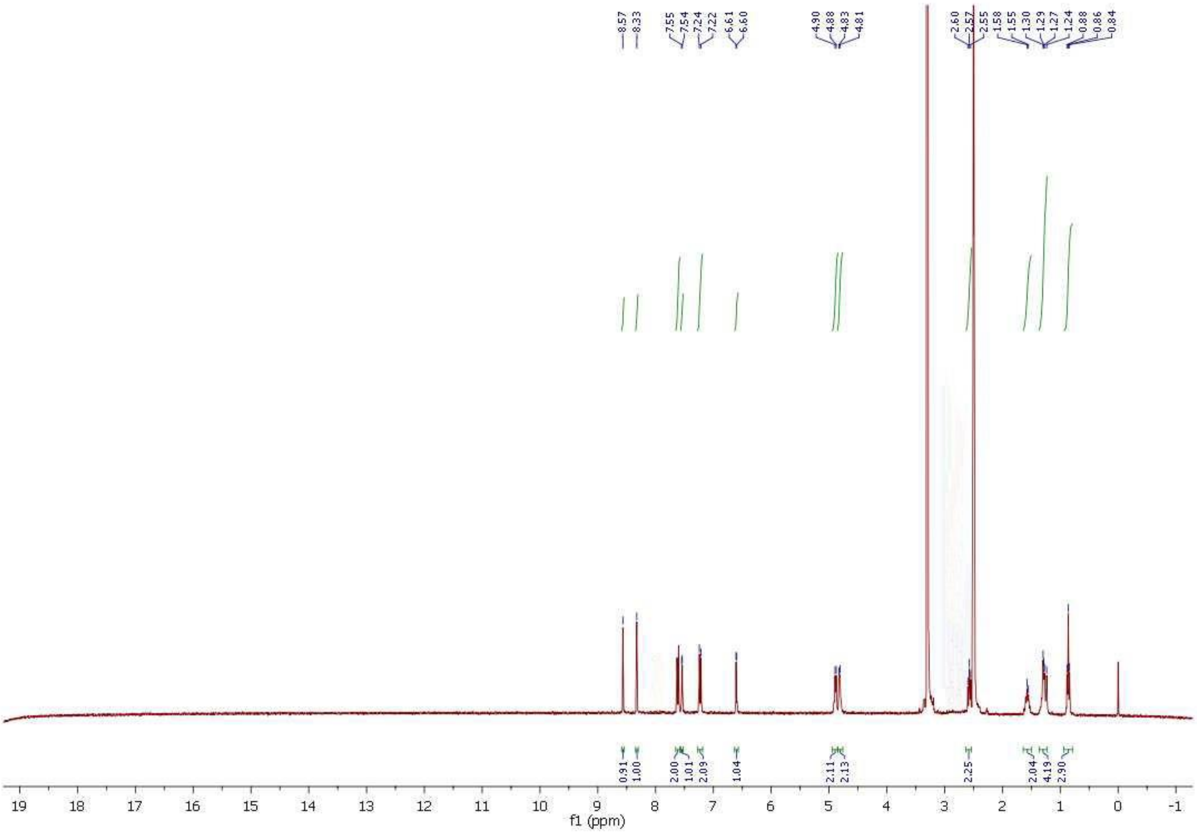

b)

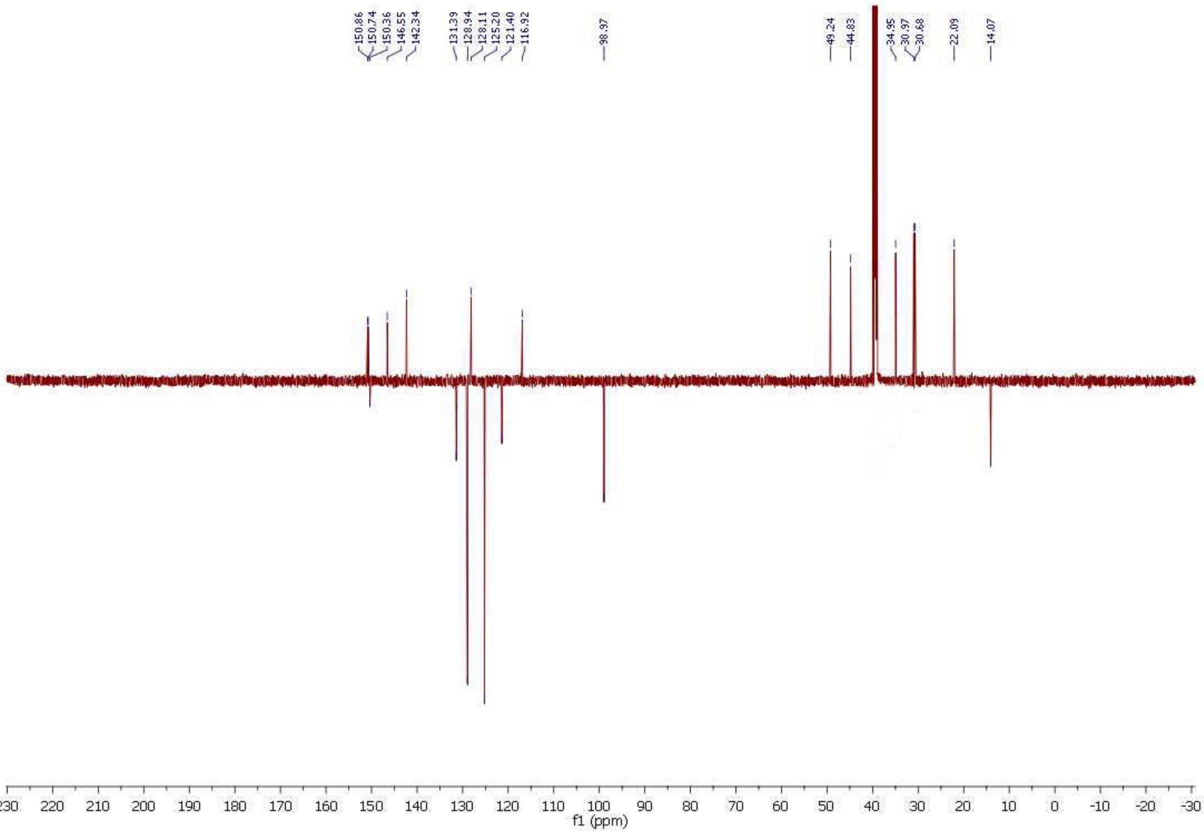

Figure S13. a) ^{1}H NMR and b) ^{13}C NMR of compd. **4h**

a)

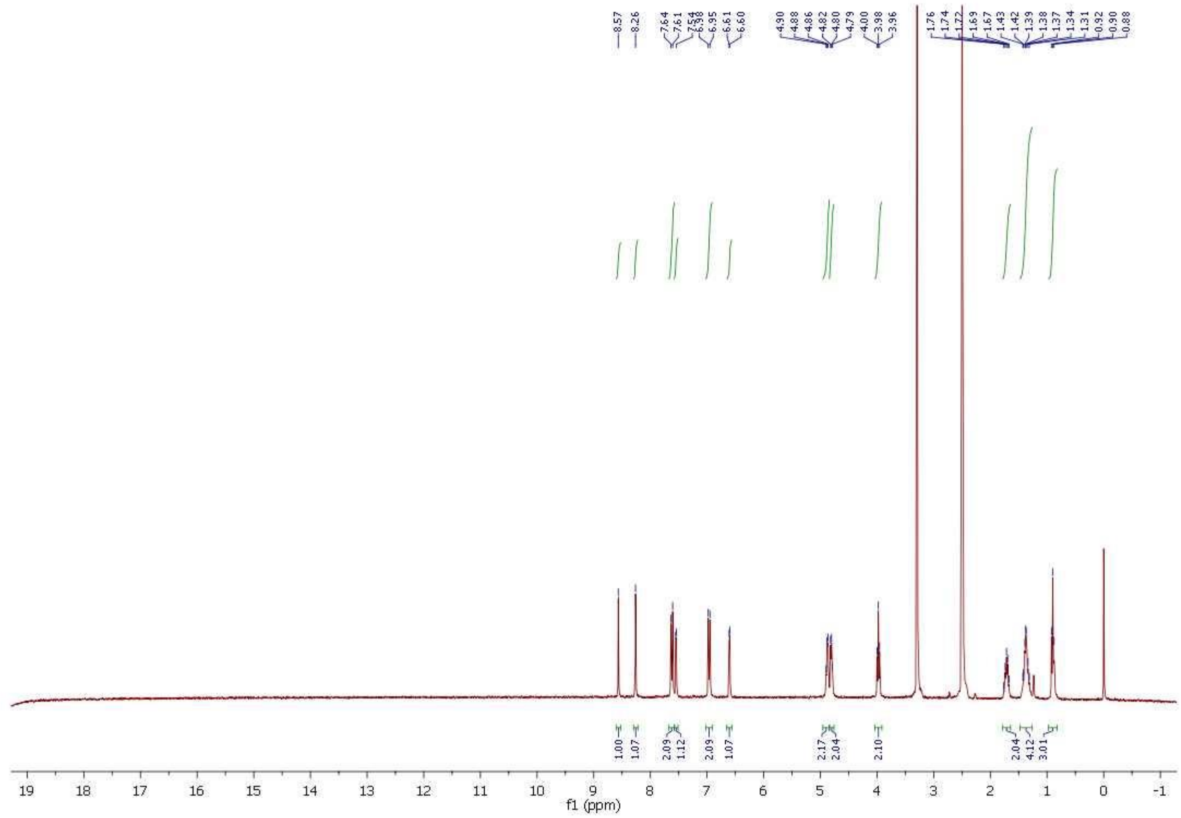

b)

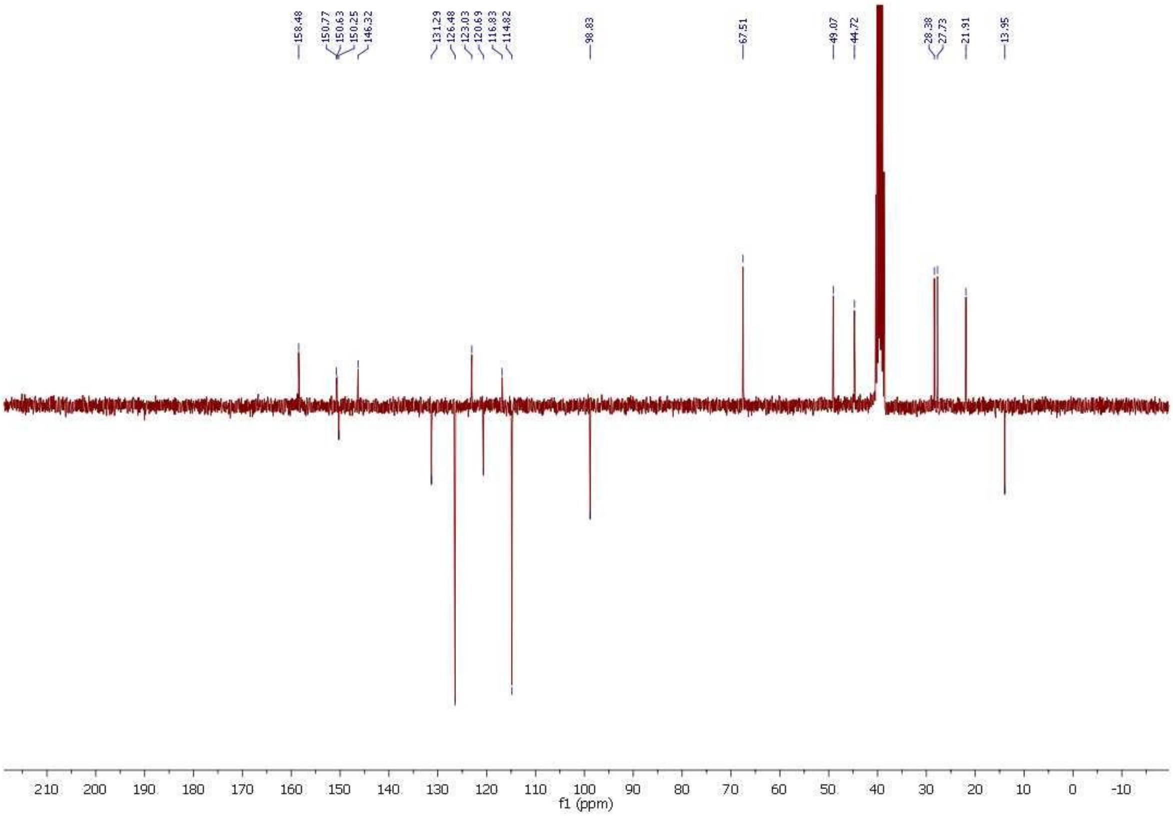

Figure S14. a) ^{1}H NMR and b) ^{13}C NMR of compd. **4i**

a)

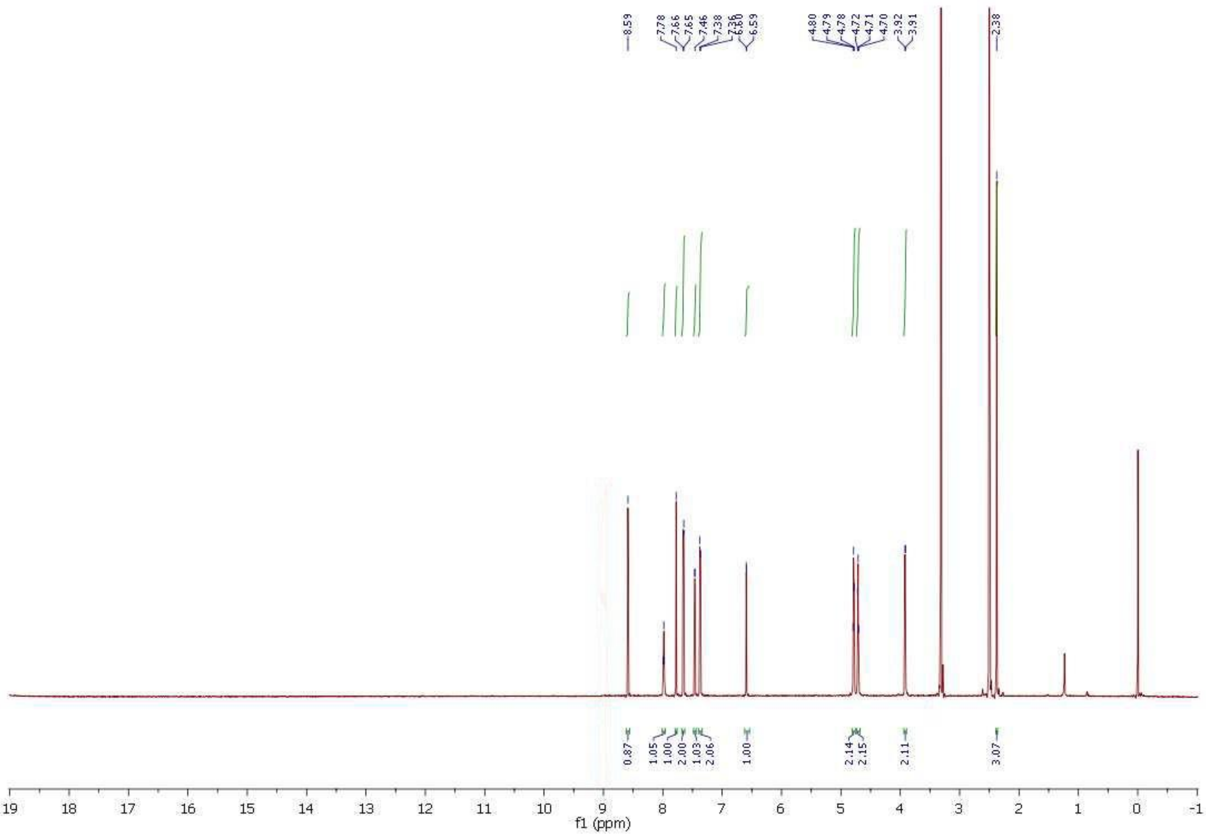

b)

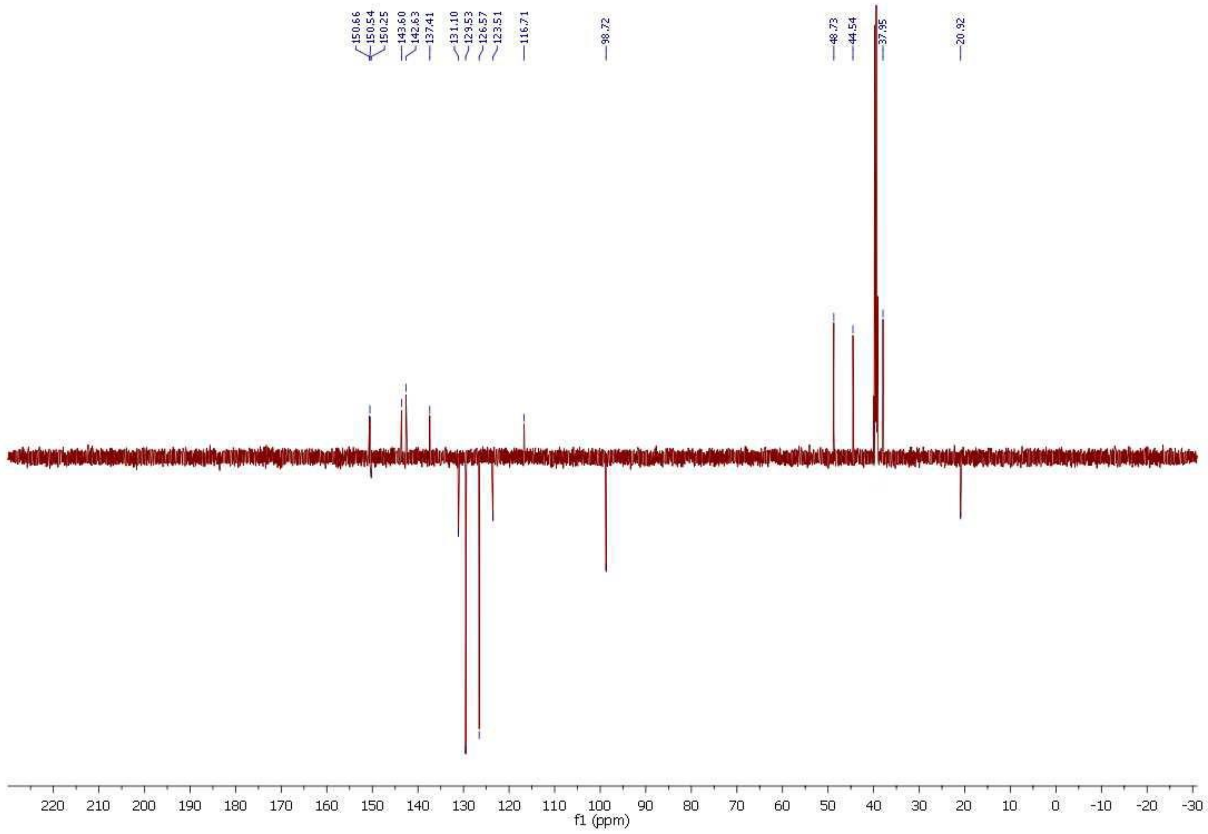

Figure S15. a) ^{1}H NMR and b) ^{13}C NMR of compd. **4j**

a)

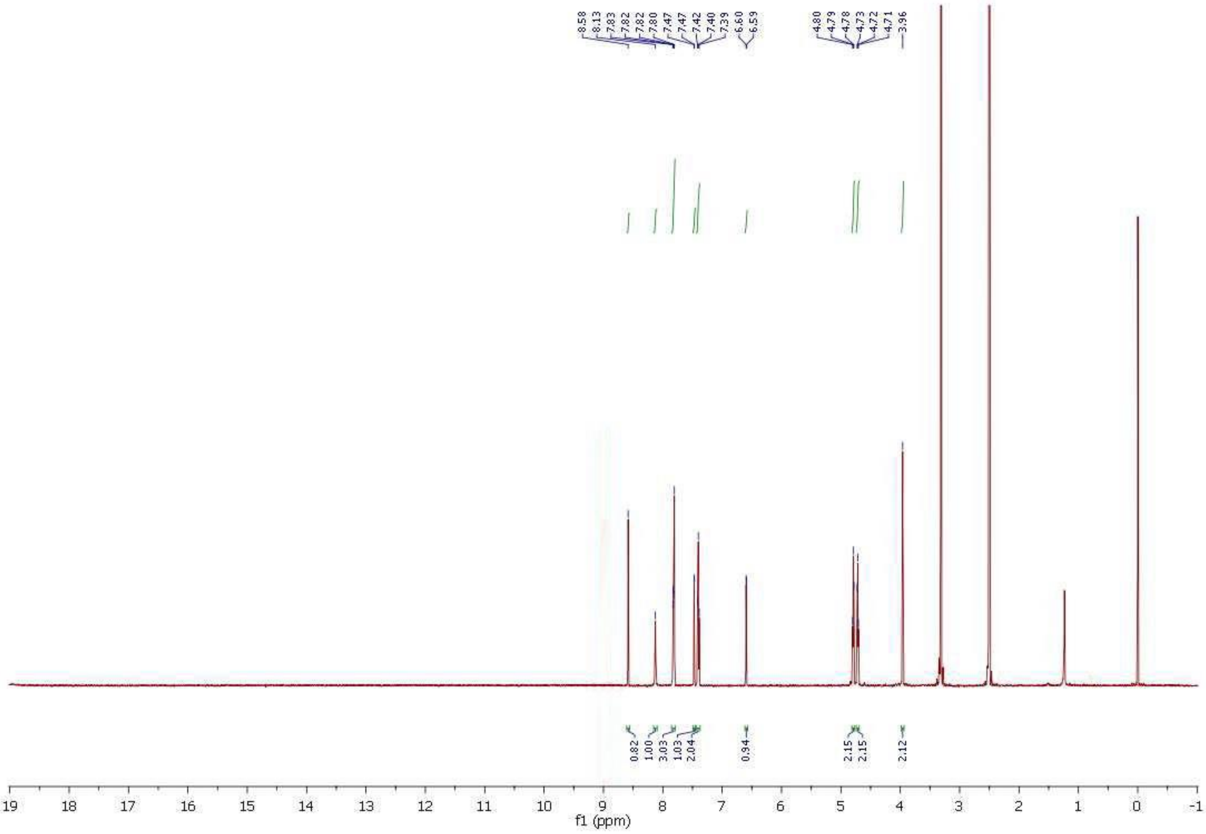

b)

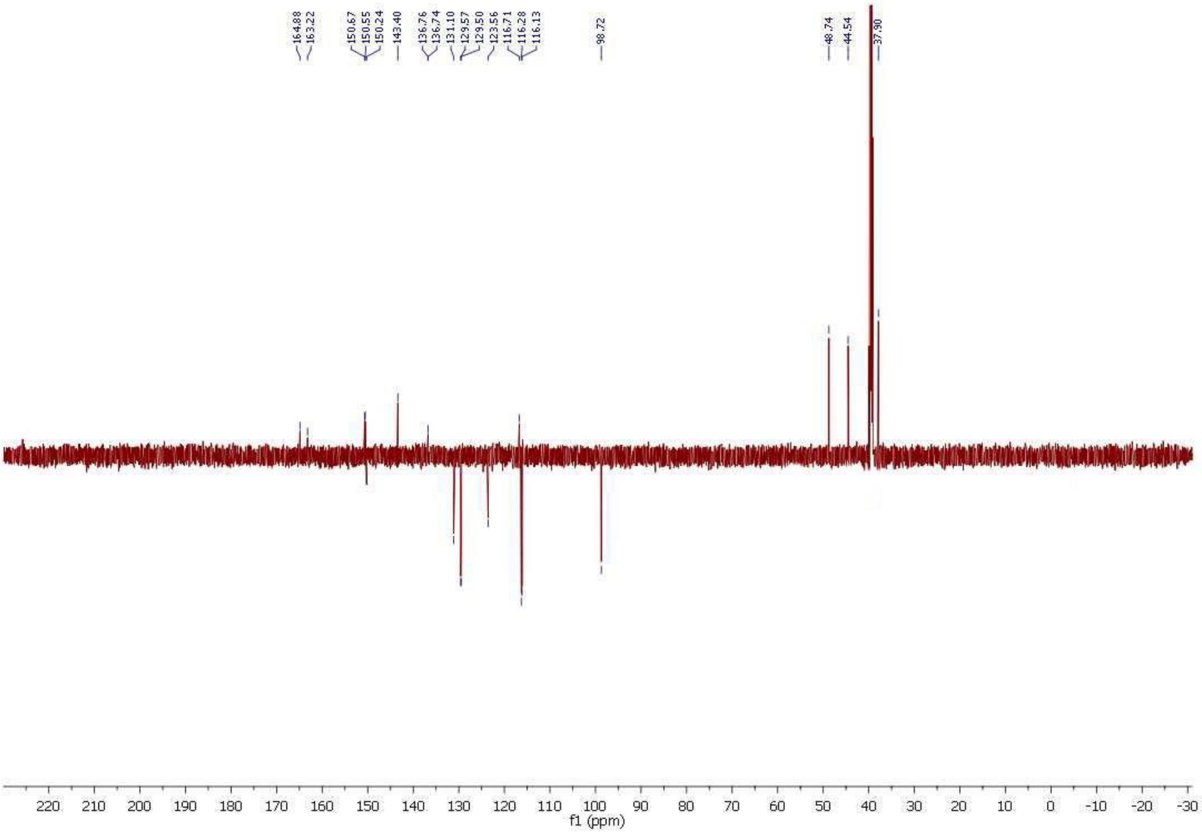

Figure S16. a) ^{1}H NMR and b) ^{13}C NMR of compd. **4k**

a)

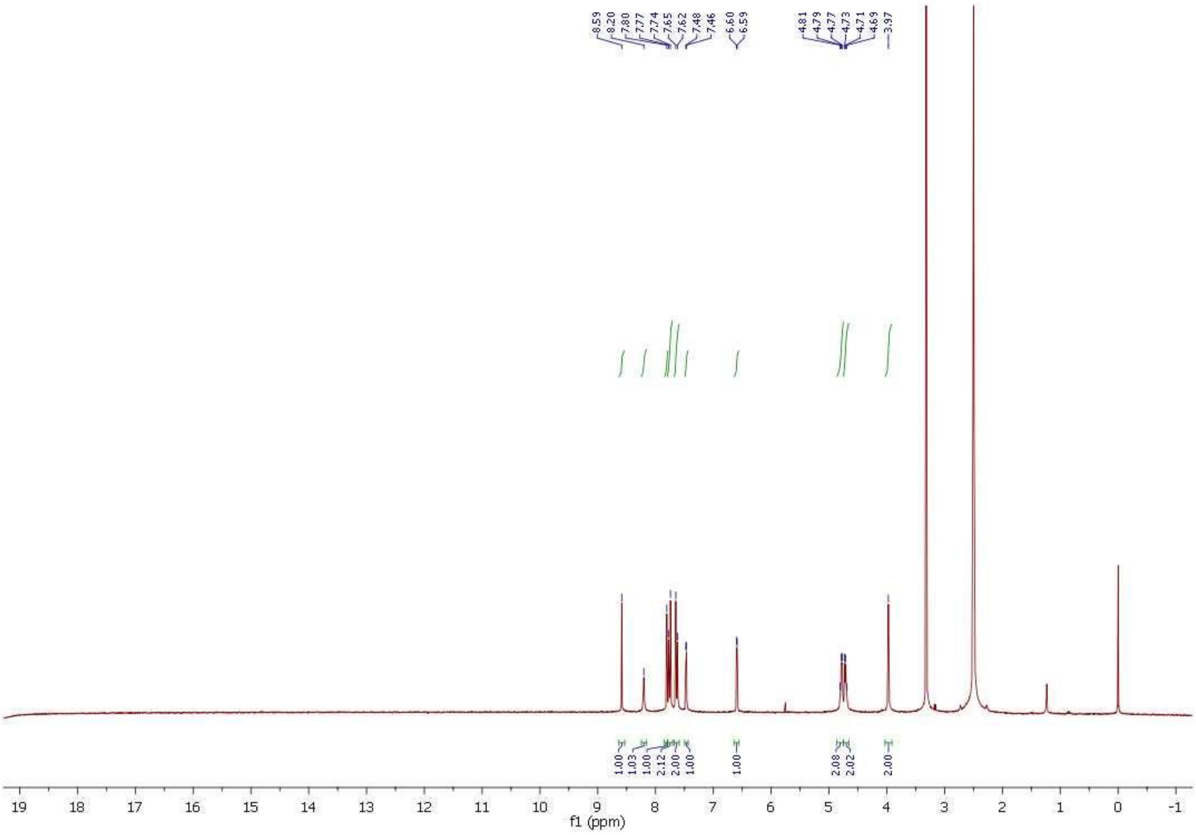

b)

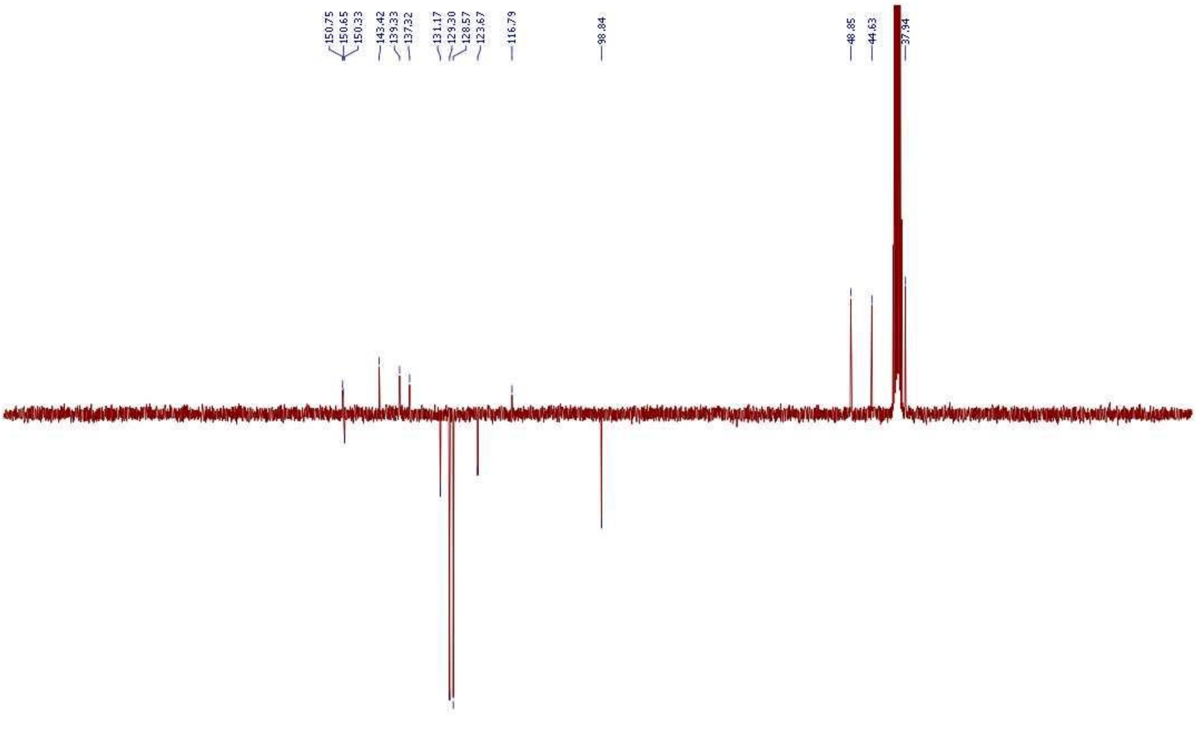

Figure S17. a) ^{1}H NMR and b) ^{13}C NMR of compd. **5a**

a)

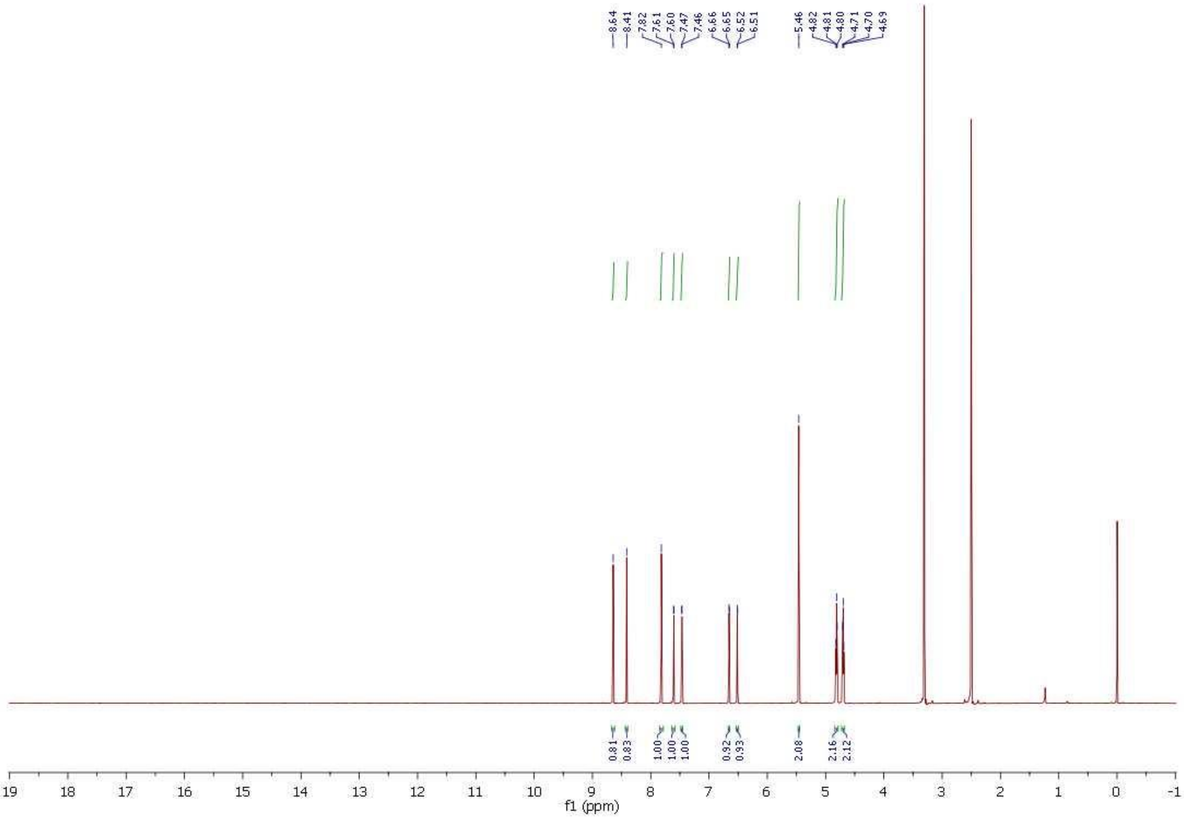

b)

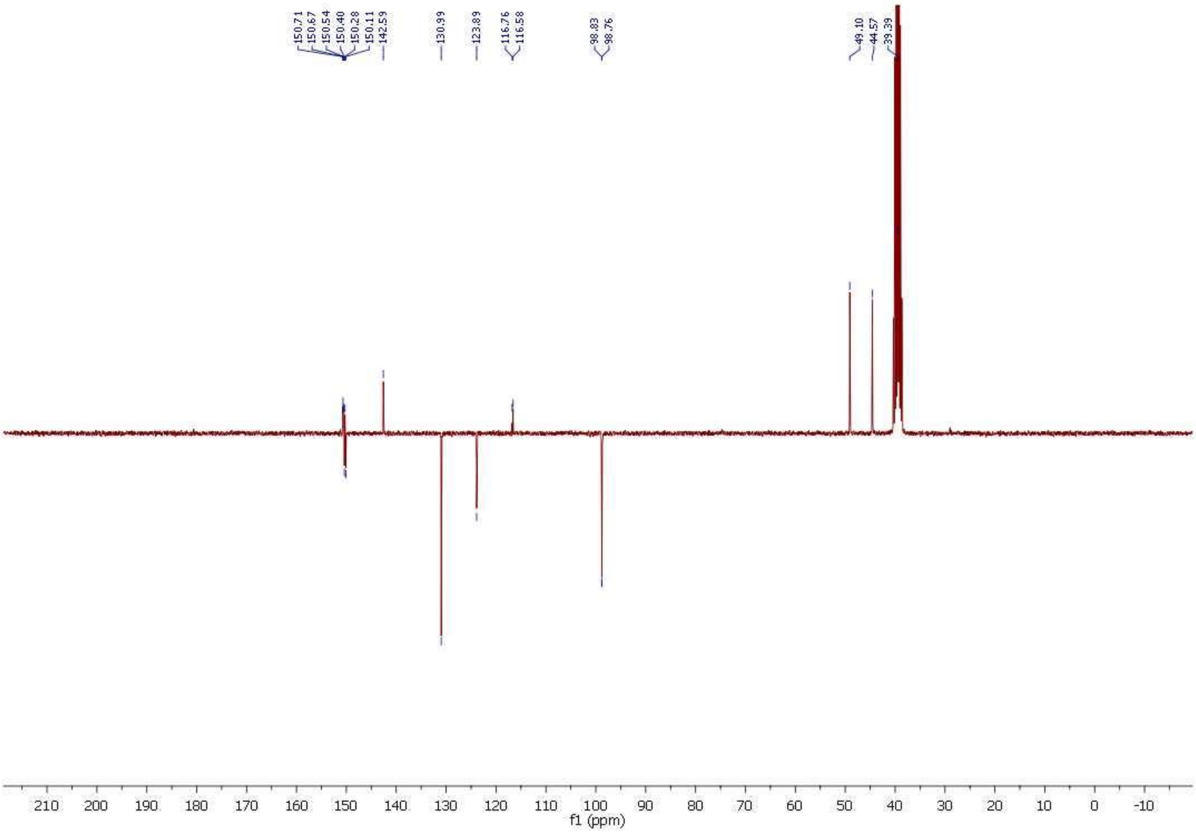

Figure S18. a) ^{1}H NMR and b) ^{13}C NMR of compd. **5b**

a)

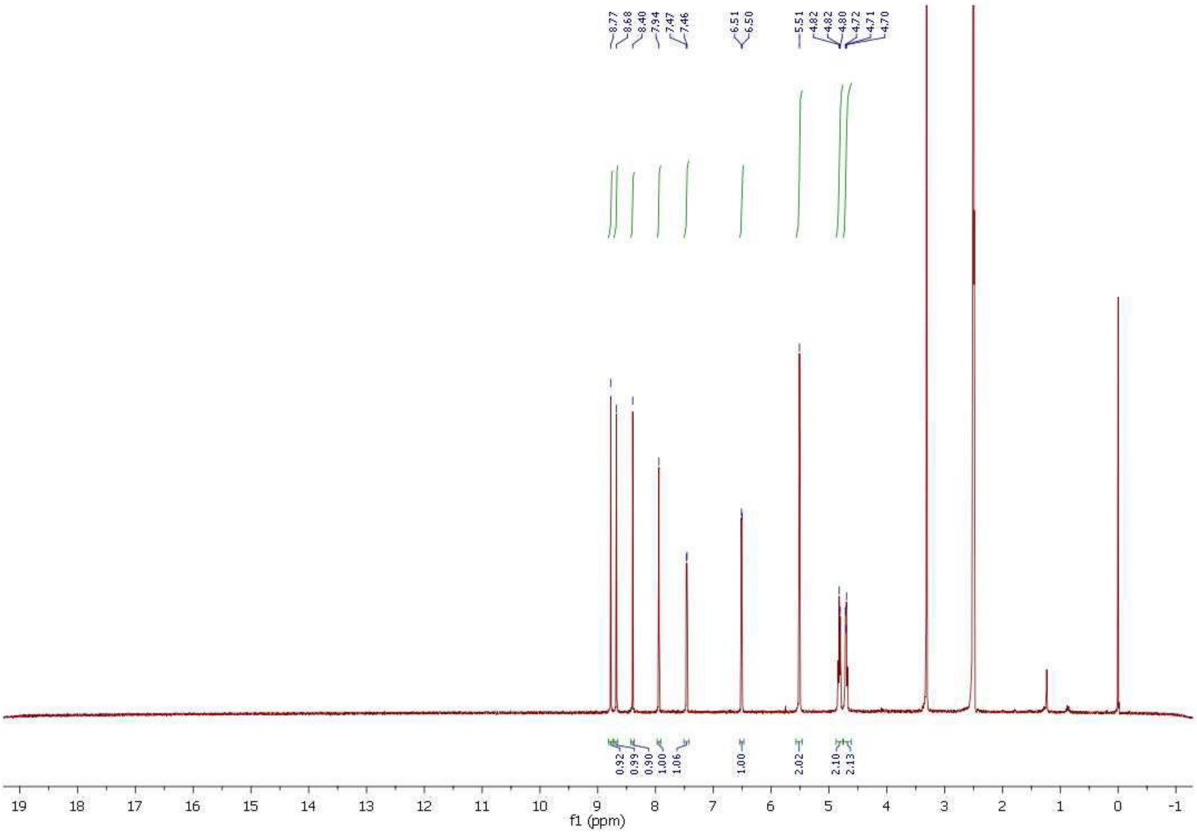

b)

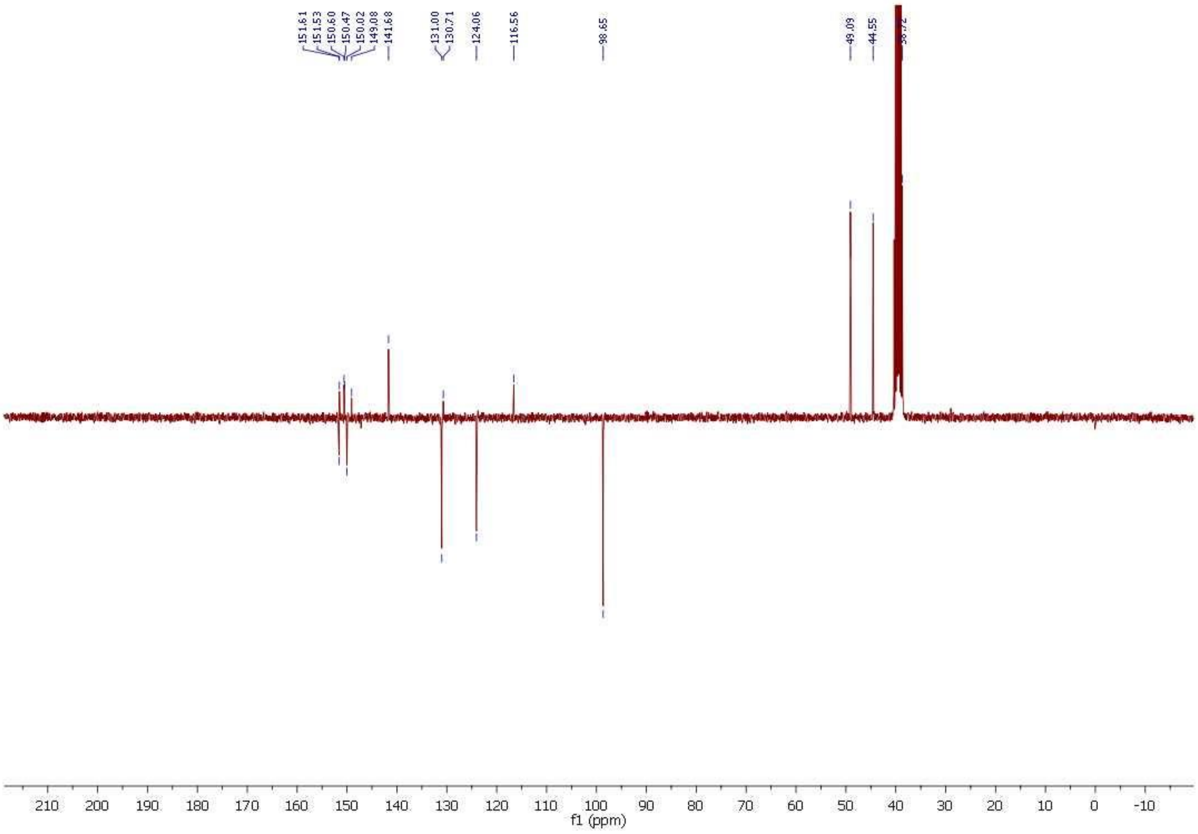

Figure S19. a) ^1H NMR and b) ^{13}C NMR of compd. **5c**

a)

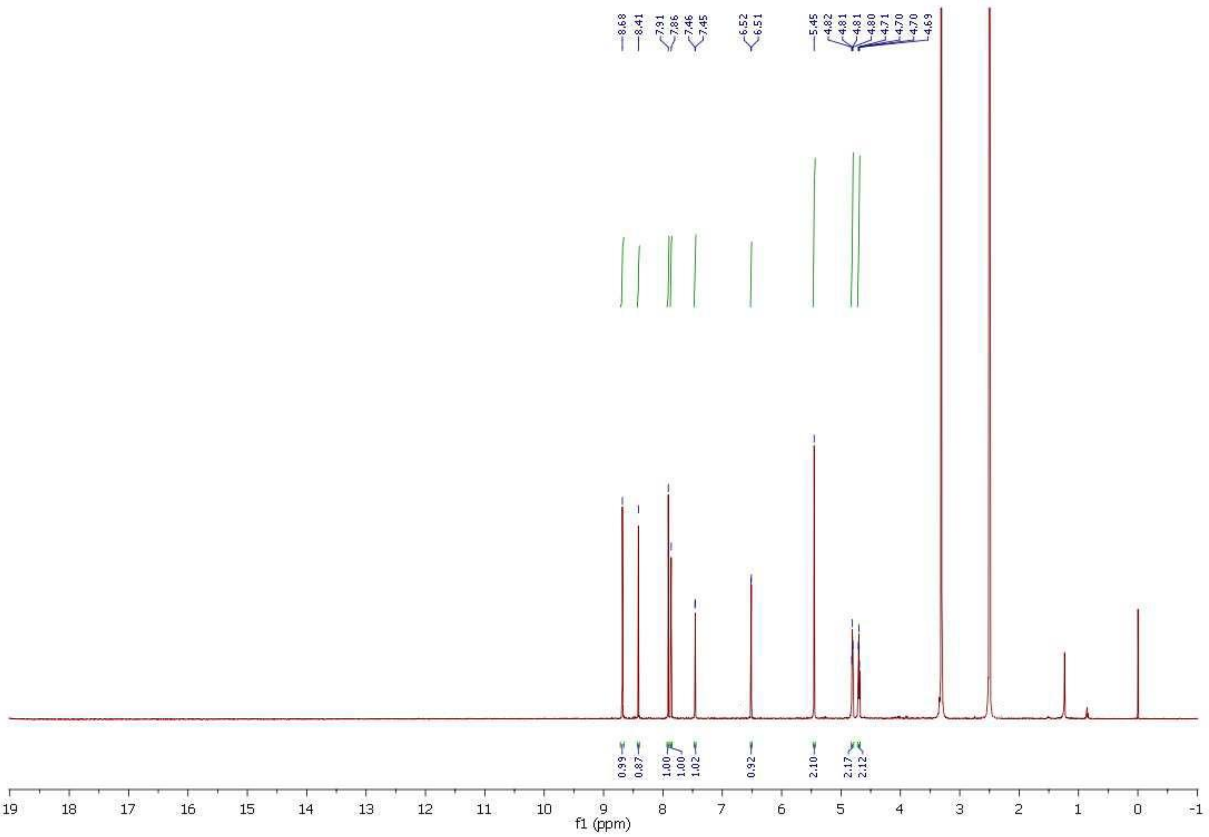

b)

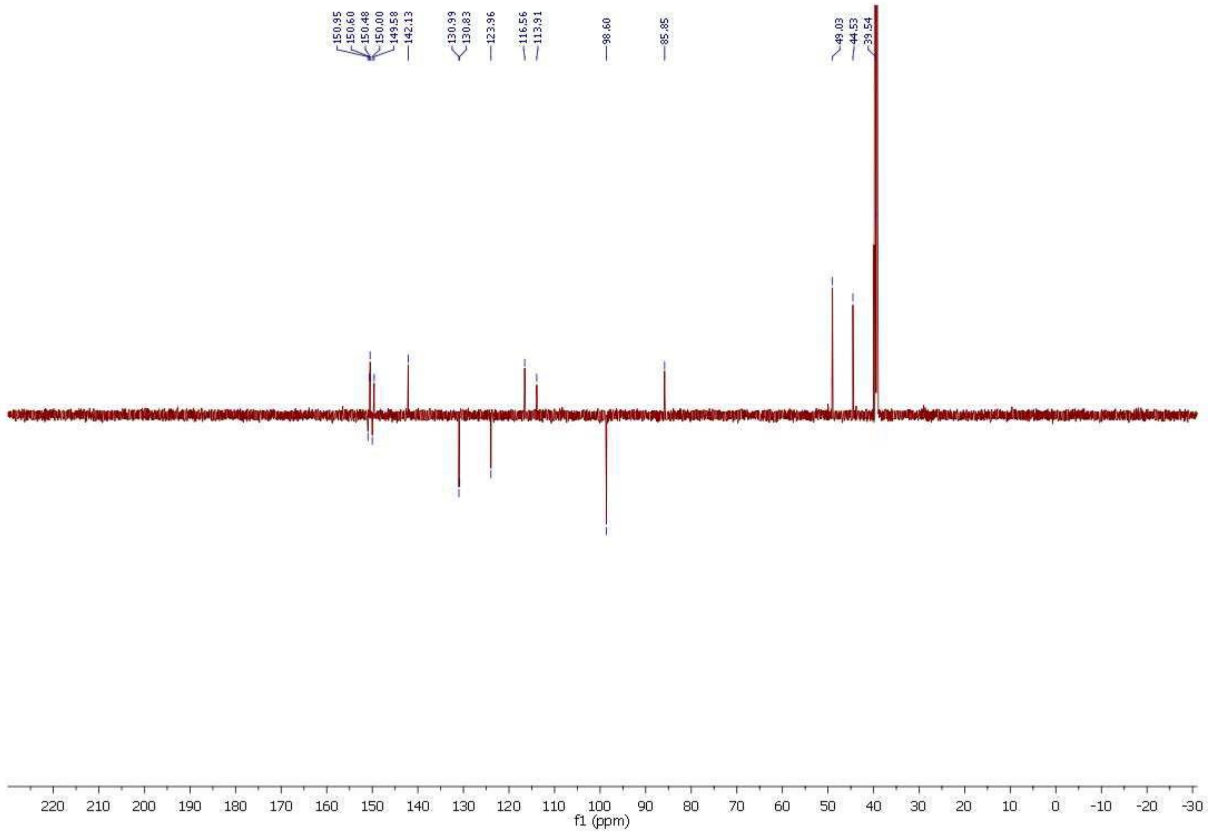

Figure S20. a) ^{1}H NMR and b) ^{13}C NMR of compd. **5d**

a)

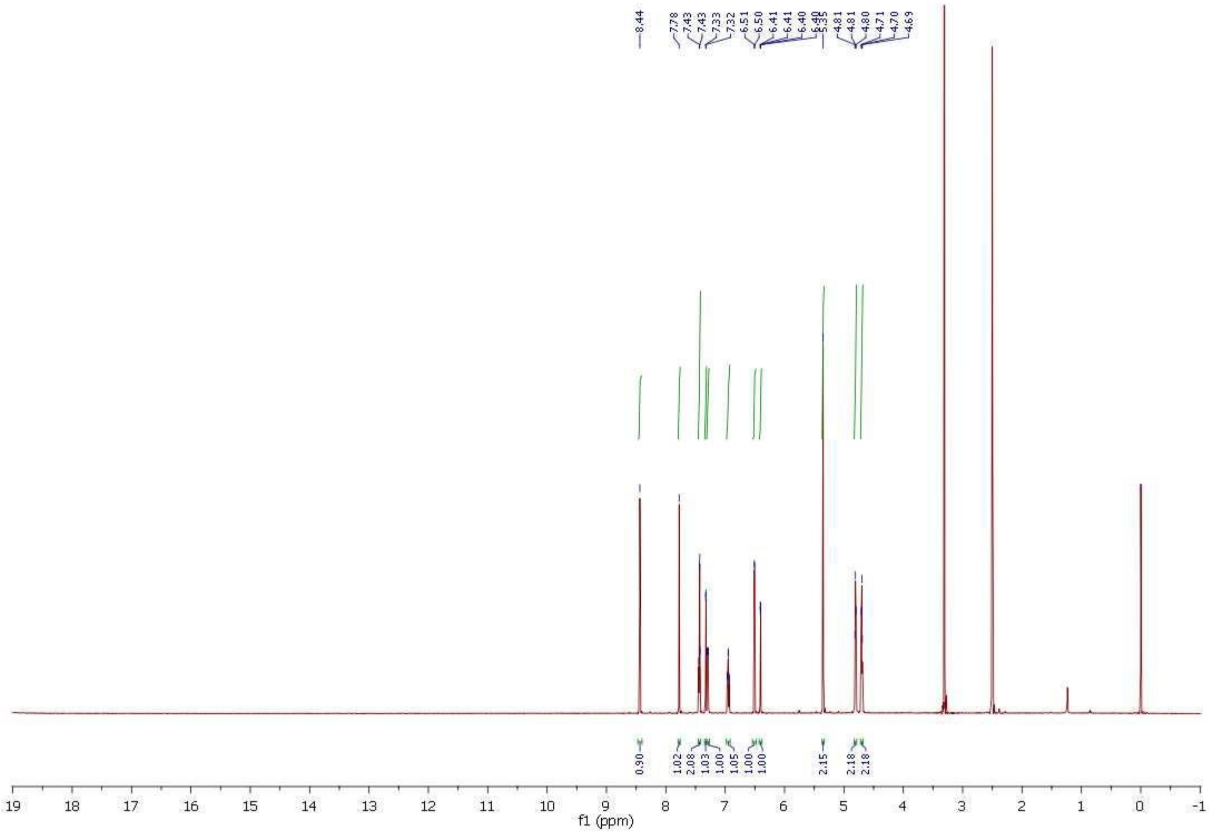

b)

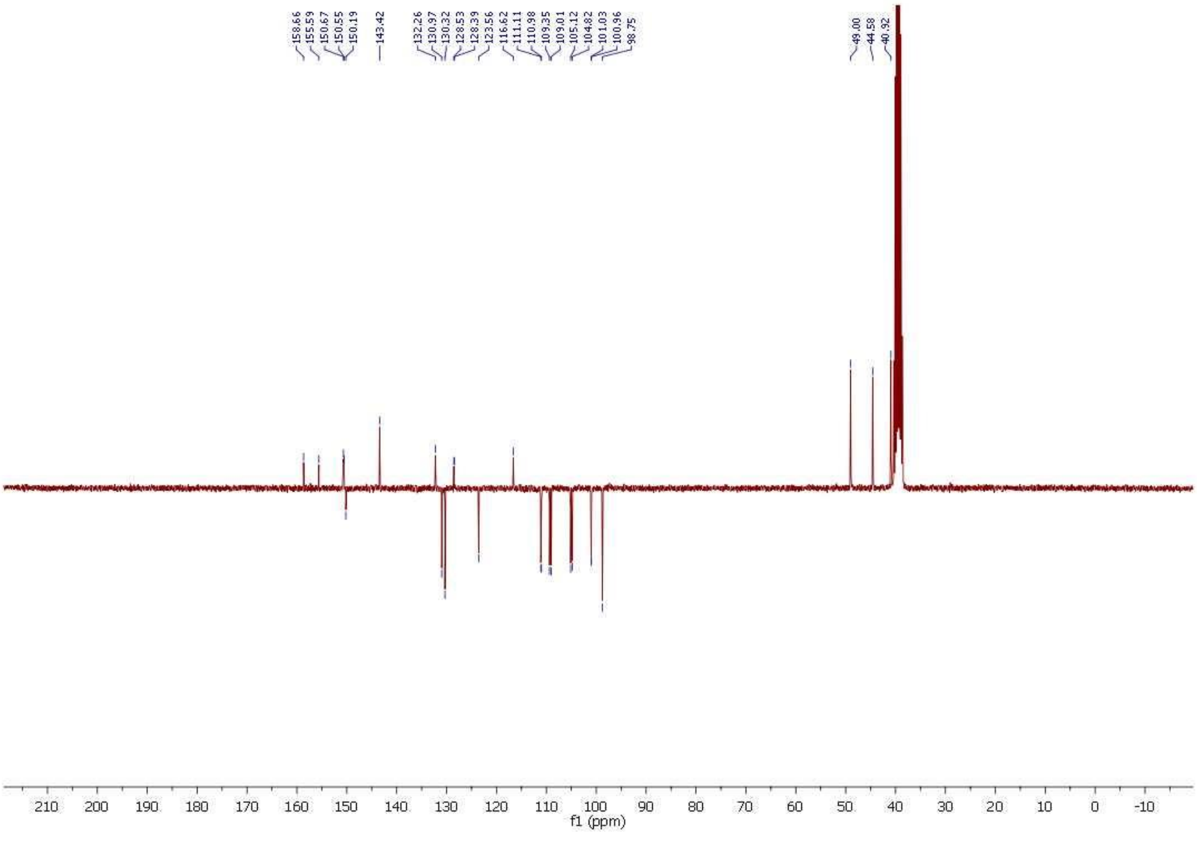

Figure S21. a) ^{1}H NMR and b) ^{13}C NMR of compd. **5e**

a)

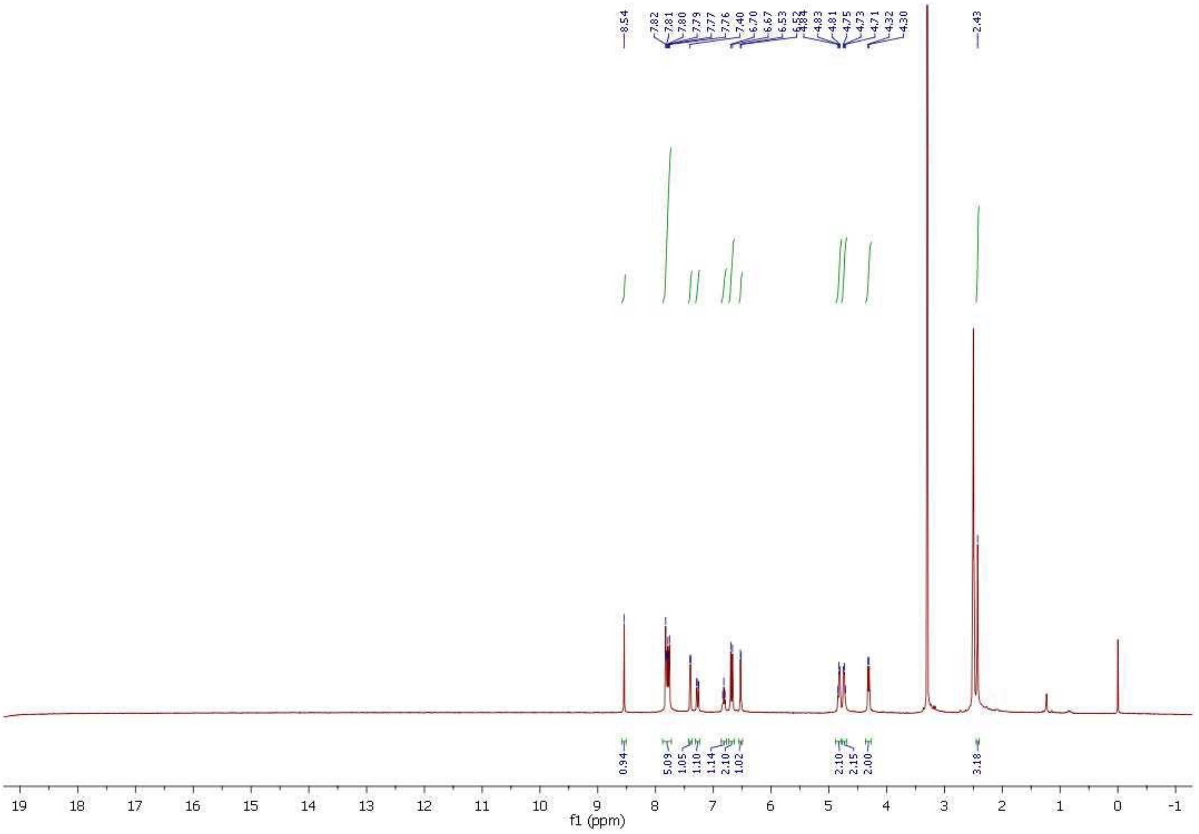

b)

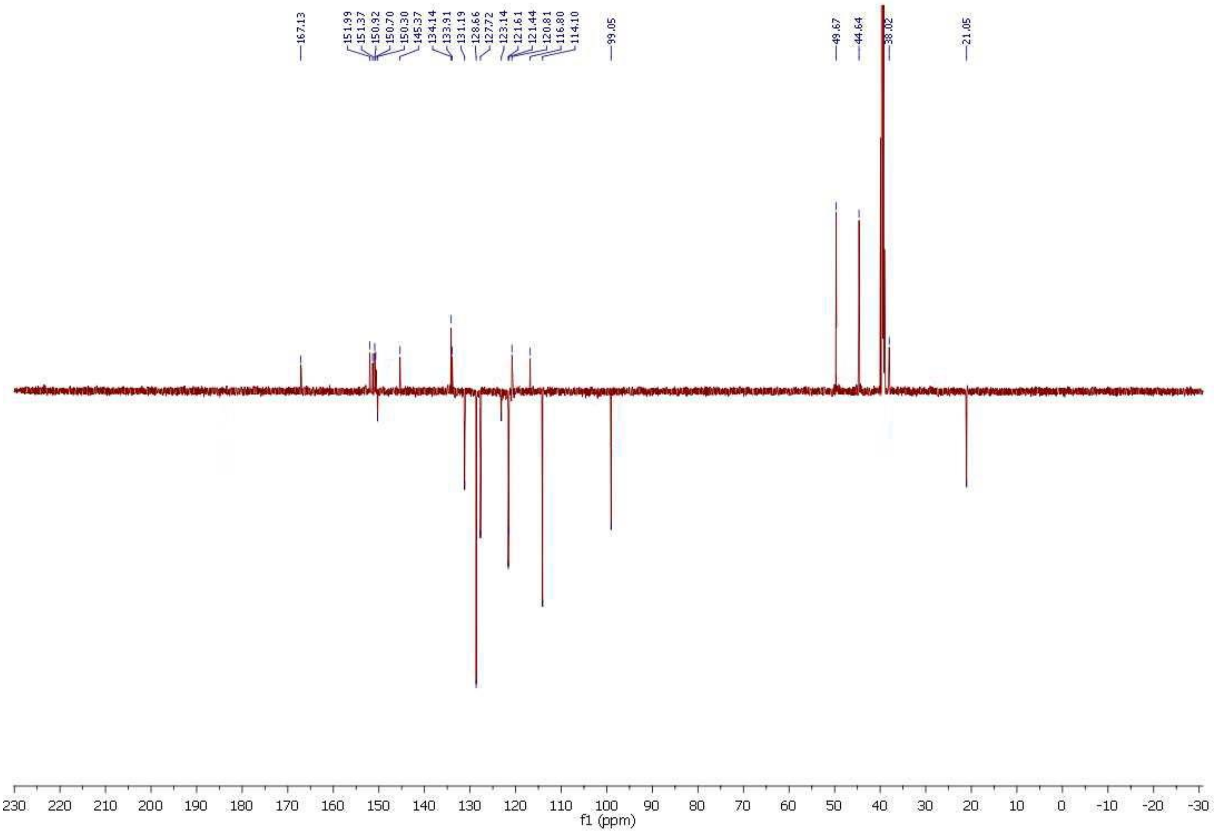

Figure S22. a) ^{1}H NMR and b) ^{13}C NMR of compd. **6a**

a)

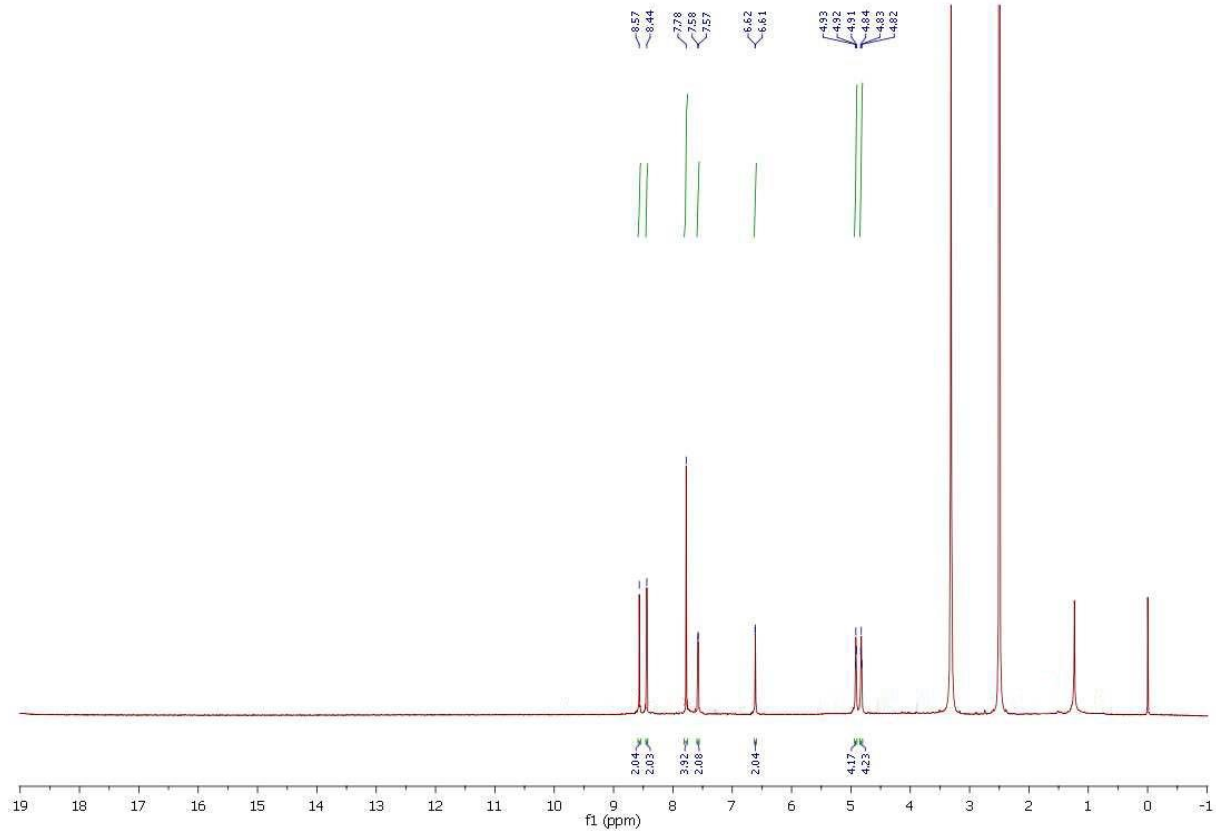

b)

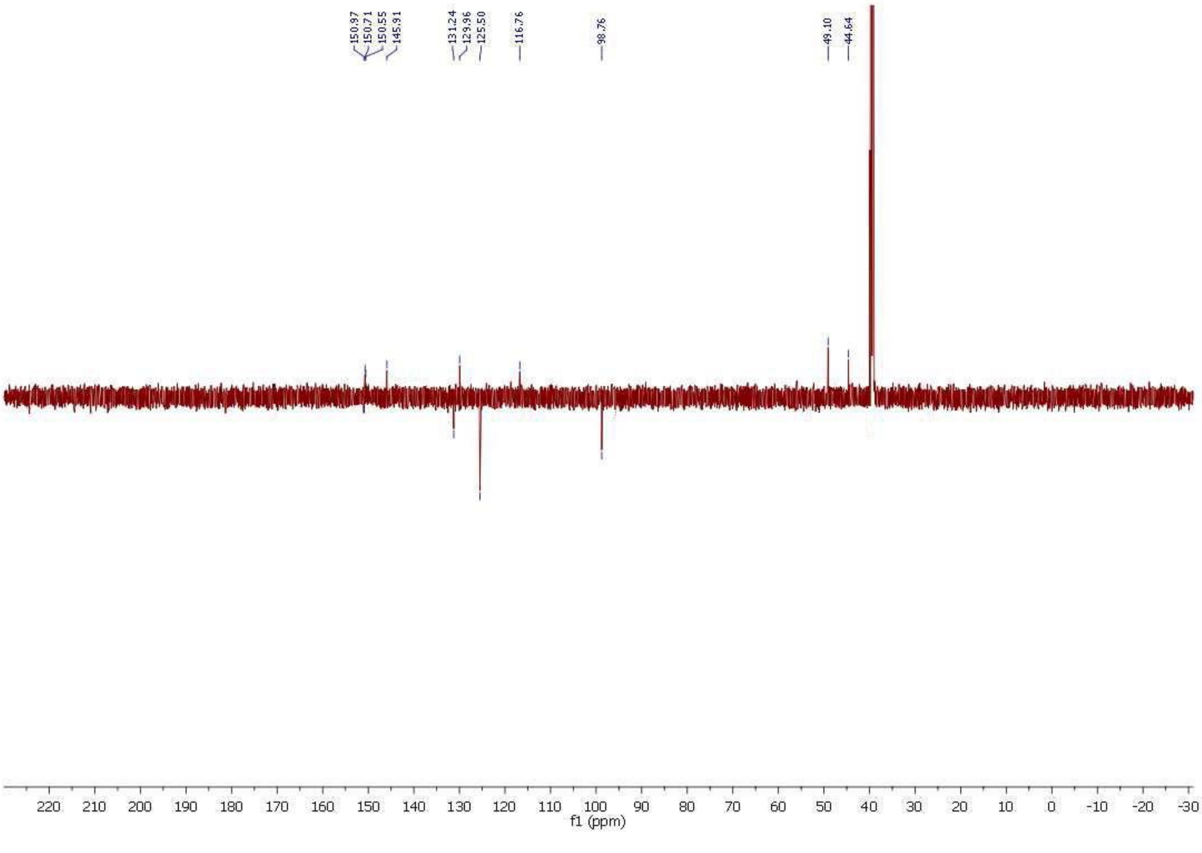

Figure S23. a) ^{1}H NMR and b) ^{13}C NMR of compd. **6b**

a)

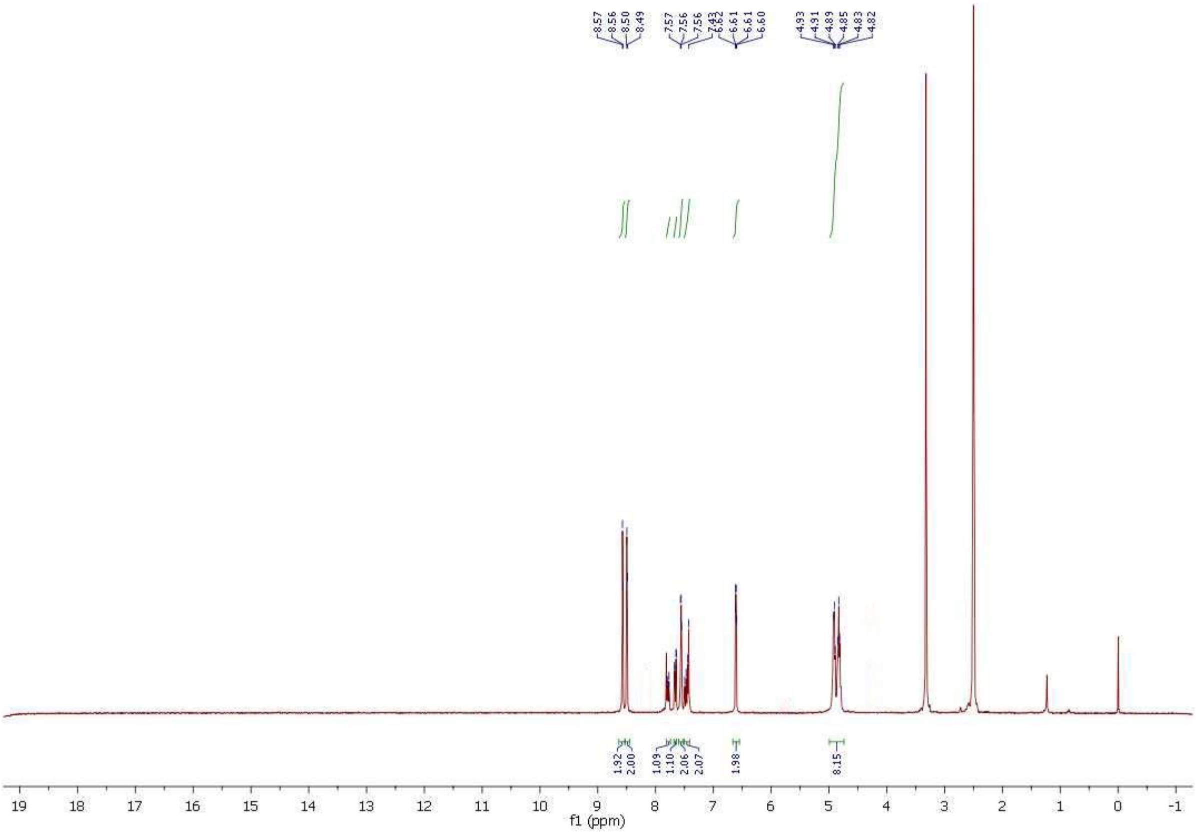

b)

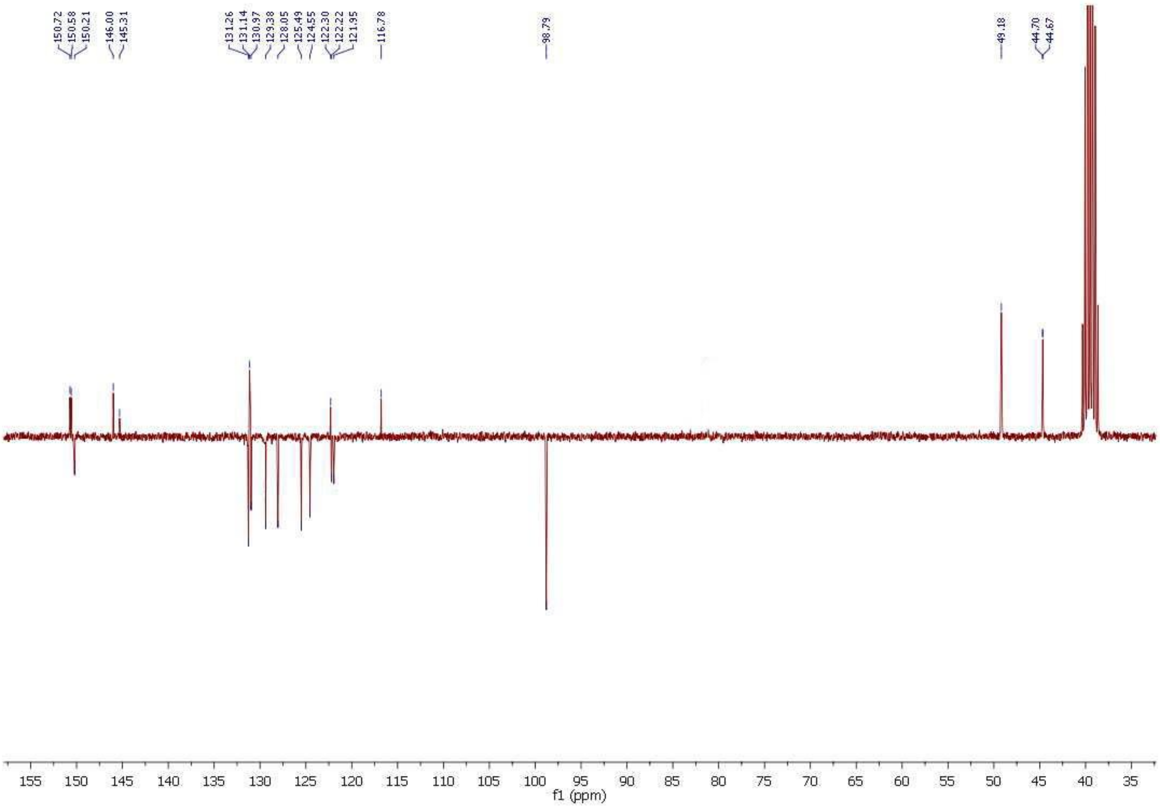

Supplement: Supplementary file 1 [file ijms-18-02292-s001.pdf]
